# Supplementary material for: Movement patterns and activity levels are shaped by the neonatal environment in Antarctic fur seal pups
Source: Sci Rep. 2021 Jul 12;11:14323. doi: 10.1038/s41598-021-93253-1 (PMC8275608; doi:10.1038/s41598-021-93253-1)
Supplement: Supplementary file 1 — Supplementary Information 1. [file 41598_2021_93253_MOESM1_ESM.pdf]

R-code for ‘Movement patterns and activity levels are shaped by the neonatal environment in Antarctic fur seal pups’

compiled by S. Mews and R. Nagel

Contents

Download packages and libraries **2**

5-minute GPS data **2**

Fitting 2 and 3 state HMMs 4

Hourly GPS data **16**

Determine the number of HMM states - 2 vs 3 state HMMs without covariates 18

2 state HMM - include covariates 29

Overview of final dataset **53**

Post-hoc analysis **55**

Pup mortality 56

Attendance of mother 57

Manuscript Figures **60**

Figure 2 60

Figure 3 64

Figure 4 68

Manuscript Figures - Supplementary information **70**

Figure S1 70

Figure S2 70

Figure S4 72

Figure S5 73

This document provides all the R code for the manuscript titled Movement patterns and activity levels are shaped by the neonatal environment in Antarctic fur seal pups by Rebecca Nagel, Sina Mews, Timo Adam, Claire Stainfield, Cameron Fox-Clarke, Camille Toscani, Roland Langrock, Jaume Forcada, and Joseph I.

Hoffman. Both the R Markdown file and the data can be downloaded via DRYAD (XXXX). If you have any questions, don't hesitate to contact Rebecca Nagel (renagel2@gmail.com).

The GPS data was collected from Antarctic fur seal (*Arctocephalus gazella*) on Bird Island, South Georgia between 2018-2020.

Download packages and libraries

In order to repeat analyses presented in this manuscript a number of packages that extend the functionalities of base `R` are required. These can be installed using the code `install.packages("xxPACKAGENAMExx", dependencies = TRUE)`

```
library(moveHMM)
library(dplyr)
library(lme4)
library(boot)
library(sjPlot)
library(ggplot2)
library(ggtext)
library(ggspatial)
library(data.table)
library(tidyr)
library(ggpubr)
library(DHARMa)
library(sf)
library(chron)
library(rgdal)
library(rgeos)
library(patchwork)
library(gridExtra)
library(mvtnorm)
library(ggsn)
```

5-minute GPS data

5-minute interval GPS data were collected from three fur seal pups, each between 30 – 50 days of age, for 13 – 19 days. To determine if there was any relevant loss of information on pup activity by using a coarser temporal resolution (hourly data), HMMs were fitted to these high frequency data.

```
### read in data
highFreq <- read.csv("Data5min.csv")
### data preparation
highFreq <- prepData(highFreq, coordNames = c("Longitude", "Latitude"))
highFreq$step <- highFreq$step * 1000
head(highFreq)
```

```
##    ID      step      angle         x         y       Date     Time Altitude
## 1 N21 15.425735         NA -38.05109 -54.00856 2020-01-19 09:52:01       12
## 2 N21 48.380353  0.5440309 -38.05087 -54.00861 2020-01-19 09:57:04        6
## 3 N21 52.284575  0.7339518 -38.05014 -54.00854 2020-01-19 10:02:09       24
## 4 N21 14.783983 -1.4217999 -38.04964 -54.00818 2020-01-19 10:07:11       24
## 5 N21  3.892253  0.3362293 -38.04945 -54.00824 2020-01-19 10:12:14       22
## 6 N21  7.448496 -2.3712435 -38.04939 -54.00825 2020-01-19 10:17:17       26
##   Satellites HDOP PDOP Temperature..C. Speed..km.h. TTFF SNR tbd
## 1          5  1.7  1.9              27        0.000   63   0   0
## 2          5  1.6  1.9              24        0.000    2   0   0
## 3          5  1.6  1.9              20        1.852    4   0   0
## 4          5  1.6  1.9              18       22.224    1   0   0
## 5          5  1.6  1.9              17        0.000    2   0   0
## 6          5  1.6  1.9              16        0.000    2   0   0
```

```
summary(highFreq$step)
```

```
##     Min.  1st Qu.   Median     Mean  3rd Qu.     Max.     NA's
##    0.000    7.151   13.829   25.471   27.202 1452.367      227
```

```
x <- which(highFreq$step > 200)
highFreq$step[x] <- highFreq$angle[x] <- highFreq$x[x] <- highFreq$y[x] <- NA

sum(highFreq$step == 0, na.rm = T)
```

```
## [1] 2
```

```
highFreq$step[which(highFreq$step == 0)] <- NA
# get an overview of the variables step length & turning angle
par(mfrow = c(2, 1))
hist(highFreq$step, breaks = 60, col = "grey", xlab = "step length in metres", main =
    "Histogram", prob = T)
hist(highFreq$angle, breaks = 60, col = "grey", xlab = "turning angle in radians", main =
    "Histogram", prob = T)
```

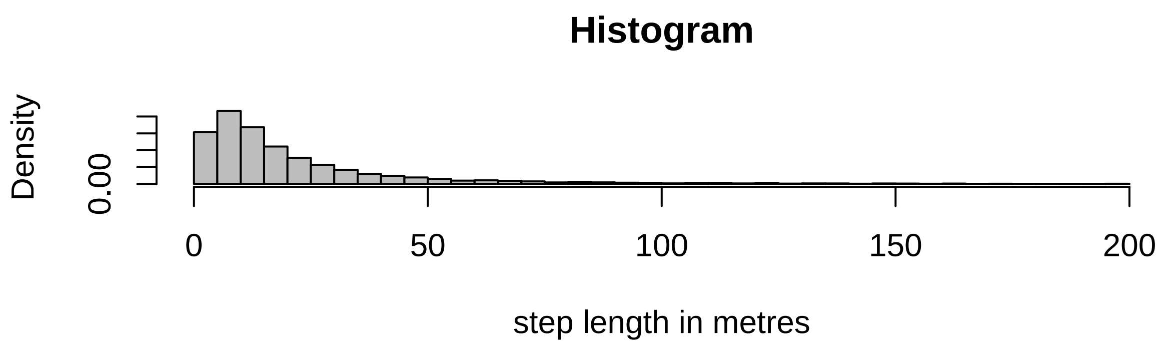

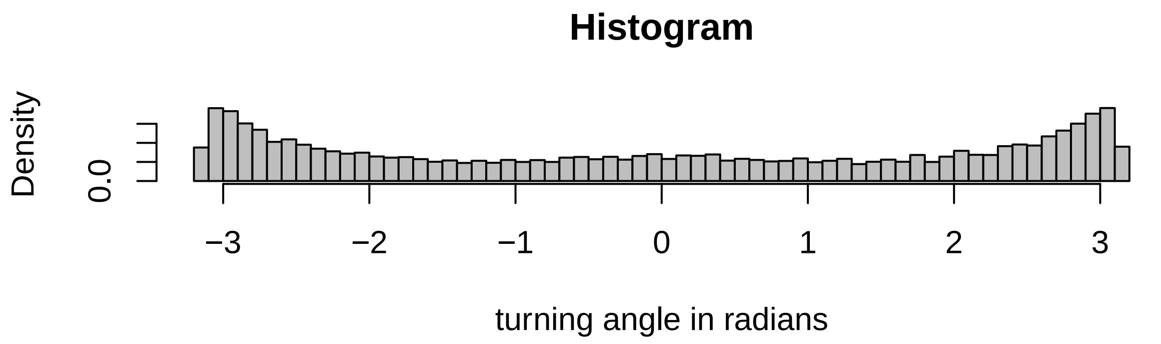


```
summary(highFreq$step)
```

```
##     Min.  1st Qu.   Median     Mean  3rd Qu.     Max.     NA's
##   0.1115   7.1049  13.6947  22.5767  26.7937 198.5270      314
```

```
summary(highFreq$angle)
```

```
##    Min. 1st Qu.  Median    Mean 3rd Qu.    Max.    NA's
## -3.1416 -2.1590 -0.0121 -0.0071  2.1373  3.1416     401
```

Fitting 2 and 3 state HMMs

2 and 3 state HMMs were fitted to the 5-minute interval data. Only models with a biologically meaningful numbers of states should be tested. We expected Antarctic fur seal pup movement to include a resting state (short step-lengths), an active state (long step-lengths with more directed movement) and potentially a local exploration state (moderate step-lengths with high degrees of turning).

```
### 2-state HMM initial parameter values
mu0 = c(8, 40)
sigma0 = c(5, 20)
kappa0 = c(0.5, 1)
stepPar0 = c(mu0, sigma0)
anglePar0 = c(-3, 0, kappa0)
```

```r
mod0 <- fitHMM(highFreq, nbStates = 2, stepDist = "gamma", angleDist = "vm", stepPar0 =
    stepPar0, anglePar0 = anglePar0, stationary = T, verbose = 0)
mod0
```

```
## Value of the maximum log-likelihood: -66188.86
##
## Step length parameters:
## ----------------------
##        state 1  state 2
## mean 11.629442 44.93999
## sd    8.256221 33.26303
##
## Turning angle parameters:
## ------------------------
##                  state 1    state 2
## mean          -3.1381353 -3.1206931
## concentration  0.5427501  0.4761463
##
## Regression coeffs for the transition probabilities:
## --------------------------------------------------
##              1 -> 2    2 -> 1
## intercept -2.452853 -1.673565
##
## Transition probability matrix:
## -----------------------------
##           [,1]      [,2]
## [1,] 0.9207698 0.0792302
## [2,] 0.1579495 0.8420505
##
## Initial distribution:
## --------------------
## [1] 0.6659486 0.3340514
```

```r
plot(mod0, animals = 1)
```

```
## Decoding states sequence... DONE
```

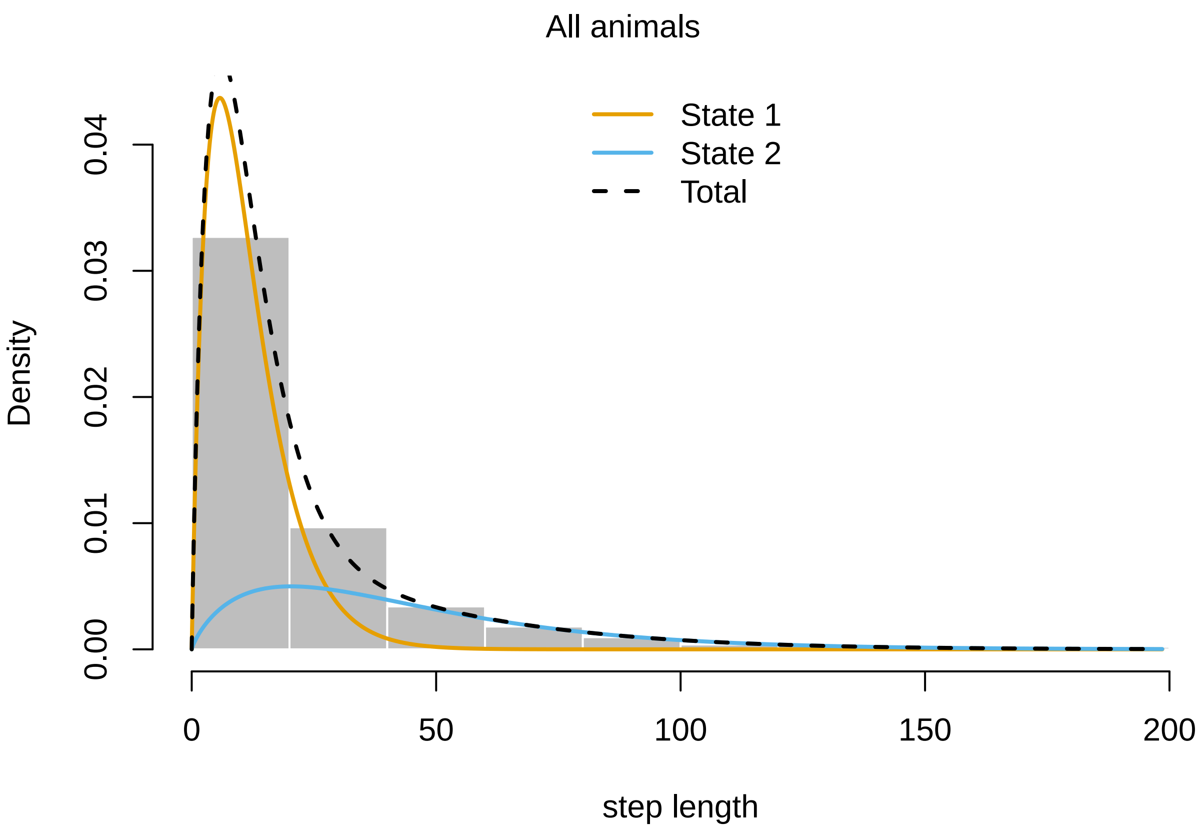
All animals
State 1
State 2
Total
Density
0.00
0.01
0.02
0.03
0.04
0
50
100
150
200
step length

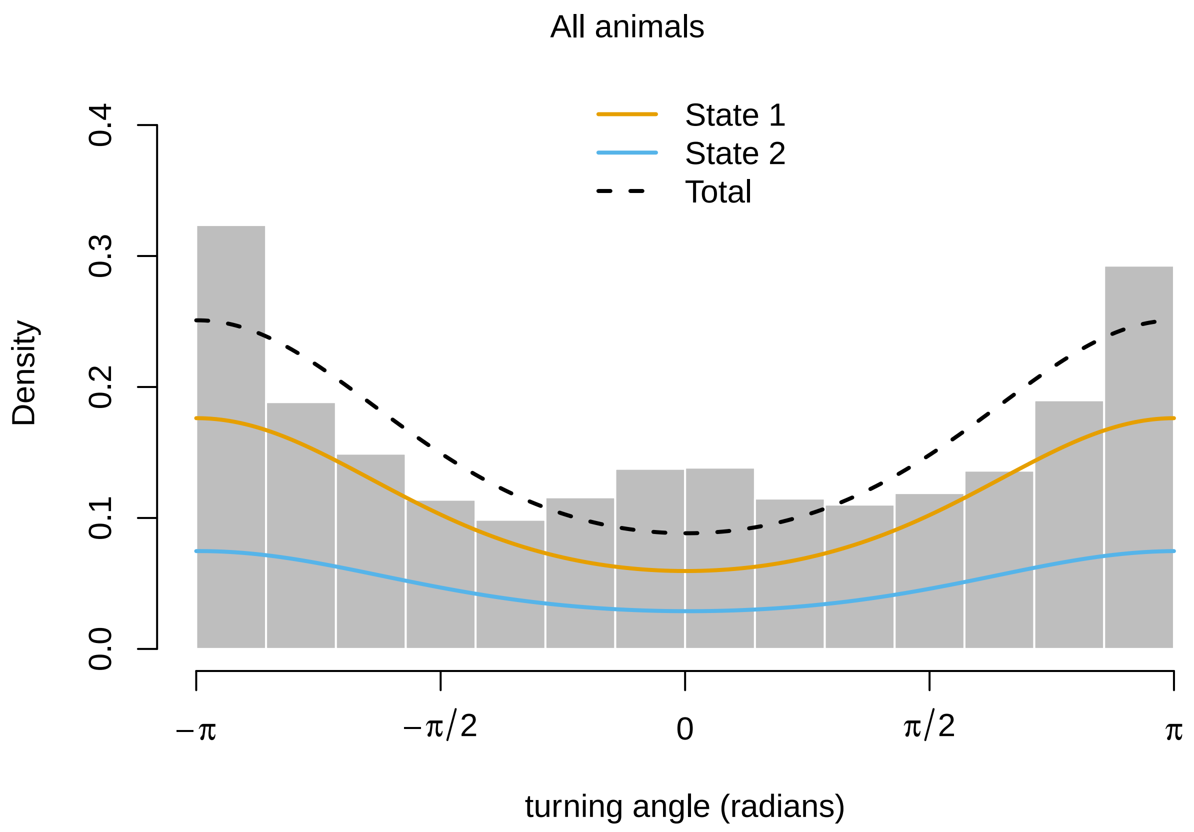
All animals
State 1
State 2
Total
Density
0.0
0.1
0.2
0.3
0.4
−π
−π/2
0
π/2
π
turning angle (radians)

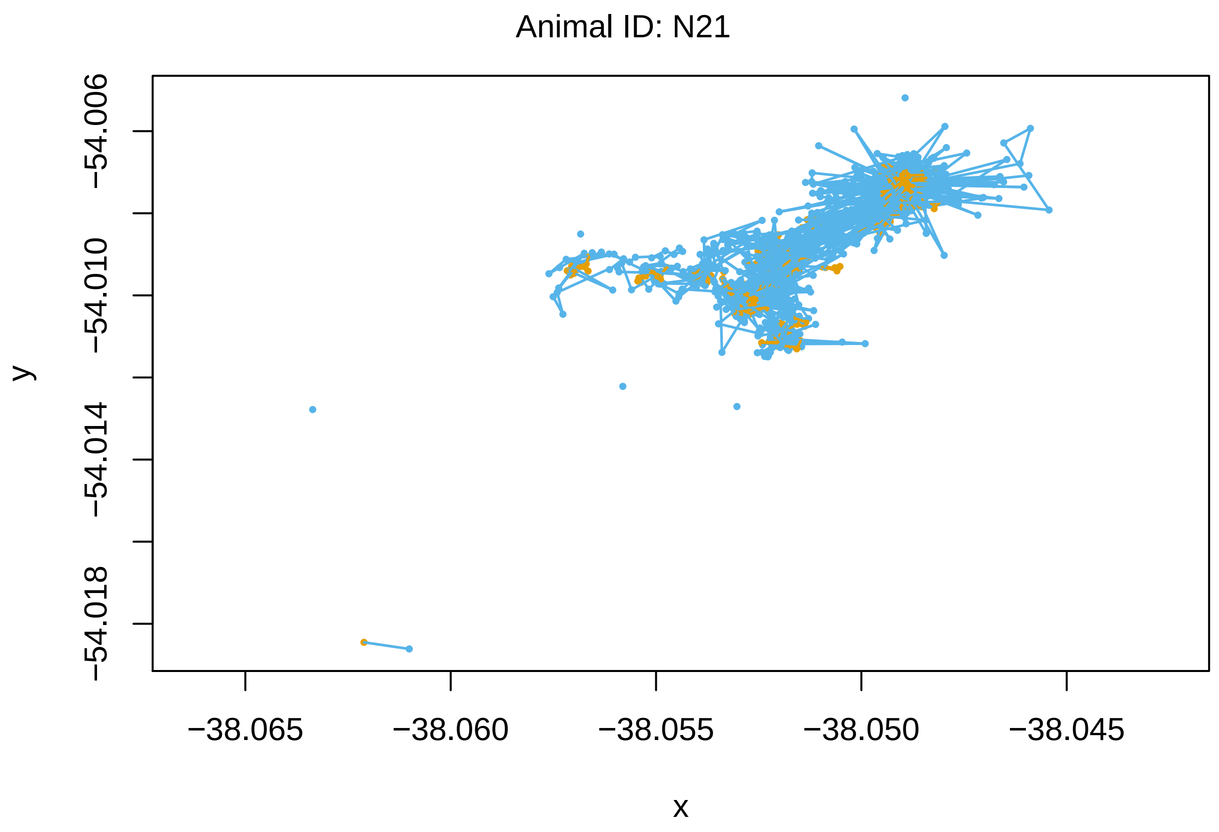


```r
# model checking using pseudo-residuals
plotPR(mod0)
```

```
## Computing pseudo-residuals... DONE
```

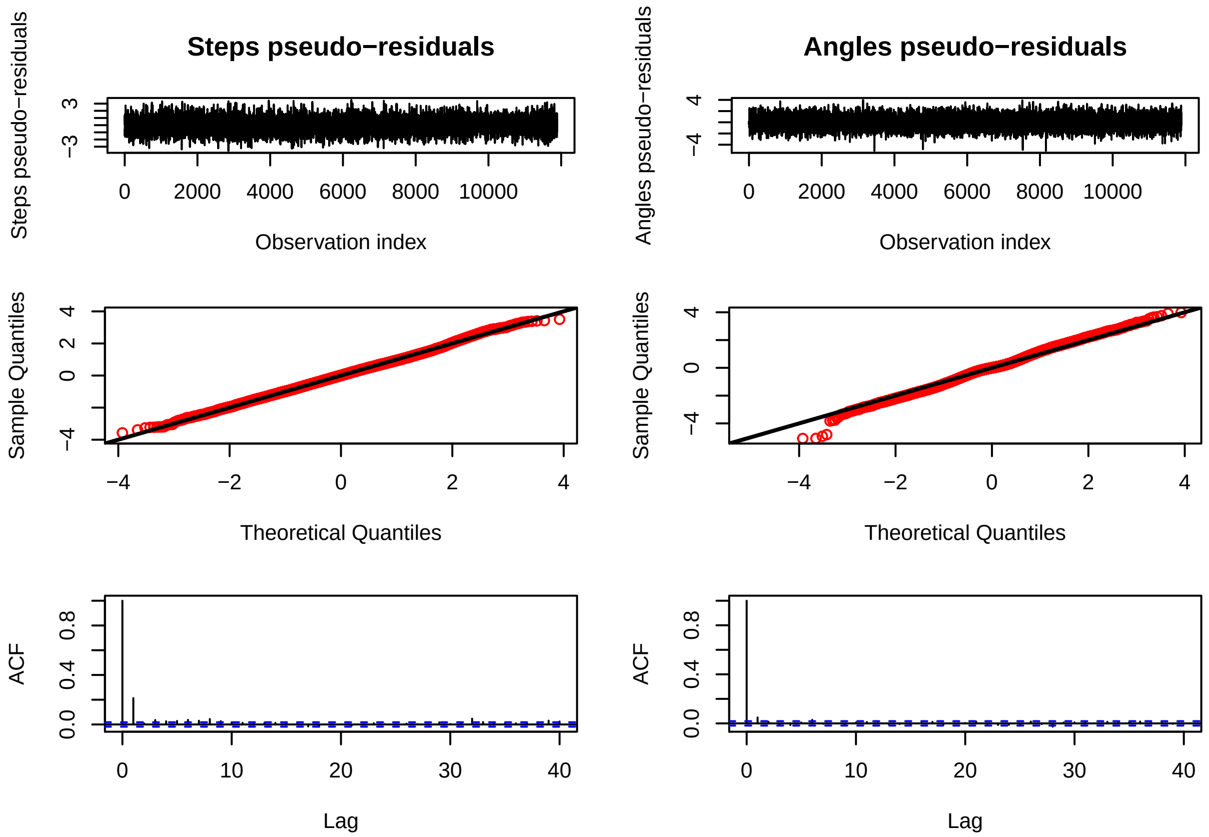


```
## code to fit several (2-state) HMMs using random starting values (to check for
## local maxima)
nb.fit <- 10 # number of models to fit
llks <- numeric(nb.fit)
mods <- list()
for (i in 1:nb.fit) { mu0 = runif(2, 10, 150) sigma0 = runif(2, 5, 100) mean0 = runif(2,
    -3.1, pi) kappa0 = runif(2, 0.1, 1.5) stepPar0 = c(mu0, sigma0) anglePar0 = c(mean0,
    kappa0) mods[[i]] <- fitHMM(highFreq, nbStates = 2, stepDist = "gamma", angleDist =
    "vm", stepPar0 = stepPar0, anglePar0 = anglePar0, stationary = T, verbose = 0)
    llks[i] <- -mods[[i]]$mod$minimum }
plot(1:nb.fit, llks, pch = 16)
```

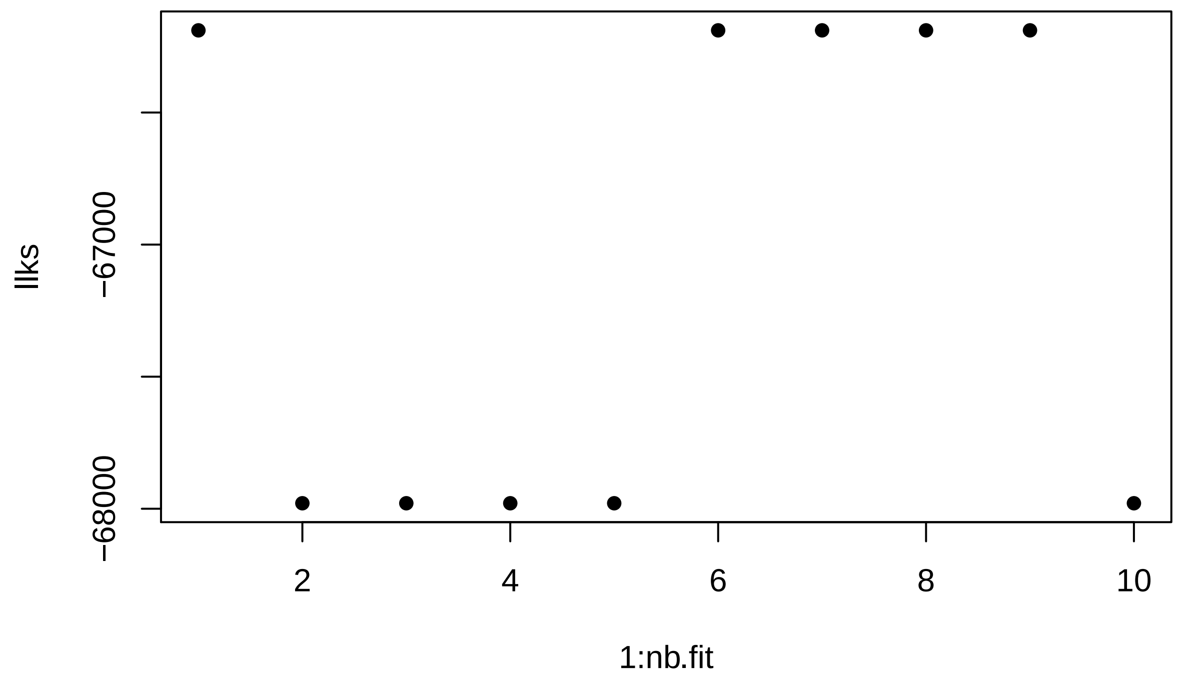


```
mods[[which.max(llks)]]$mod$minimum
```

```
## [1] 66188.86
```

```
mod0$mod$minimum
```

```
## [1] 66188.86
```

```
### state decoding
highFreq$states2 <- viterbi(mod0)


### 3-state HMM initial parameter values
mu0 = c(11, 40, 52)
sigma0 = c(8, 30, 36)
pi0 <- c(-3.1, 0, 3.1)
kappa0 = c(0.55, 0.4, 12)
stepPar0 = c(mu0, sigma0)
anglePar0 = c(pi0, kappa0)

mod <- fitHMM(highFreq, nbStates = 3, stepDist = "gamma", angleDist = "vm", stepPar0 =
    stepPar0, anglePar0 = anglePar0, stationary = T, verbose = 0)
mod
```

```
## Value of the maximum log-likelihood: -65522
```

```
##
## Step length parameters:
## ----------------------
##       state 1  state 2  state 3
## mean 11.24179 40.01147 51.71163
## sd    7.87601 29.37282 35.96386
##
## Turning angle parameters:
## ------------------------
##                  state 1     state 2  state 3
## mean          -3.1370479 -0.06405651  3.13006
## concentration  0.5551247  0.40451563 11.86077
##
## Regression coeffs for the transition probabilities:
## --------------------------------------------------
##              1 -> 2    1 -> 3    2 -> 1     2 -> 3    3 -> 1    3 -> 2
## intercept -2.273015 -6.790311 -2.201311 -0.2468513 0.3322202 0.2246709
##
## Transition probability matrix:
## -----------------------------
##            [,1]       [,2]       [,3]
## [1,] 0.90569395 0.09328749 0.00101856
## [2,] 0.05848995 0.52856502 0.41294503
## [3,] 0.38235631 0.34336829 0.27427540
##
## Initial distribution:
## --------------------
## [1] 0.6517375 0.2213801 0.1268824
```

```r
plot(mod, animals = 1)
```

```
## Decoding states sequence... DONE
```

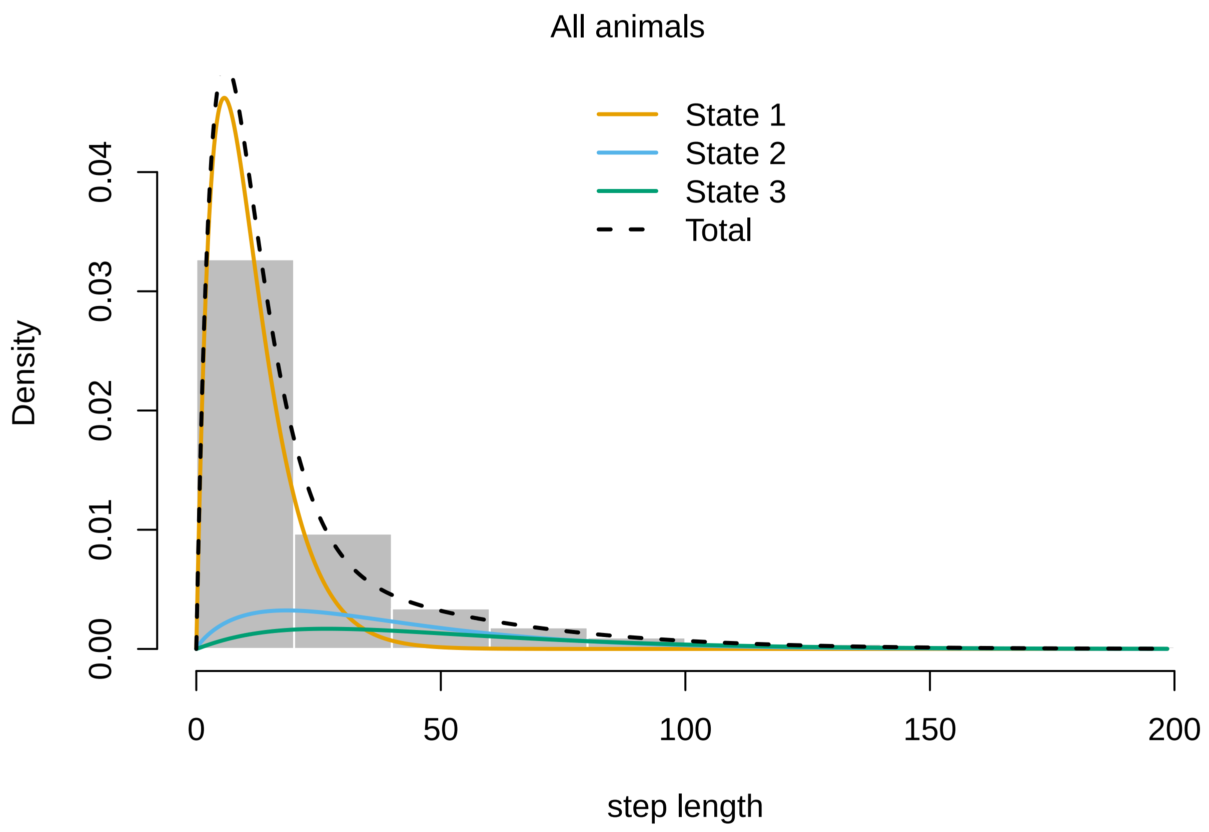
All animals
State 1
State 2
State 3
Total
Density
0.00
0.01
0.02
0.03
0.04
0
50
100
150
200
step length

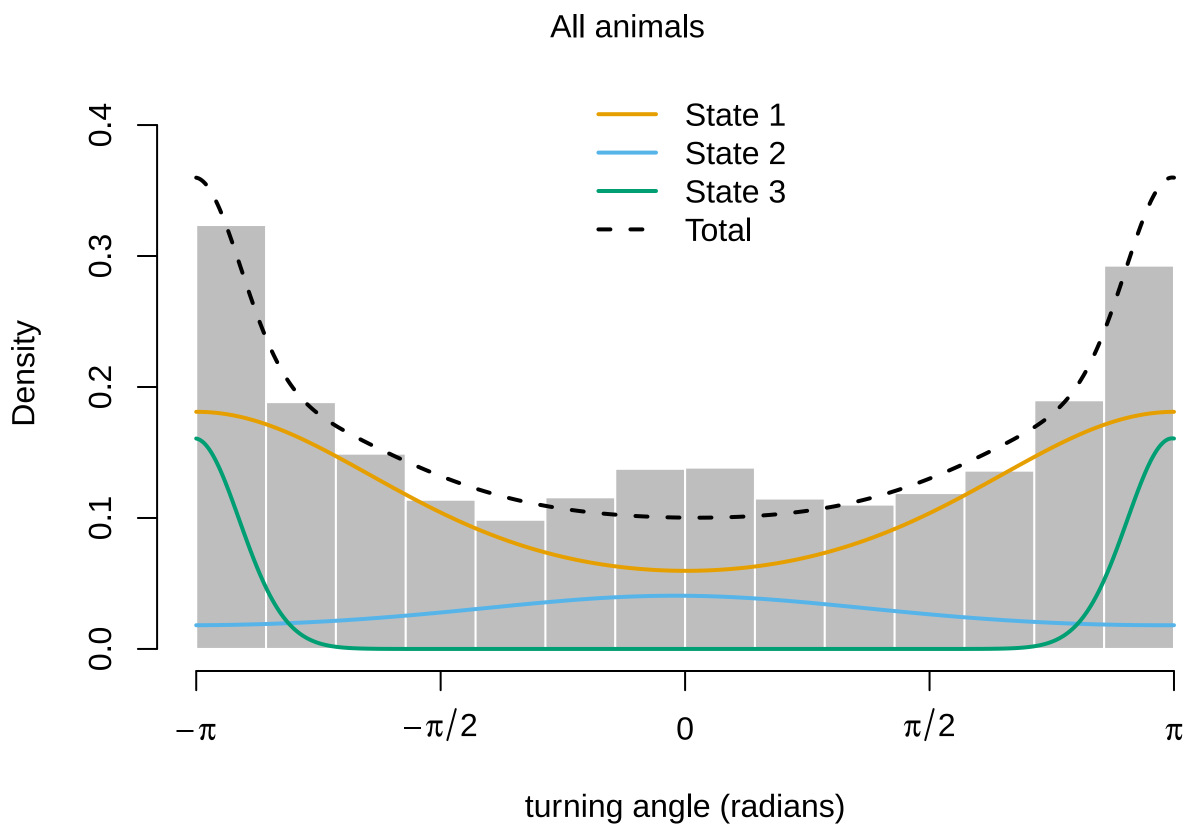
All animals
State 1
State 2
State 3
Total
Density
0.0
0.1
0.2
0.3
0.4
−π
−π/2
0
π/2
π
turning angle (radians)

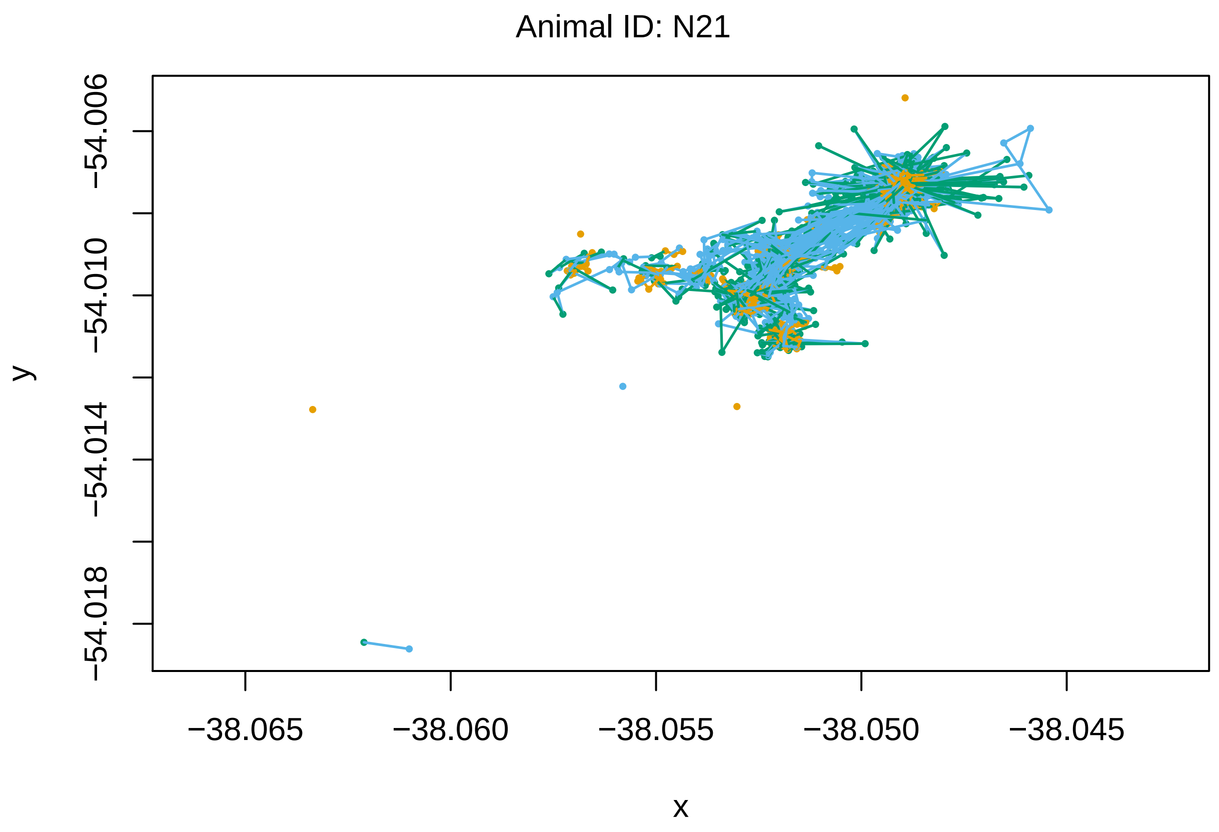


```r
# model checking using pseudo-residuals
plotPR(mod)
```

```
## Computing pseudo-residuals... DONE
```

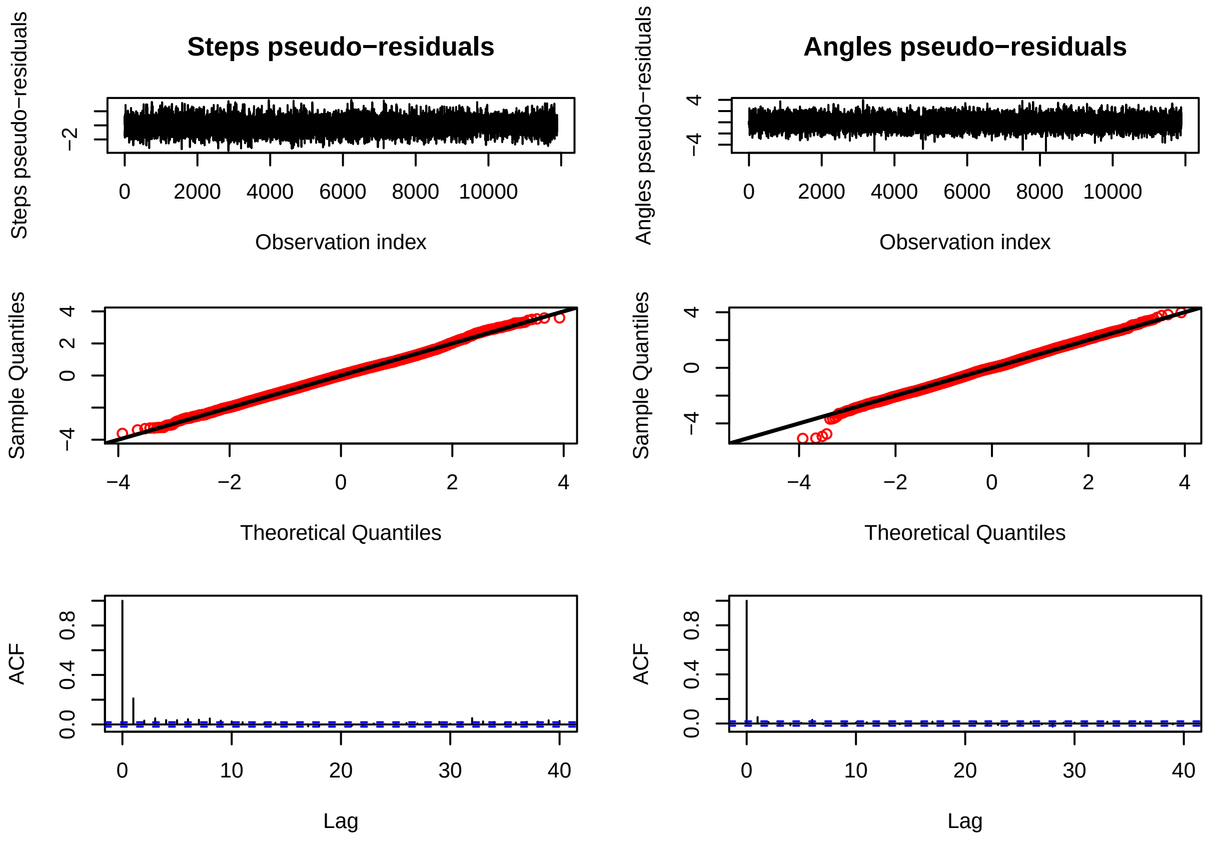


```
## code to fit several (3-state) HMMs using random starting values (to check for
## local maxima)
nb.fit <- 10 # number of models to fit
llks <- numeric(nb.fit)
mods <- list()
for (i in 1:nb.fit) { mu0 = runif(3, 5, 100) sigma0 = runif(3, 5, 50) mean0 = runif(3,
    -3.1, pi) kappa0 = runif(3, 0.1, 1.5) stepPar0 = c(mu0, sigma0) anglePar0 = c(mean0,
    kappa0) mods[[i]] <- fitHMM(highFreq, nbStates = 3, stepDist = "gamma", angleDist =
    "vm", stepPar0 = stepPar0, anglePar0 = anglePar0, stationary = T, verbose = 0)
    llks[i] <- -mods[[i]]$mod$minimum }
plot(1:nb.fit, llks, pch = 16)
```

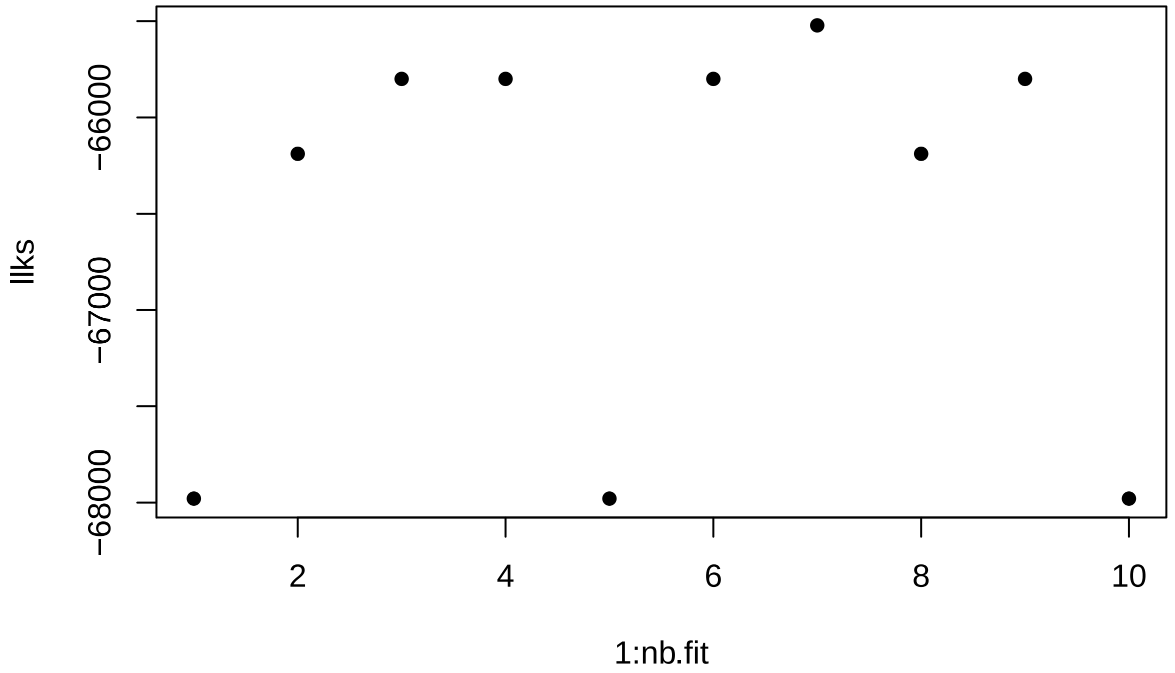


```
mods[[which.max(llks)]]$mod$minimum
```

```
## [1] 65522
```

```
mod$mod$minimum
```

```
## [1] 65522
```

```
### state decoding
highFreq$states <- viterbi(mod)
```

Hourly GPS data

Hourly GPS data were collected from 66 fur seal pups from two adjacent breeding colonies. Pups tracked from birth until moulting in order to investigate the factors shaping movement patterns and activity levels. Deployment durations ranged from 20 to 80 days (median = 51 days).

```
### hourly GPS data
allData <- read.csv("allData.csv")
```

```r
### data preparation
allData <- prepData(allData, coordNames = c("Longitude", "Latitude"))
allData$step <- allData$step * 1000
head(allData)
```

```
##     ID       step      angle         x         y IDno BurstNo
## 1 C1.1 130.047169         NA -38.05123 -54.00792   C1       1
## 2 C1.1   3.473101  2.3923474 -38.05101 -54.00908   C1       1
## 3 C1.1   3.897306  0.6701058 -38.05098 -54.00906   C1       1
## 4 C1.1   7.751492 -2.0447505 -38.05098 -54.00902   C1       1
## 5 C1.1  11.125718  3.0042979 -38.05088 -54.00905   C1       1
## 6 C1.1  10.273673 -3.0484593 -38.05102 -54.00900   C1       1
##              DateTime Hour WIND_SPEED DRYBULB_TEMPERATURE Beach Weight
## 1 2018-12-10 16:09:48   16          4                 2.8   FWB    6.6
## 2 2018-12-10 17:10:03   17          5                 2.9   FWB    6.6
## 3 2018-12-10 18:10:14   18          4                 3.0   FWB    6.6
## 4 2018-12-10 19:10:48   19          4                 2.7   FWB    6.6
## 5 2018-12-10 20:10:52   20          3                 2.4   FWB    6.6
## 6 2018-12-10 21:11:30   21          4                 2.2   FWB    6.6
##   Aggressiveness LocalDensity Sex Death sunlight Age Weight_10Days       CI
## 1              1            7   M     N        Y   0           6.6 7.318679
## 2              1            7   M     N        Y   0           6.6 7.318679
## 3              1            7   M     N        Y   0           6.6 7.318679
## 4              1            7   M     N        Y   0           6.6 7.318679
## 5              1            7   M     N        Y   0           6.6 7.318679
## 6              1            7   M     N        Y   0           6.6 7.318679
##   Year
## 1    0
## 2    0
## 3    0
## 4    0
## 5    0
## 6    0
```

```r
x <- which(allData$step > 400)
allData$step[x] <- allData$angle[x] <- allData$x[x] <- allData$y[x] <- NA
allData$step[which(allData$step == 0)] <- NA

# recode binary variables
allData$Beach01 <- 0
allData$Beach01[which(allData$Beach == "FWB")] <- 1
allData$Sex01 <- 0
allData$Sex01[which(allData$Sex == "M")] <- 1
allData$Death01 <- 0
allData$Death01[which(allData$Death == "Y")] <- 1
allData$sunlight01 <- 0
allData$sunlight01[which(allData$sunlight == "N")] <- 1

# standardize metric variables
allData$Weight_sd <- scale(allData$Weight)
allData$WIND_SPEED_sd <- scale(allData$WIND_SPEED)
allData$DRYBULB_TEMPERATURE_sd <- scale(allData$DRYBULB_TEMPERATURE)
allData$Age_sd <- scale(allData$Age)
```

```
allData$CI_sd <- scale(allData$CI)

# get an overview of the variables step length & turning angle
par(mfrow = c(2, 1))
hist(allData$step, breaks = 60, col = "grey", xlab = "step length in metres", main =
    "Histogram", prob = T)
hist(allData$angle, breaks = 60, col = "grey", xlab = "turning angle in radians", main =
    "Histogram", prob = T)
```

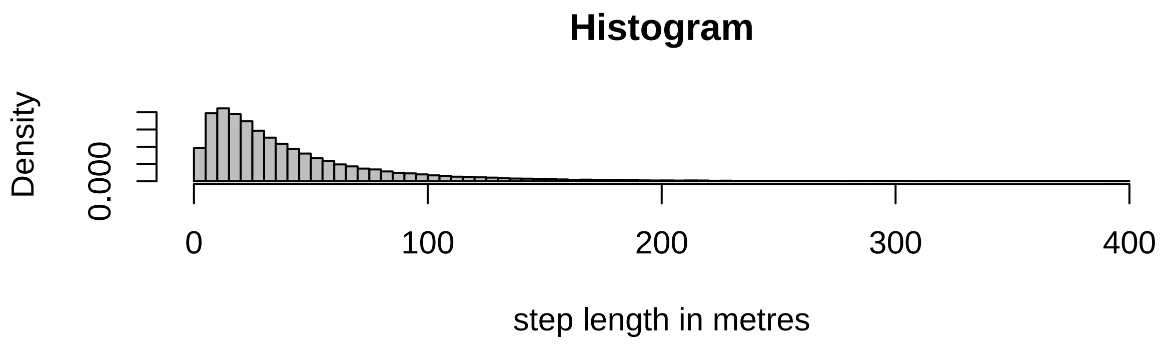

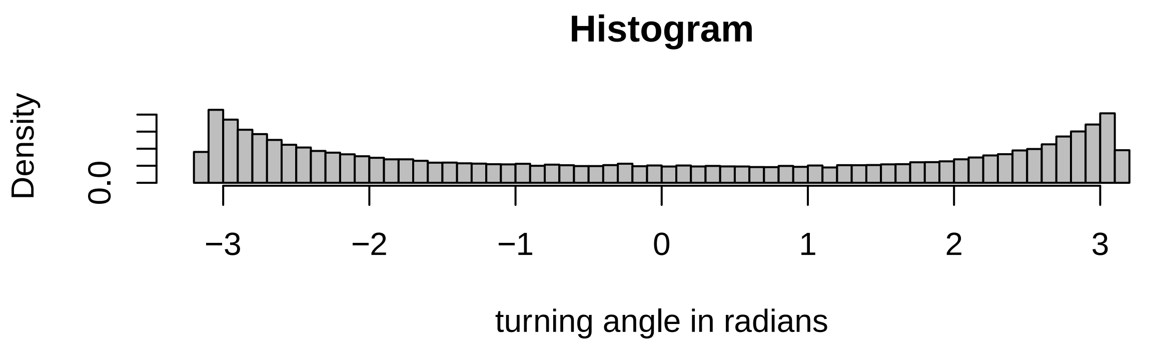


```
summary(allData$step)
```

```
##    Min. 1st Qu.  Median    Mean 3rd Qu.    Max.    NA's
##   0.111  14.895  29.232  43.859  55.415 399.491    4080
```

```
summary(allData$angle)
```

```
##    Min. 1st Qu.  Median    Mean 3rd Qu.    Max.    NA's
##  -3.142  -2.272  -0.210  -0.078   2.173   3.142    5778
```

Determine the number of HMM states - 2 vs 3 state HMMs without covariates

As with the high frequency (5-minute interval GPS data), 2 and 3 state HMMs without covariates were fitted to the hourly data. Only models with a biologically meaningful numbers of states should be tested. The number of states in the final HMM is determined by model fit.

```r
### HMMs

## 2-state HMM without covariates initial parameter values
mu0 = c(20, 90)
sigma0 = c(10, 70)
kappa0 = c(0.5, 1)
stepPar0 = c(mu0, sigma0)
anglePar0 = c(-3, -3.1, kappa0)

mod0 <- fitHMM(allData, nbStates = 2, stepDist = "gamma", angleDist = "vm", stepPar0 =
    stepPar0, anglePar0 = anglePar0, stationary = T, verbose = 0)
mod0
```

```
## Value of the maximum log-likelihood: -403403.2
##
## Step length parameters:
## ----------------------
##       state 1  state 2
## mean 22.31599 77.36962
## sd   15.73308 52.15421
##
## Turning angle parameters:
## ------------------------
##                  state 1    state 2
## mean          -2.9999500 -3.0940383
## concentration  0.5077446  0.8983488
##
## Regression coeffs for the transition probabilities:
## --------------------------------------------------
##             1 -> 2   2 -> 1
## intercept -1.67724 -1.14185
##
## Transition probability matrix:
## -----------------------------
##           [,1]      [,2]
## [1,] 0.8425388 0.1574612
## [2,] 0.2419808 0.7580192
##
## Initial distribution:
## --------------------
## [1] 0.605797 0.394203
```

```r
plot(mod0, animals = 1)
```

```
## Decoding states sequence... DONE
```

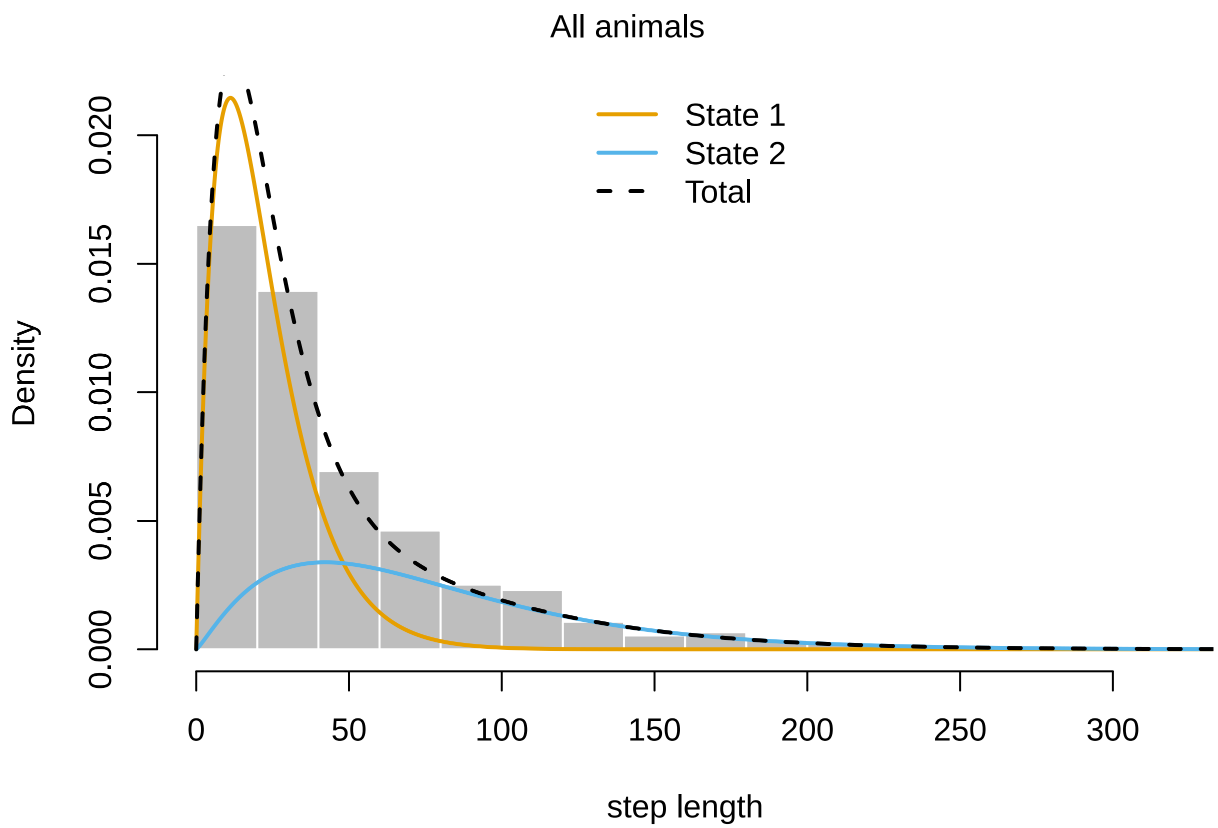
All animals
State 1
State 2
Total
Density
0.000
0.005
0.010
0.015
0.020
0
50
100
150
200
250
300
step length

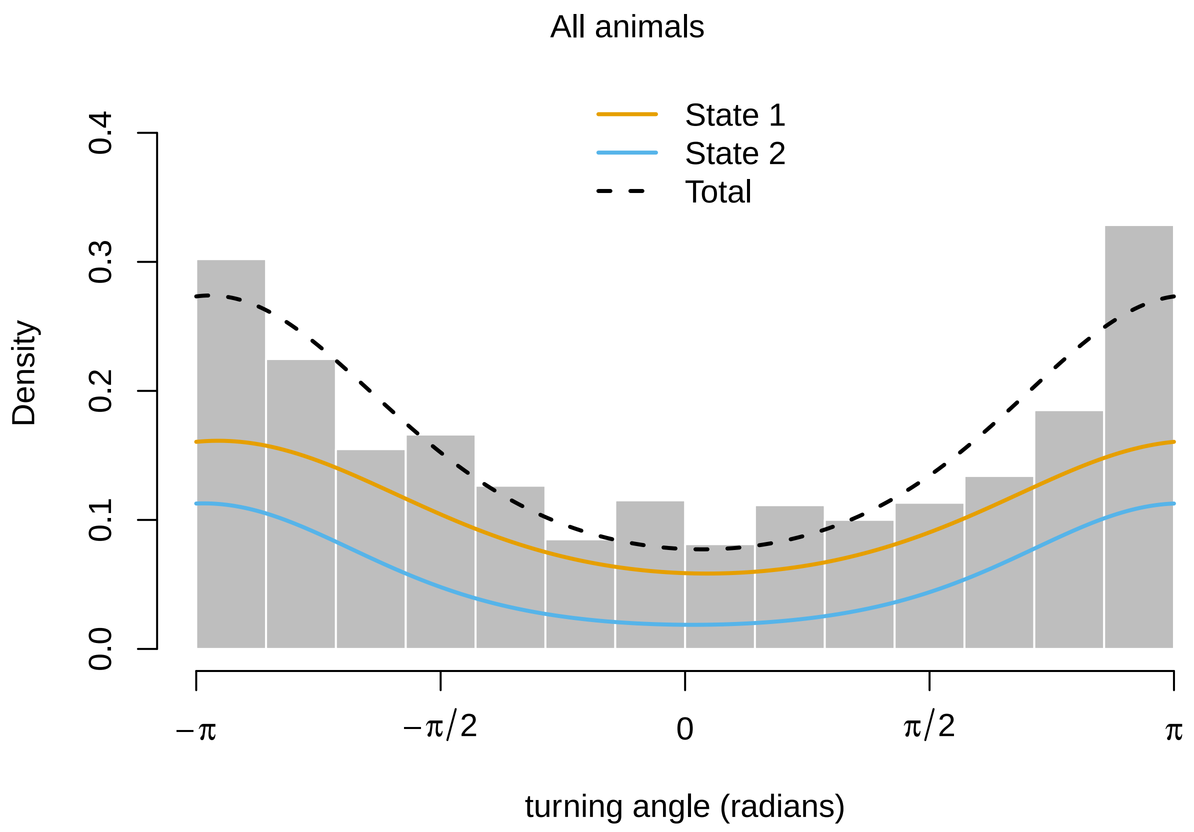
All animals
State 1
State 2
Total
Density
0.0
0.1
0.2
0.3
0.4
−π
−π/2
0
π/2
π
turning angle (radians)

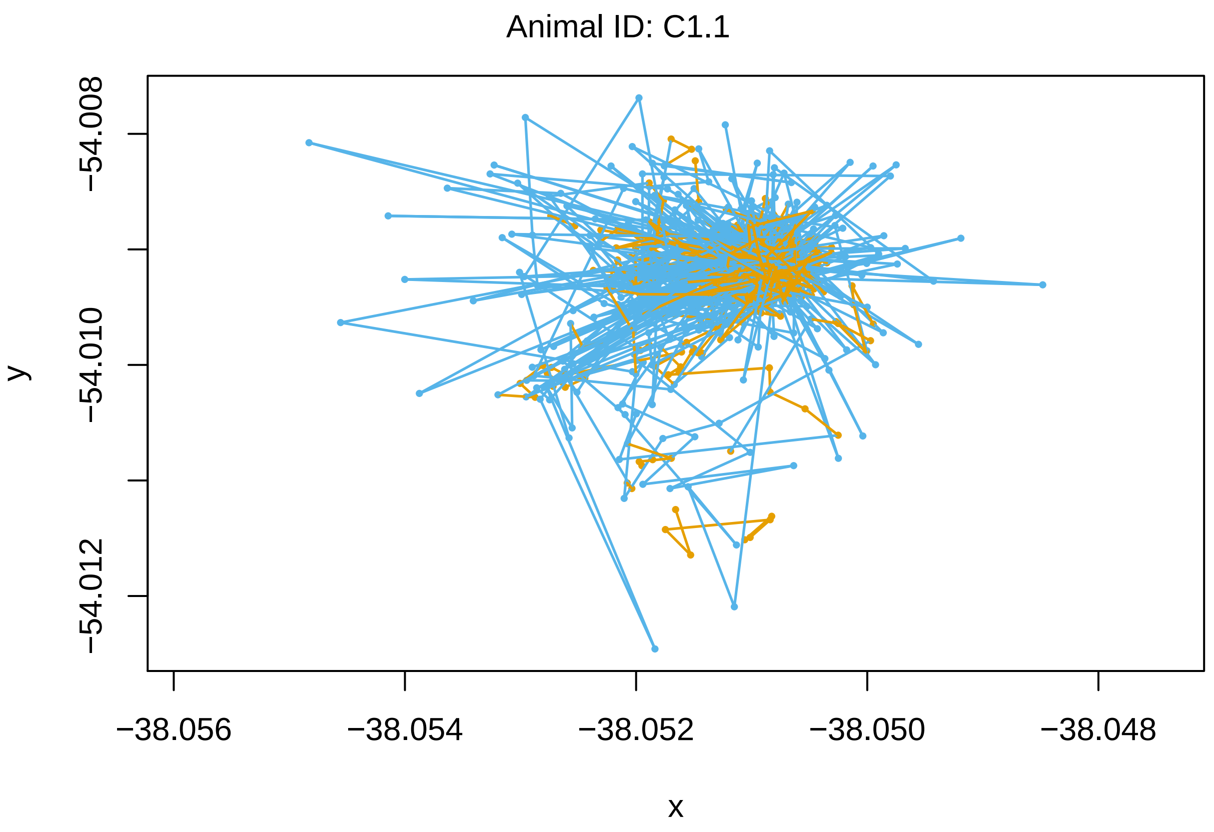


```r
# model checking using pseudo-residuals
plotPR(mod0)
```

```
## Computing pseudo-residuals... DONE
```

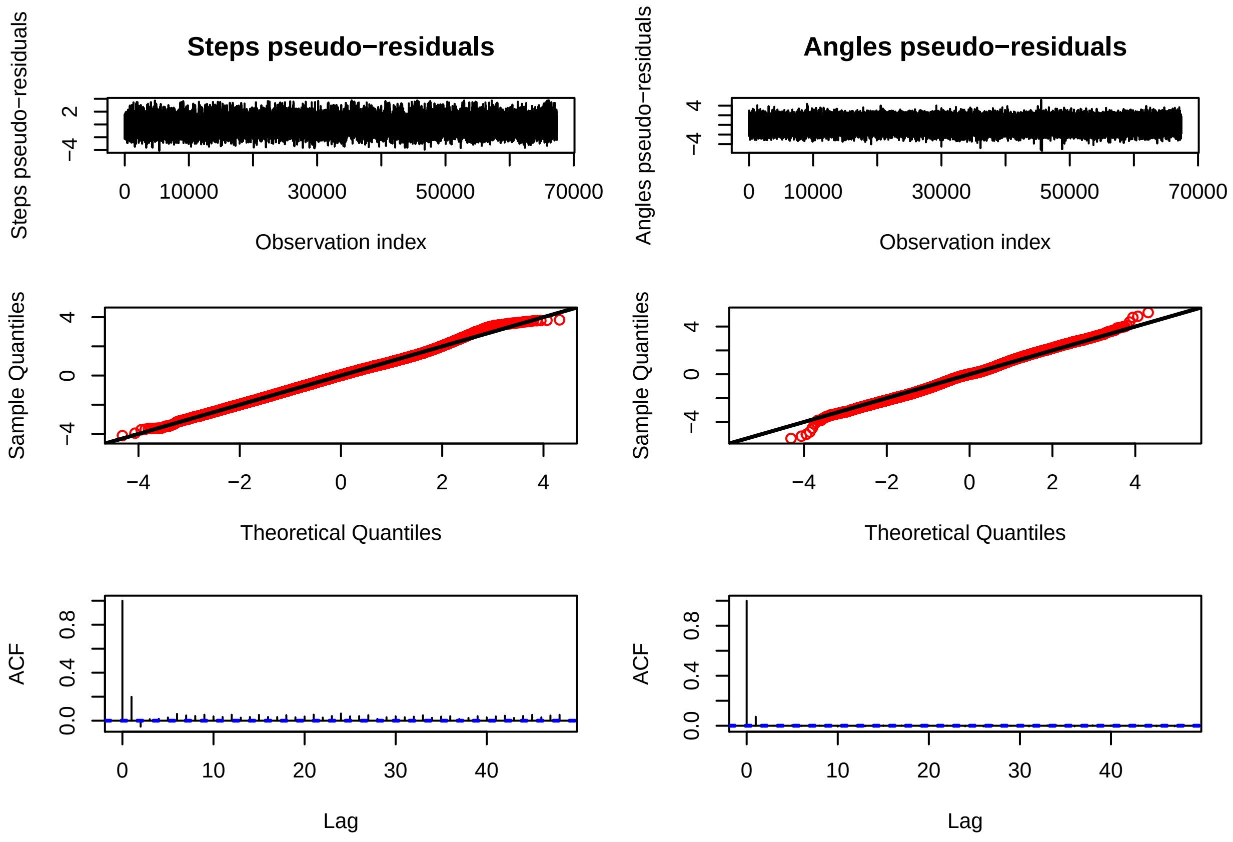


```r
## code to fit several (2-state) HMMs using random starting values (to check for
## local maxima)
nb.fit <- 10 # number of models to fit
llks <- numeric(nb.fit)
mods <- list()
for (i in 1:nb.fit) { mu0 = runif(2, 10, 150) sigma0 = runif(2, 5, 100) mean0 = runif(2,
    -3.1, pi) kappa0 = runif(2, 0.1, 1.5) stepPar0 = c(mu0, sigma0) anglePar0 = c(mean0,
    kappa0) mods[[i]] <- fitHMM(allData, nbStates = 2, stepDist = "gamma", angleDist =
    "vm", stepPar0 = stepPar0, anglePar0 = anglePar0, stationary = T, verbose = 0)
    llks[i] <- -mods[[i]]$mod$minimum }
plot(1:nb.fit, llks, pch = 16)
```

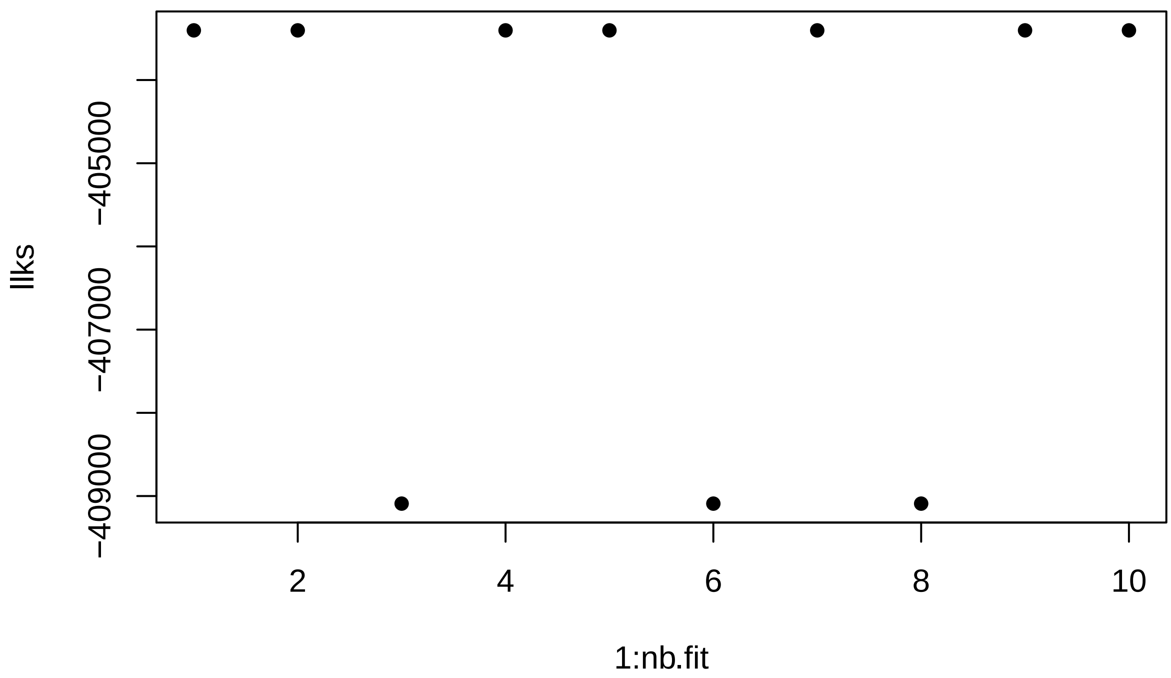


```
mods[[which.max(llks)]]$mod$minimum
```

```
## [1] 403403.2
```

```
mod0$mod$minimum
```

```
## [1] 403403.2
```

```
## 3-state HMM without covariates initial parameter values
mu0 = c(20, 55, 150)
sigma0 = c(10, 25, 90)
mean0 = c(-3, -3, 0)
kappa0 = c(0.4, 1, 1)
stepPar0 = c(mu0, sigma0)
anglePar0 = c(mean0, kappa0)

mod3N <- fitHMM(allData, nbStates = 3, stepDist = "gamma", angleDist = "vm", stepPar0 =
    stepPar0, anglePar0 = anglePar0, stationary = T, verbose = 0)
mod3N
```

```
## Value of the maximum log-likelihood: -401461.9
##
## Step length parameters:
## ----------------------
```

```
##       state 1  state 2   state 3
## mean 15.94608 44.81779 117.79036
## sd   10.51299 21.96331  63.33282
##
## Turning angle parameters:
## ------------------------
##                   state 1    state 2   state 3
## mean           -3.0022284 -3.0334230 3.1316302
## concentration  0.3693061  0.9199476 0.8313828
##
## Regression coeffs for the transition probabilities:
## ---------------------------------------------------
##              1 -> 2    1 -> 3    2 -> 1    2 -> 3    3 -> 1    3 -> 2
## intercept -1.201823 -2.316462 -1.133677 -2.115022 -1.121946 -1.038062
##
## Transition probability matrix:
## -----------------------------
##           [,1]      [,2]       [,3]
## [1,] 0.7146596 0.2148593 0.07048111
## [2,] 0.2231212 0.6932514 0.08362739
## [3,] 0.1938613 0.2108246 0.59531403
##
## Initial distribution:
## --------------------
## [1] 0.4296267 0.4106820 0.1596913
```

```
plot(mod3N, animals = 1)
```

```
## Decoding states sequence... DONE
```

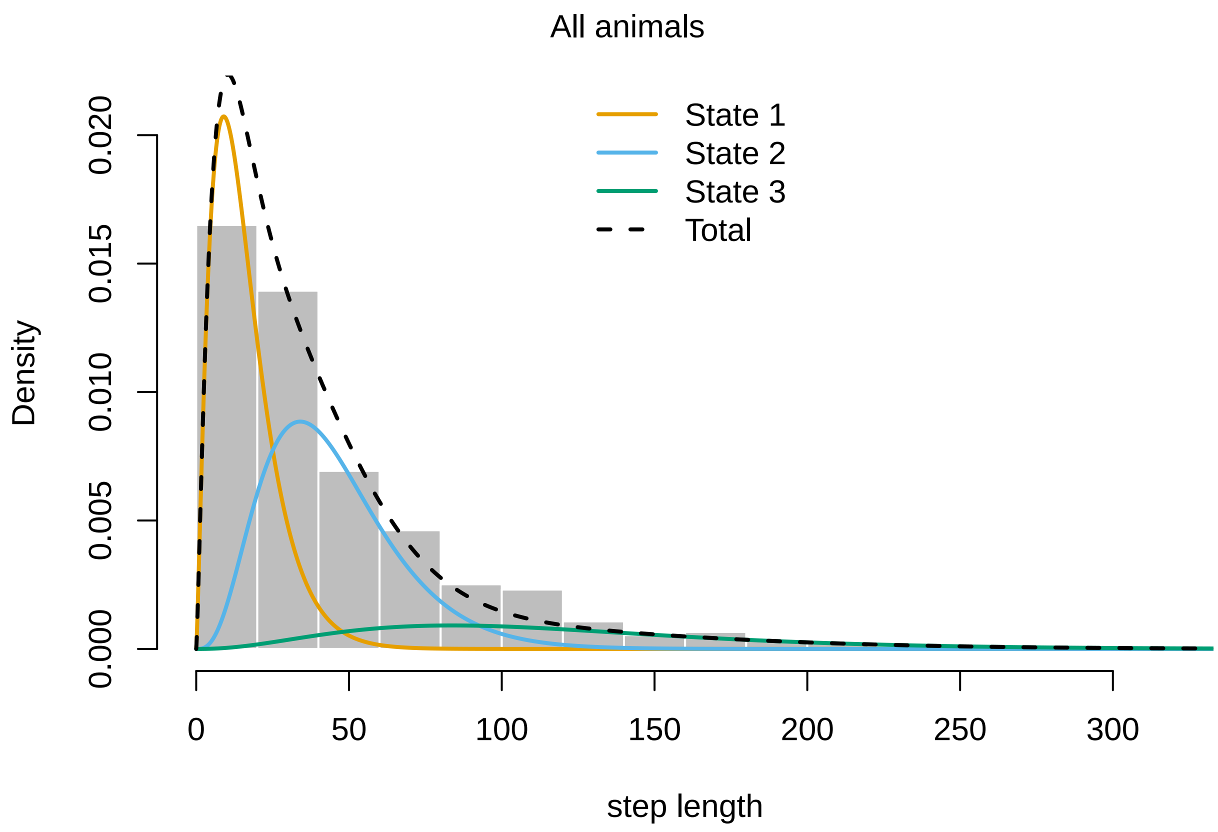
All animals
State 1
State 2
State 3
Total
Density
0.000
0.005
0.010
0.015
0.020
0
50
100
150
200
250
300
step length

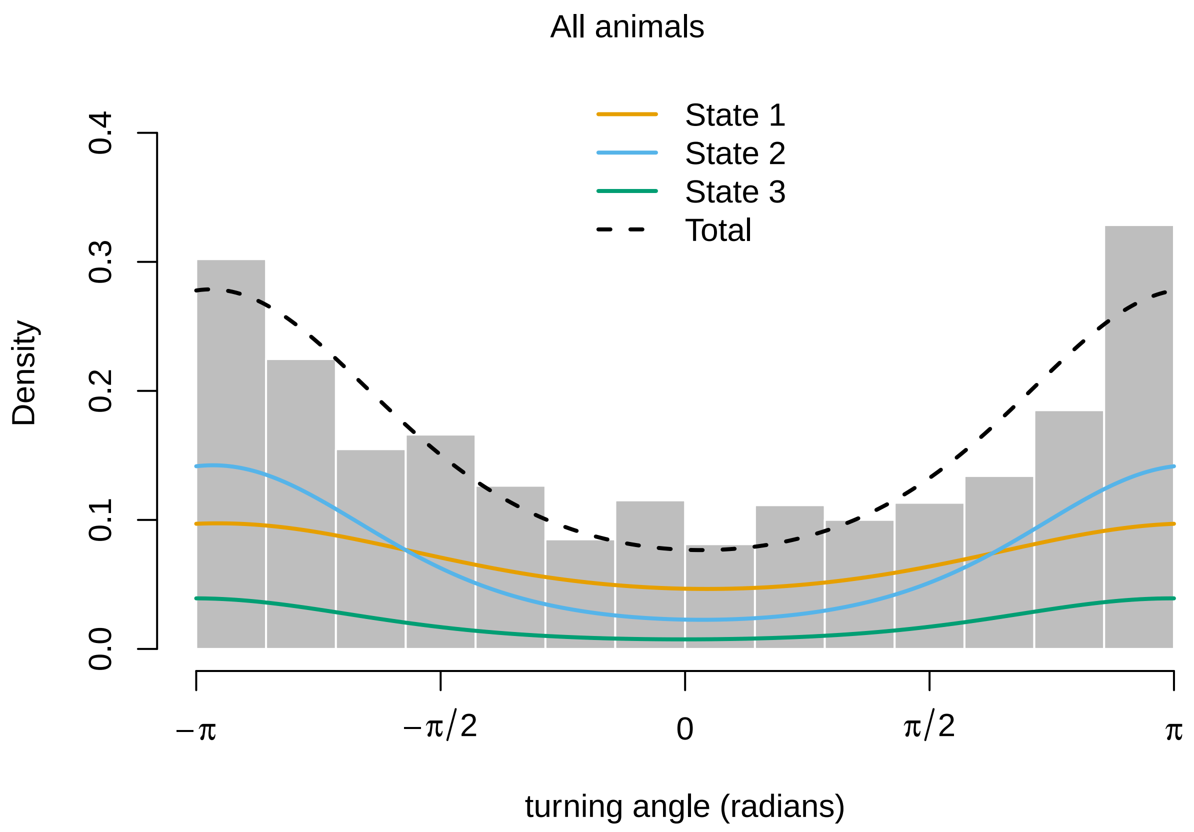
All animals
State 1
State 2
State 3
Total
Density
0.0
0.1
0.2
0.3
0.4
−π
−π/2
0
π/2
π
turning angle (radians)

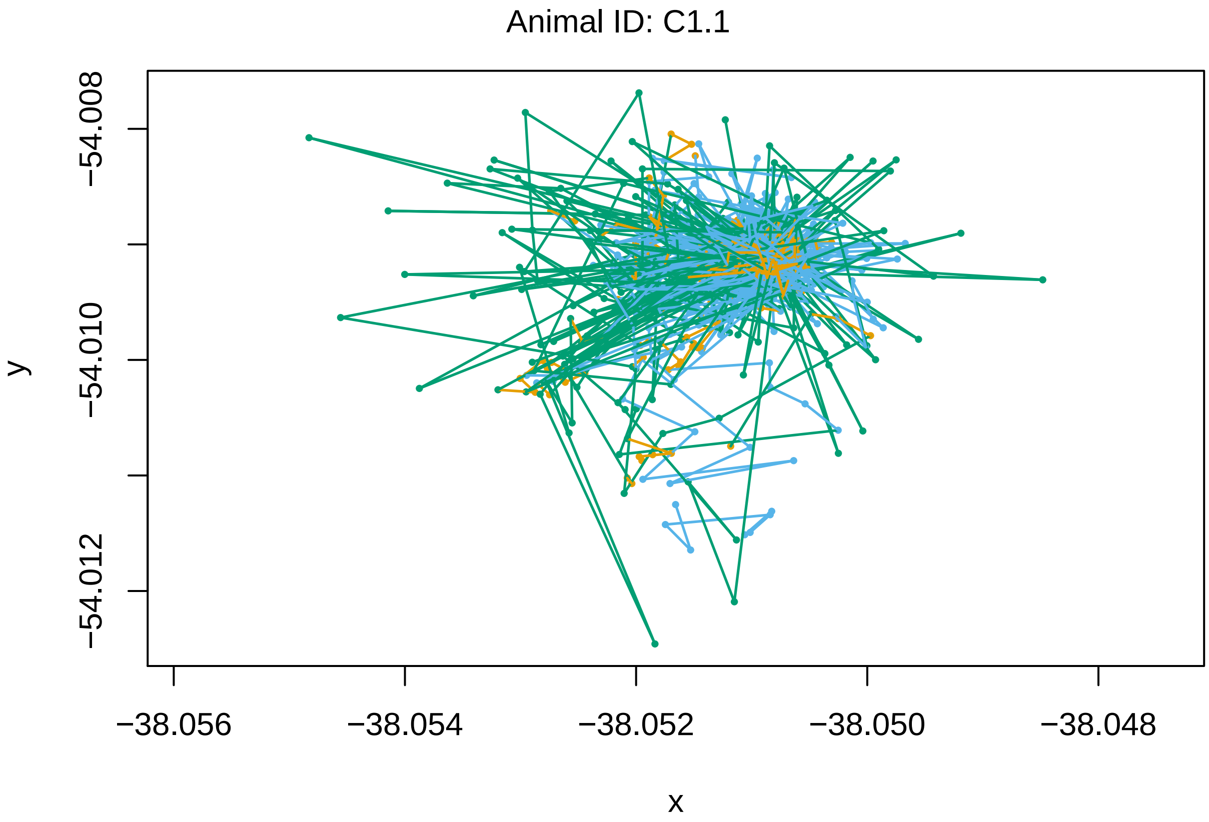


```r
# model checking using pseudo-residuals
plotPR(mod3N)
```

```
## Computing pseudo-residuals... DONE
```

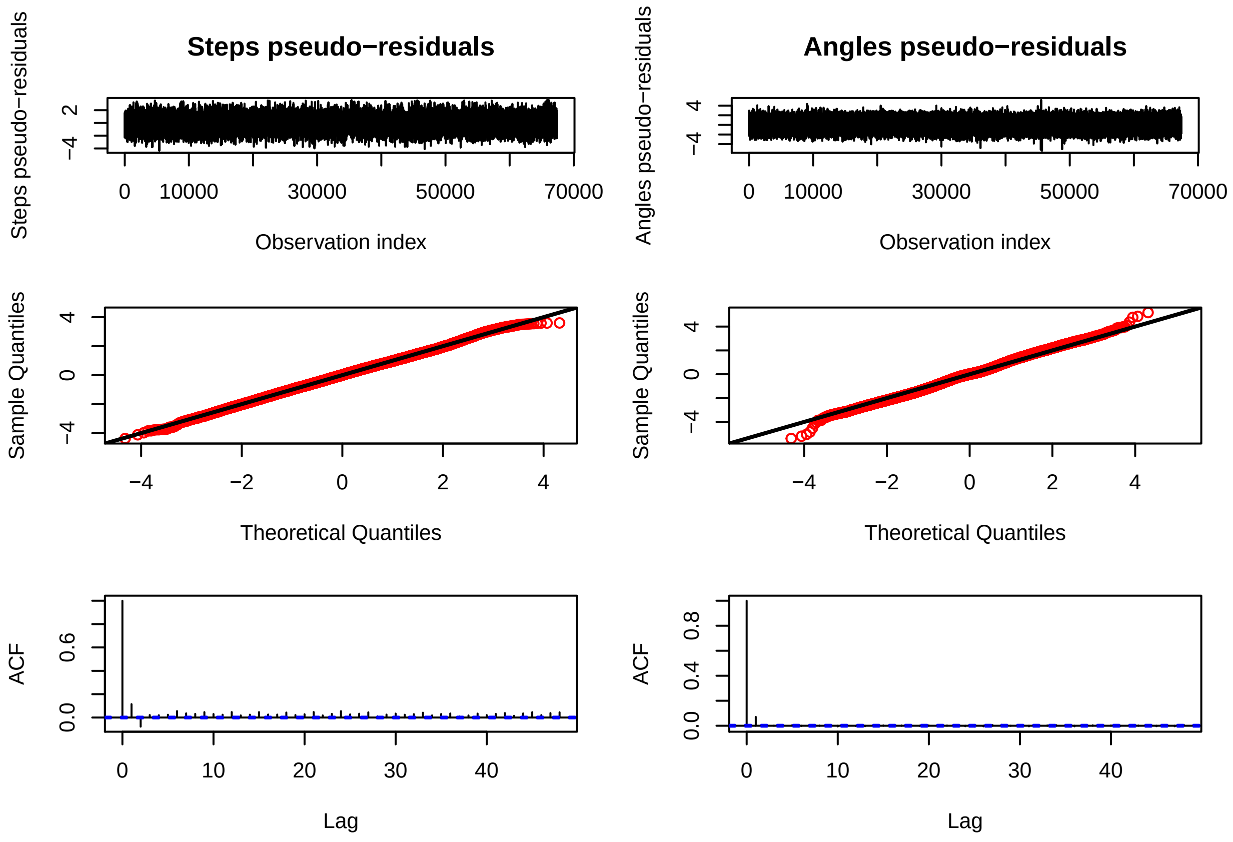

2 state HMM - include covariates

We restricted our final analysis to a parsimonious univariate HMM with 2 states. The inclusion of the turning angle as well as additional states resulted in a negligible improvement of model fit while complicating the interpretation of the underlying states.

Covariates included in the model were: Hourly dry bulb air temperature and wind speed measurements. Sex (with female as the reference category) and age (measured in days since initial capture, 2 – 3 days after birth) as well as the scaled mass index (CI), calculated every 10 days and kept constant between measurements. All metric variables were standardised. The variables season (with 2019 as the reference category) and time of day (measured in hours) were also included.

```r
## 2-state HMM including all covariates
mod_covsAll <- fitHMM(allData, nbStates = 2, stepDist = "gamma", angleDist = "vm",
    stepPar0 = as.vector(t(mod0$mle$stepPar)), anglePar0 =
    as.vector(t(mod0$mle$anglePar)), stationary = F, verbose = 0, formula = ~Sex01 +
    Age_sd + I(Age_sd^2) + CI_sd + sin((2 * pi * Hour)/24) + cos((2 * pi * Hour)/24) +
    Year + DRYBULB_TEMPERATURE_sd + WIND_SPEED_sd + Beach01 + Sex01:Beach01 +
    Age_sd:Beach01 + I(Age_sd^2):Beach01 + CI_sd:Beach01 + sin((2 * pi *
    Hour)/24):Beach01 + cos((2 * pi * Hour)/24):Beach01 + Year:Beach01 +
    DRYBULB_TEMPERATURE_sd:Beach01 + WIND_SPEED_sd:Beach01)

mod_covsAll
```

```
## Value of the maximum log-likelihood: -403120.2
```

```
##
## Step length parameters:
## ----------------------
##       state 1  state 2
## mean 22.21996 77.22047
## sd   15.65310 51.94968
##
## Turning angle parameters:
## ------------------------
##                  state 1    state 2
## mean          -2.9993286 -3.0940765
## concentration  0.5061349  0.8992607
##
## Regression coeffs for the transition probabilities:
## --------------------------------------------------
##                                    1 -> 2       2 -> 1
## intercept                     -1.68898437 -1.216600052
## Sex01                          0.14161237  0.046004775
## Age_sd                         0.04596180  0.062304608
## I(Age_sd^2)                   -0.03855691 -0.053828436
## CI_sd                          0.09051324 -0.018950563
## sin((2 * pi * Hour)/24)        0.01392432 -0.097398111
## cos((2 * pi * Hour)/24)       -0.22851210  0.051995386
## Year                           0.18315941  0.156607054
## DRYBULB_TEMPERATURE_sd        -0.09878877 -0.010003310
## WIND_SPEED_sd                 -0.03475351 -0.023588620
## Beach01                        0.35567354  0.280527711
## Sex01:Beach01                 -0.24098792 -0.158773662
## Age_sd:Beach01                 0.33882429 -0.144634986
## I(Age_sd^2):Beach01           -0.15595871 -0.005576965
## CI_sd:Beach01                  0.02002226  0.138712616
## sin((2 * pi * Hour)/24):Beach01 -0.07878720  0.063182054
## cos((2 * pi * Hour)/24):Beach01 -0.07886010  0.191124712
## Year:Beach01                  -0.57900434 -0.064147691
## DRYBULB_TEMPERATURE_sd:Beach01   0.11890812  0.032392193
## WIND_SPEED_sd:Beach01          0.01800277  0.051085646
##
## Initial distribution:
## --------------------
## [1] 0.7181295 0.2818705
```

```r
plot(mod_covsAll, animals = 1)
```

```
## Decoding states sequence... DONE
```

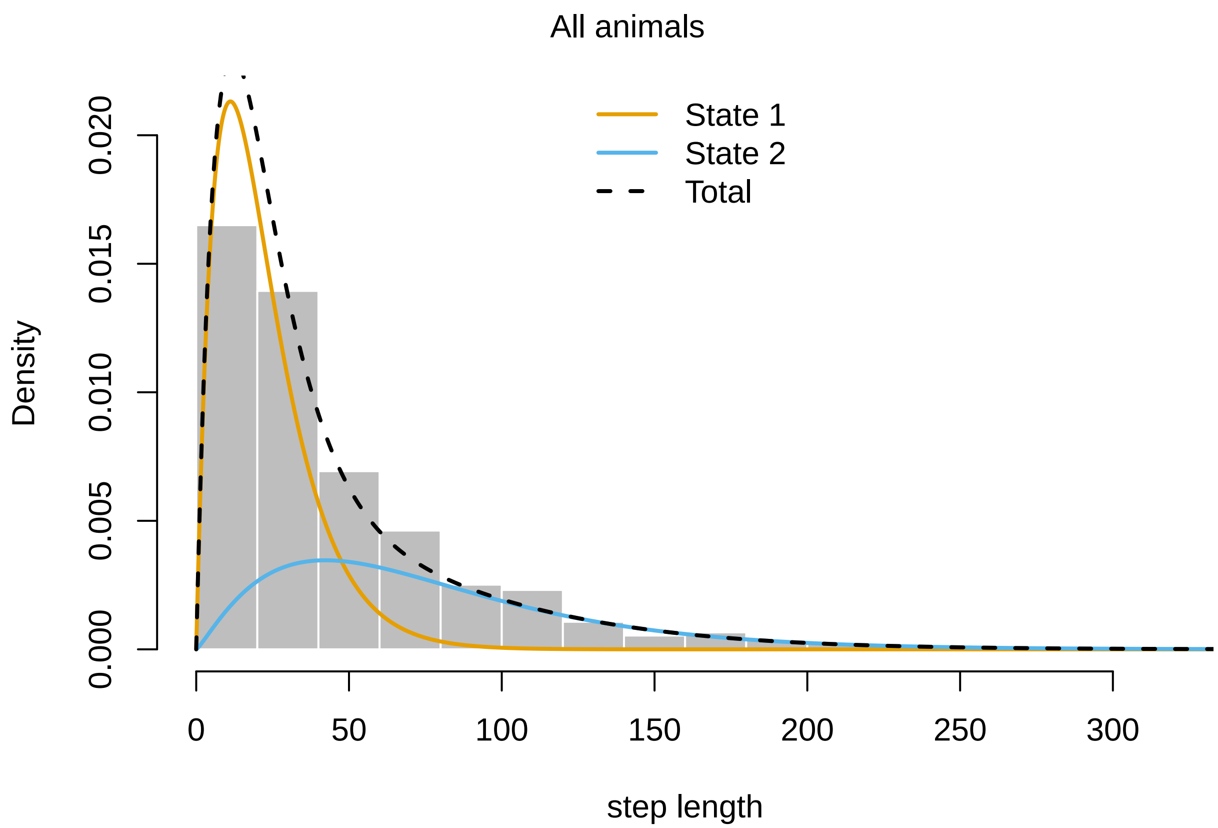

All animals
State 1
State 2
Total
Density
0.000
0.005
0.010
0.015
0.020
0
50
100
150
200
250
300
step length

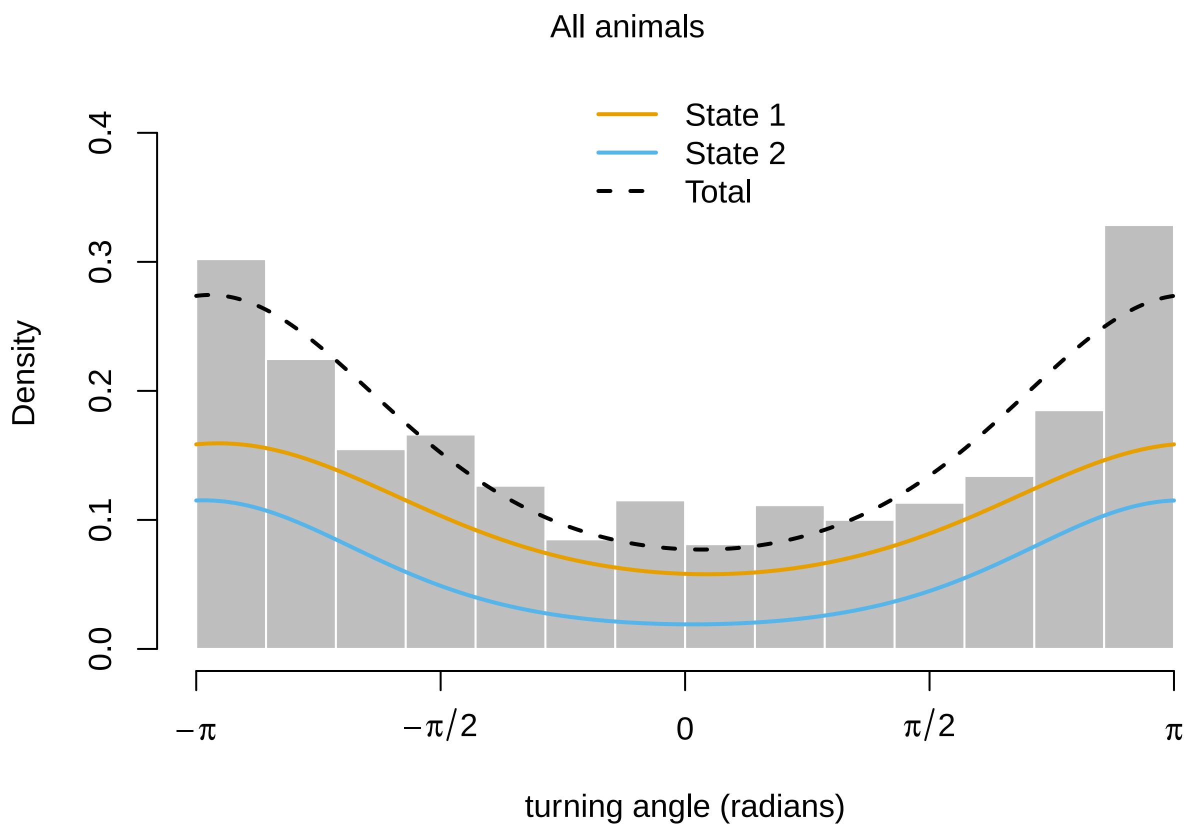
All animals
State 1
State 2
Total
Density
0.0
0.1
0.2
0.3
0.4
−π
−π/2
0
π/2
π
turning angle (radians)

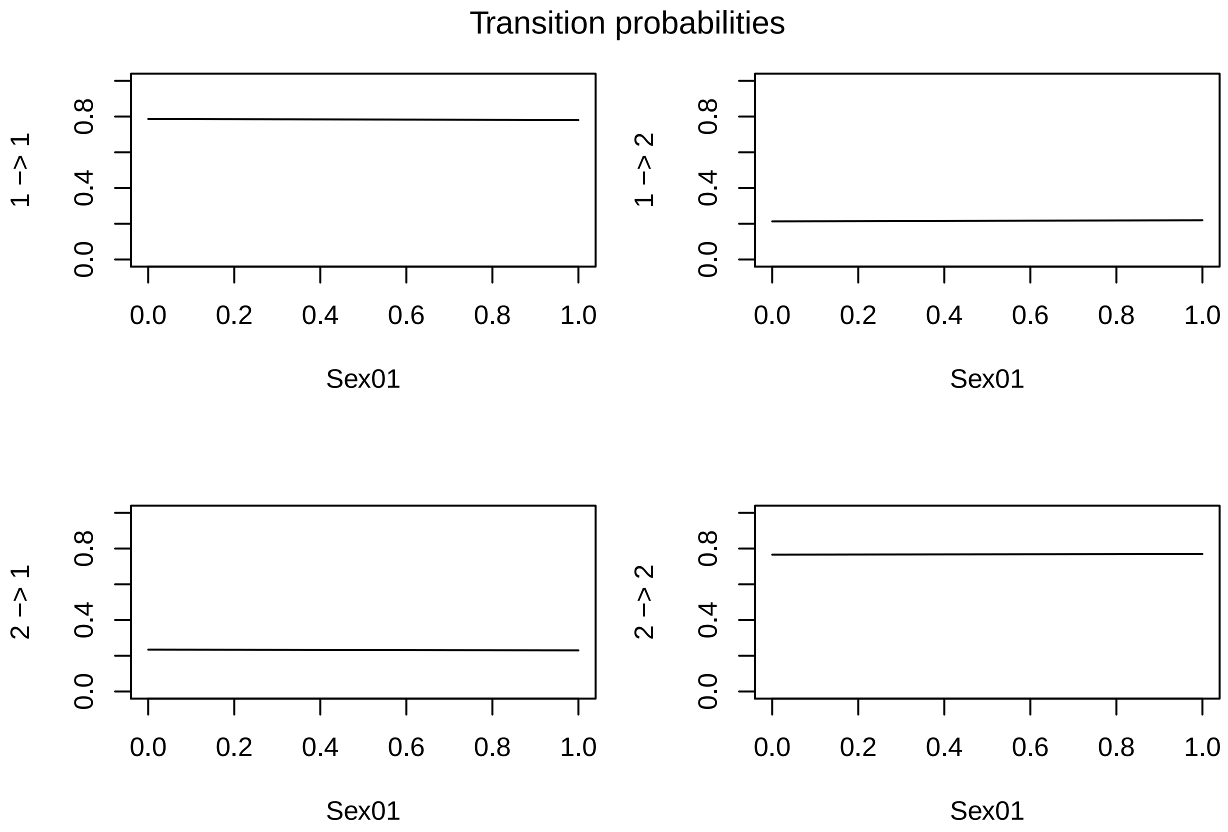
Transition probabilities
1 −> 1
1 −> 2
2 −> 1
2 −> 2
0.0
0.4
0.8
0.0
0.2
0.4
0.6
0.8
1.0
Sex01

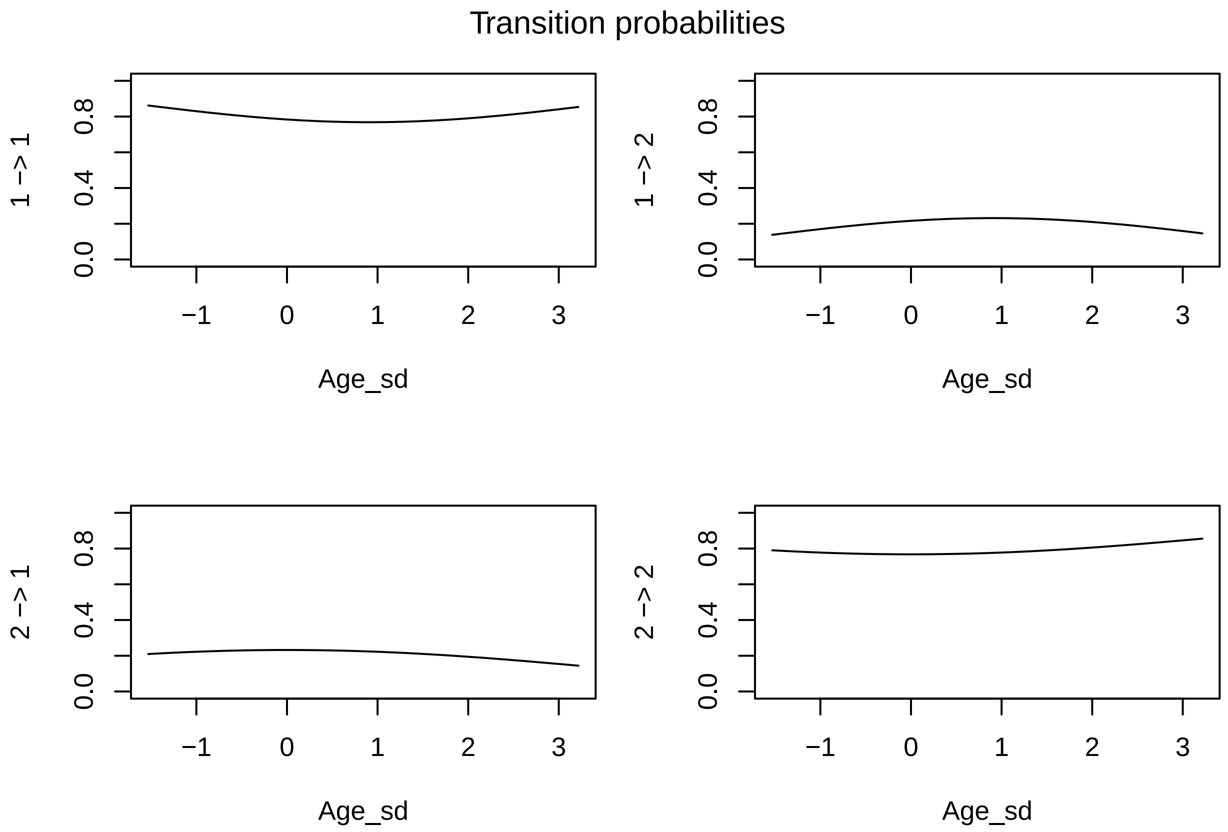

Transition probabilities
1 −> 1
1 −> 2
2 −> 1
2 −> 2
0.0
0.4
0.8
−1
0
1
2
3
Age_sd

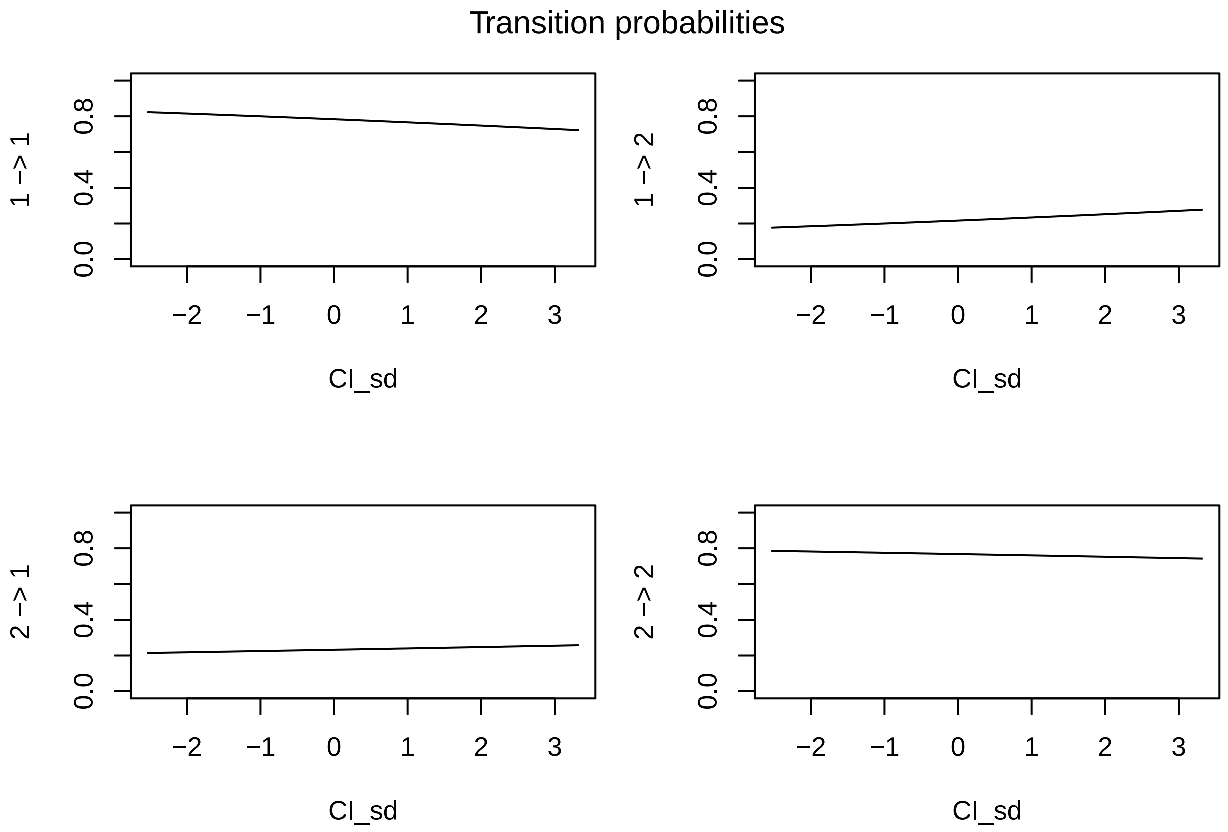
Transition probabilities
1 -> 1
1 -> 2
2 -> 1
2 -> 2
0.0
0.4
0.8
−2
−1
0
1
2
3
CI_sd

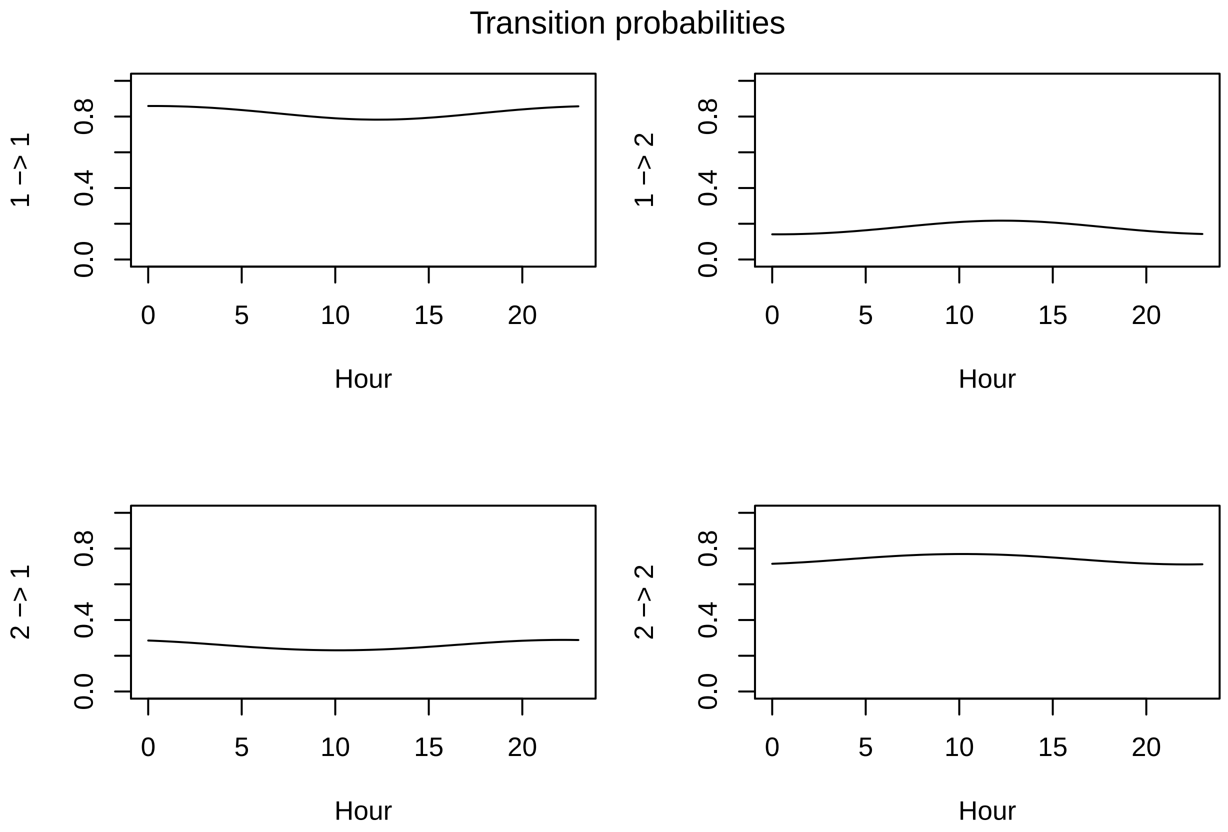

Transition probabilities
1 -> 1
1 -> 2
2 -> 1
2 -> 2
0.0
0.4
0.8
0
5
10
15
20
Hour

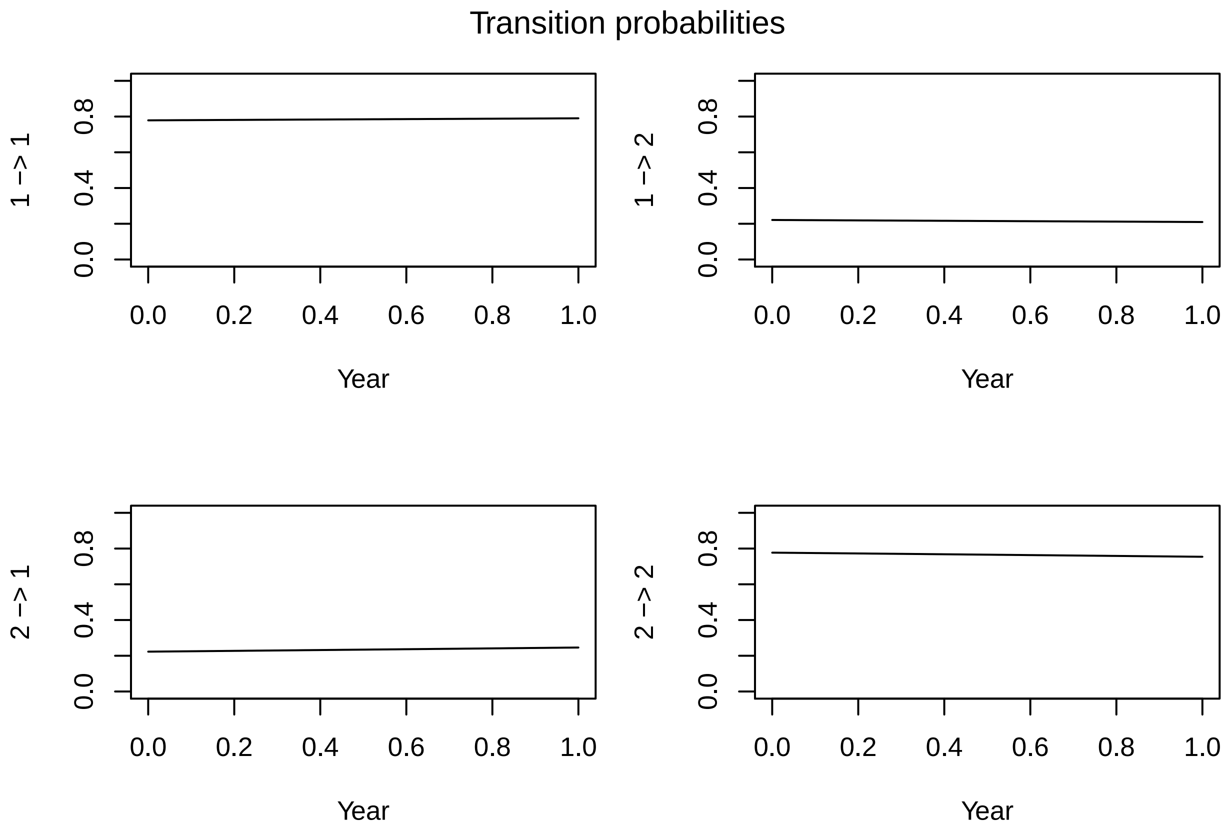
Transition probabilities
1 −> 1
1 −> 2
2 −> 1
2 −> 2
0.0
0.4
0.8
0.0
0.2
0.4
0.6
0.8
1.0
Year

Transition probabilities

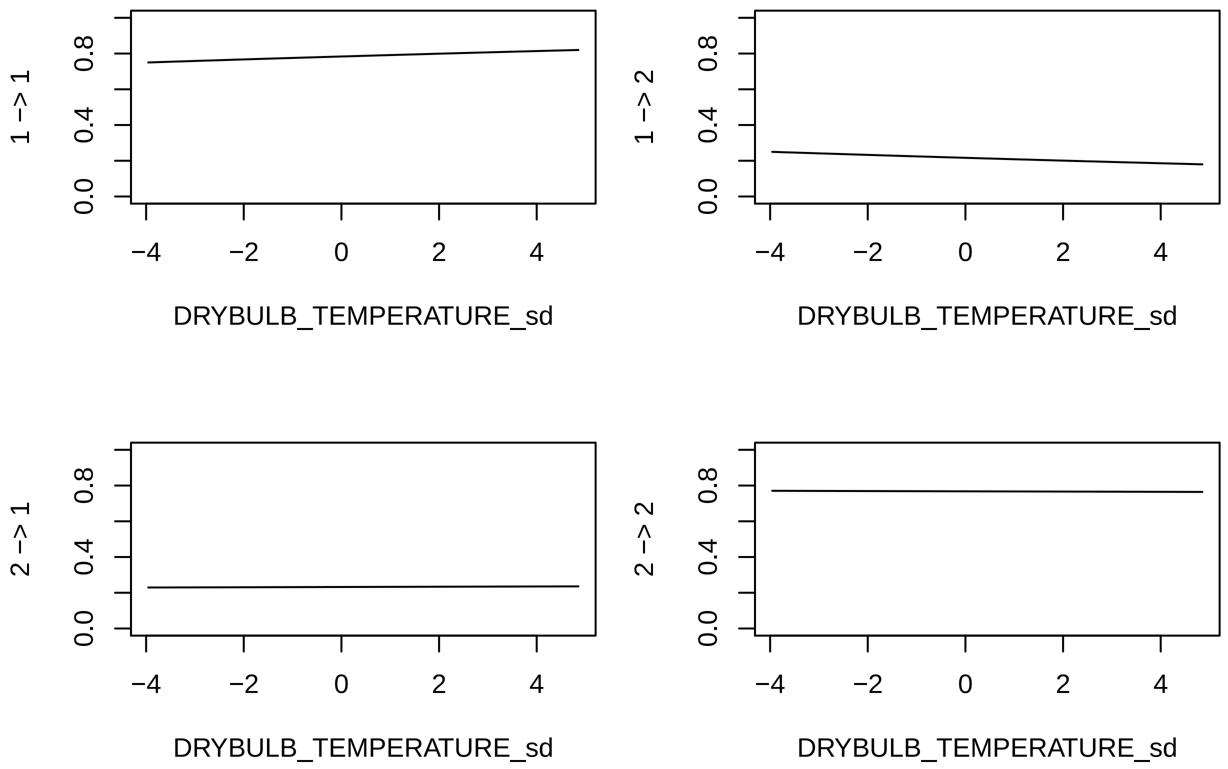

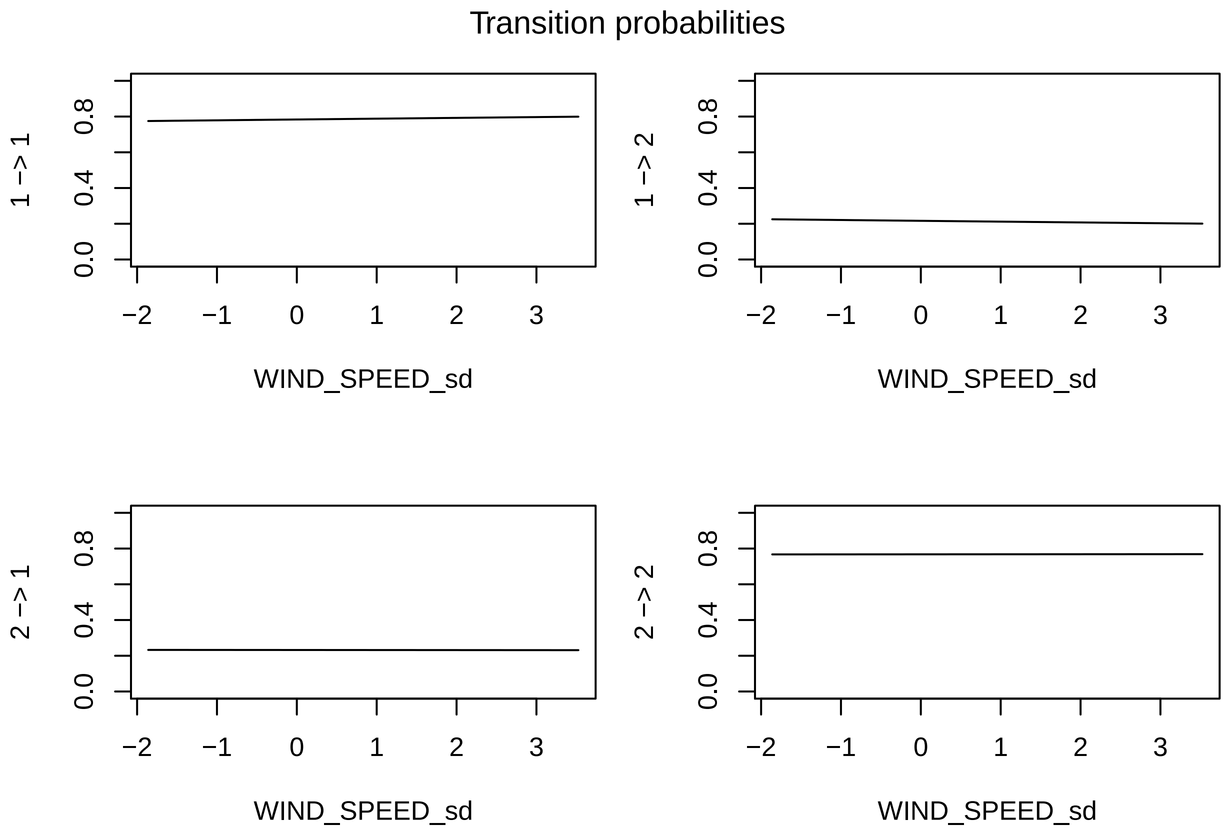
Transition probabilities
1 −> 1
1 −> 2
2 −> 1
2 −> 2
0.0
0.4
0.8
−2
−1
0
1
2
3
WIND_SPEED_sd

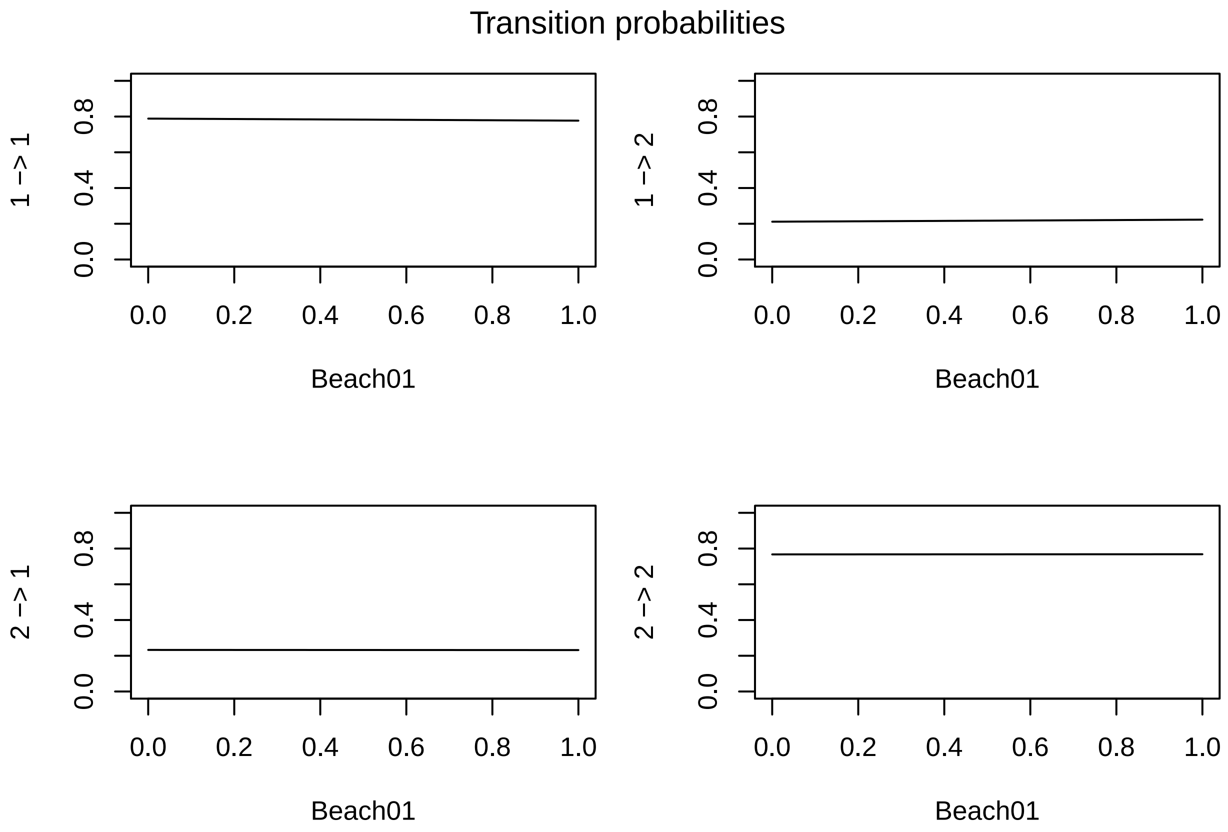
Transition probabilities
1 −> 1
1 −> 2
2 −> 1
2 −> 2
0.0
0.4
0.8
0.0
0.2
0.4
0.6
0.8
1.0
Beach01

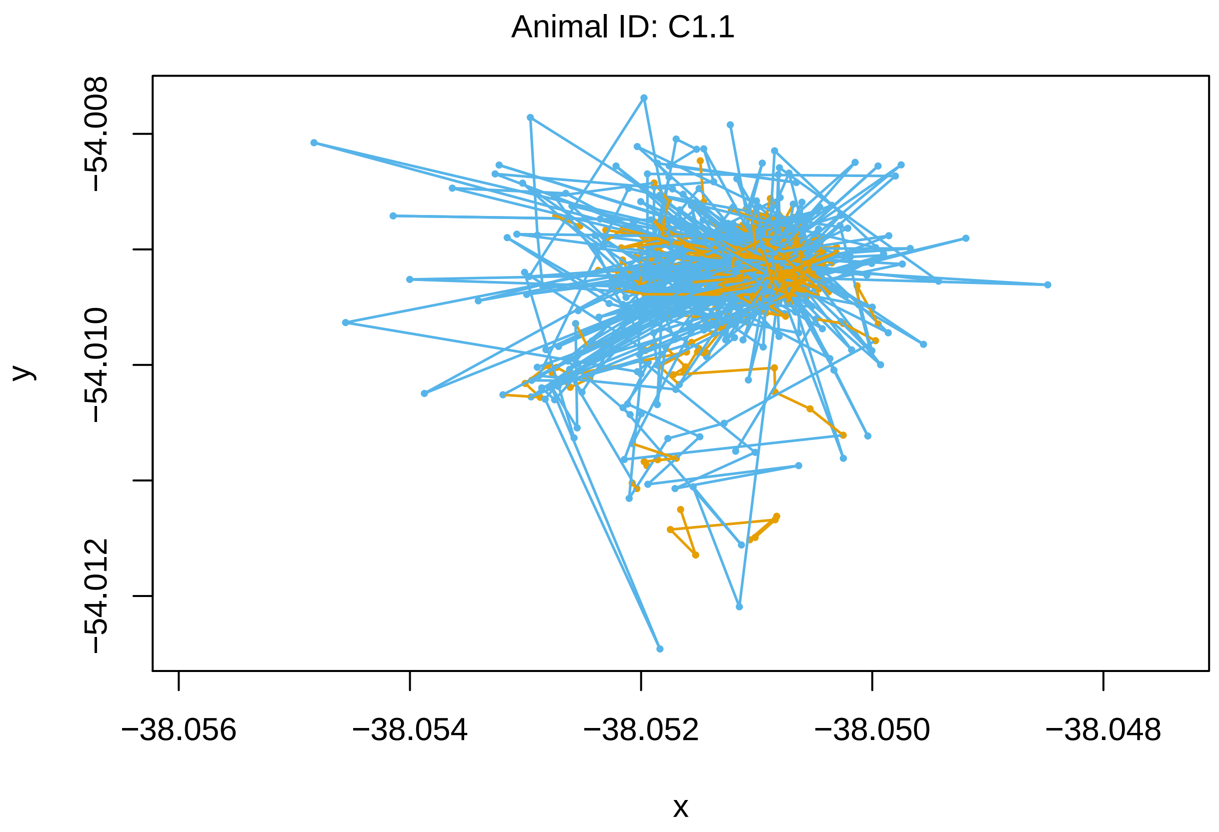


```
# model checking using pseudo-residuals
plotPR(mod_covsAll)
```

```
## Computing pseudo-residuals... DONE
```

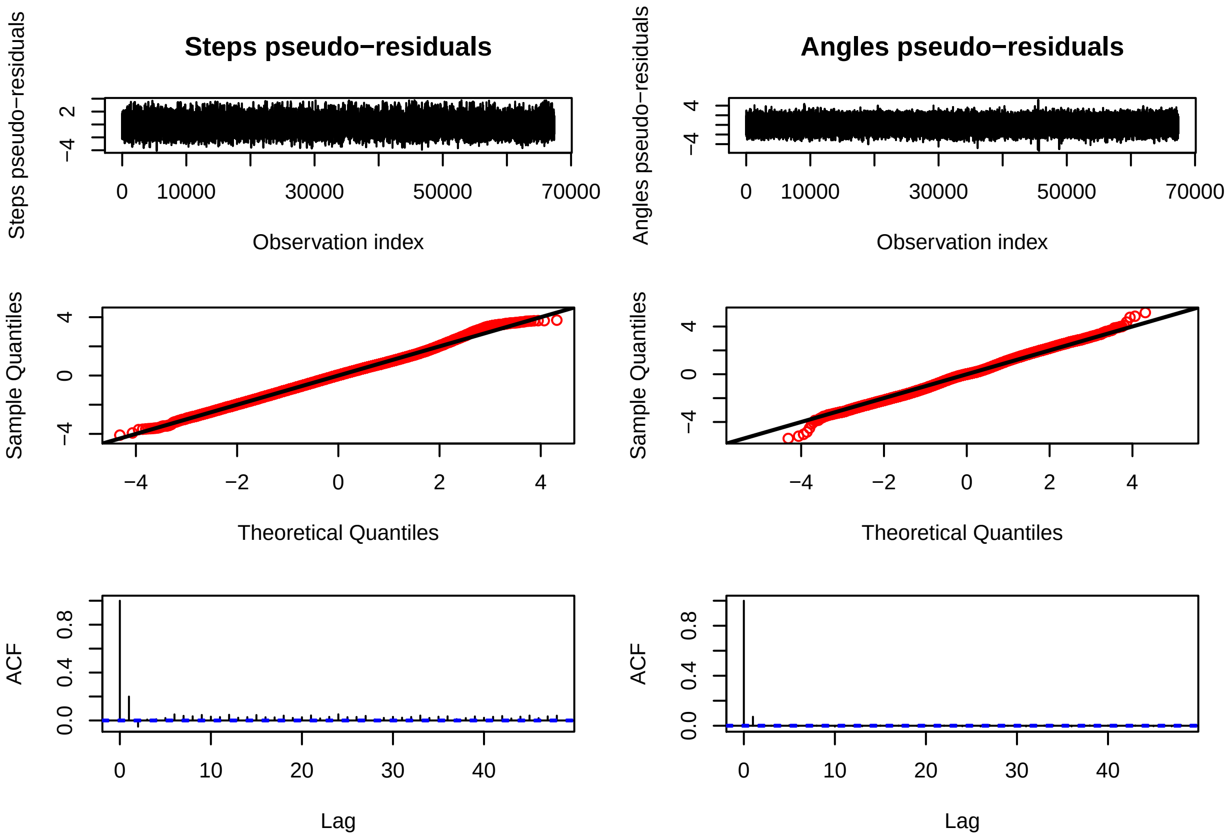


```r
## final model: 2-state HMM including all covariates & excluding turning angle
mod_final <- fitHMM(allData, nbStates = 2, stepDist = "gamma", angleDist = "none",
    stepPar0 = as.vector(t(mod0$mle$stepPar)), stationary = F, verbose = 0, formula =
    ~Sex01 + Age_sd + I(Age_sd^2) + CI_sd + sin((2 * pi * Hour)/24) + cos((2 * pi *
    Hour)/24) + Year + DRYBULB_TEMPERATURE_sd + WIND_SPEED_sd + Beach01 + Sex01:Beach01 +
    Age_sd:Beach01 + I(Age_sd^2):Beach01 + CI_sd:Beach01 + sin((2 * pi *
    Hour)/24):Beach01 + cos((2 * pi * Hour)/24):Beach01 + Year:Beach01 +
    DRYBULB_TEMPERATURE_sd:Beach01 + WIND_SPEED_sd:Beach01)

mod_final
```

```
## Value of the maximum log-likelihood: -296137.5
##
## Step length parameters:
## ----------------------
##         state 1  state 2
## mean 22.45869 75.83902
## sd   15.90470 52.66545
##
##
## Regression coeffs for the transition probabilities:
## --------------------------------------------------
##                                    1 -> 2       2 -> 1
## intercept                     -1.79020245 -1.355832078
## Sex01                          0.14276657  0.047251121
## Age_sd                         0.05819978  0.067185071
```

```
## I(Age_sd^2)                       -0.04234757 -0.063293666
## CI_sd                              0.08643966 -0.019570011
## sin((2 * pi * Hour)/24)            0.02382723 -0.114680054
## cos((2 * pi * Hour)/24)           -0.22454925  0.072597950
## Year                               0.19709997  0.171305182
## DRYBULB_TEMPERATURE_sd            -0.09333160 -0.001583425
## WIND_SPEED_sd                     -0.04389531 -0.028180310
## Beach01                            0.36642745  0.252634745
## Sex01:Beach01                     -0.24421560 -0.162419860
## Age_sd:Beach01                     0.32194051 -0.176124665
## I(Age_sd^2):Beach01               -0.15134668  0.014340439
## CI_sd:Beach01                      0.03354269  0.149429716
## sin((2 * pi * Hour)/24):Beach01 -0.06950136  0.065653451
## cos((2 * pi * Hour)/24):Beach01 -0.08596348  0.208641595
## Year:Beach01                      -0.58935835 -0.057858517
## DRYBULB_TEMPERATURE_sd:Beach01    0.12157638  0.021865225
## WIND_SPEED_sd:Beach01              0.02972298  0.053187454
##
## Initial distribution:
## --------------------
## [1] 0.7190357 0.2809643
```

```
plot(mod_final, animals = 1)
```

```
## Decoding states sequence... DONE
```

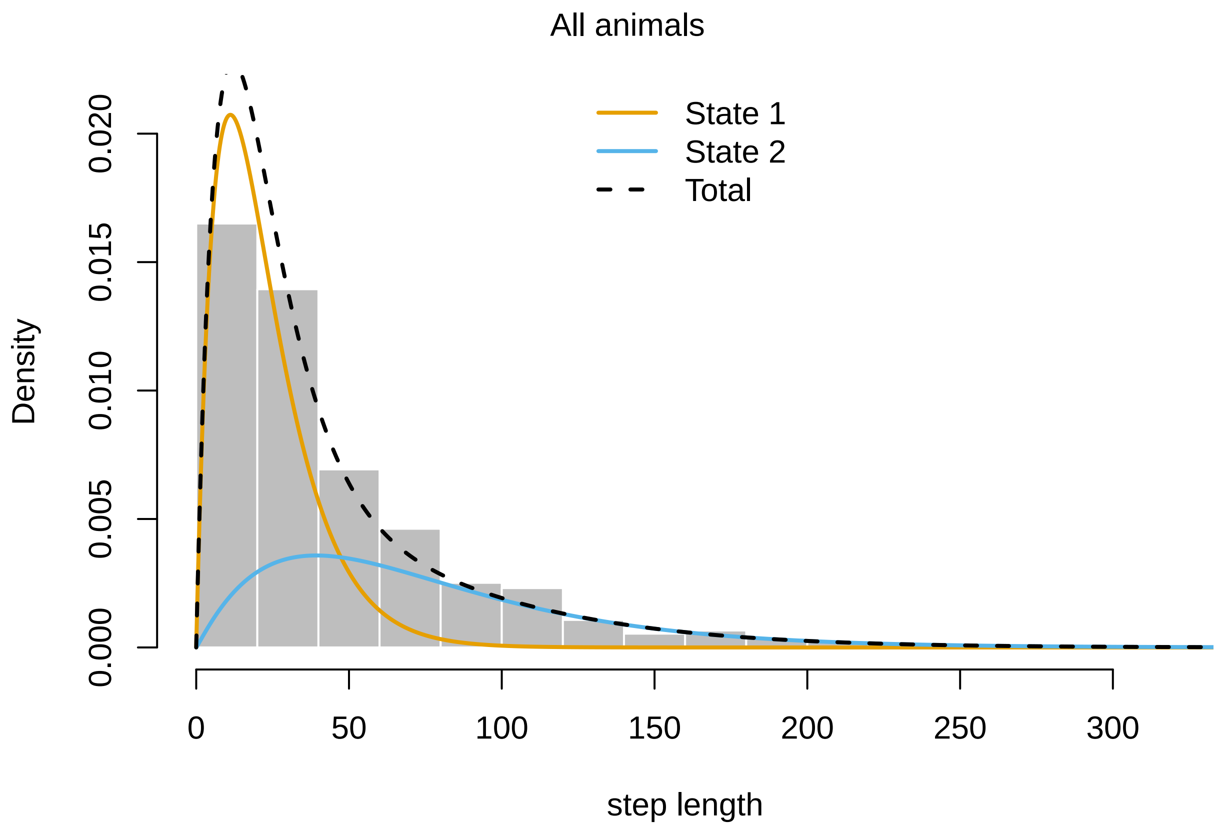

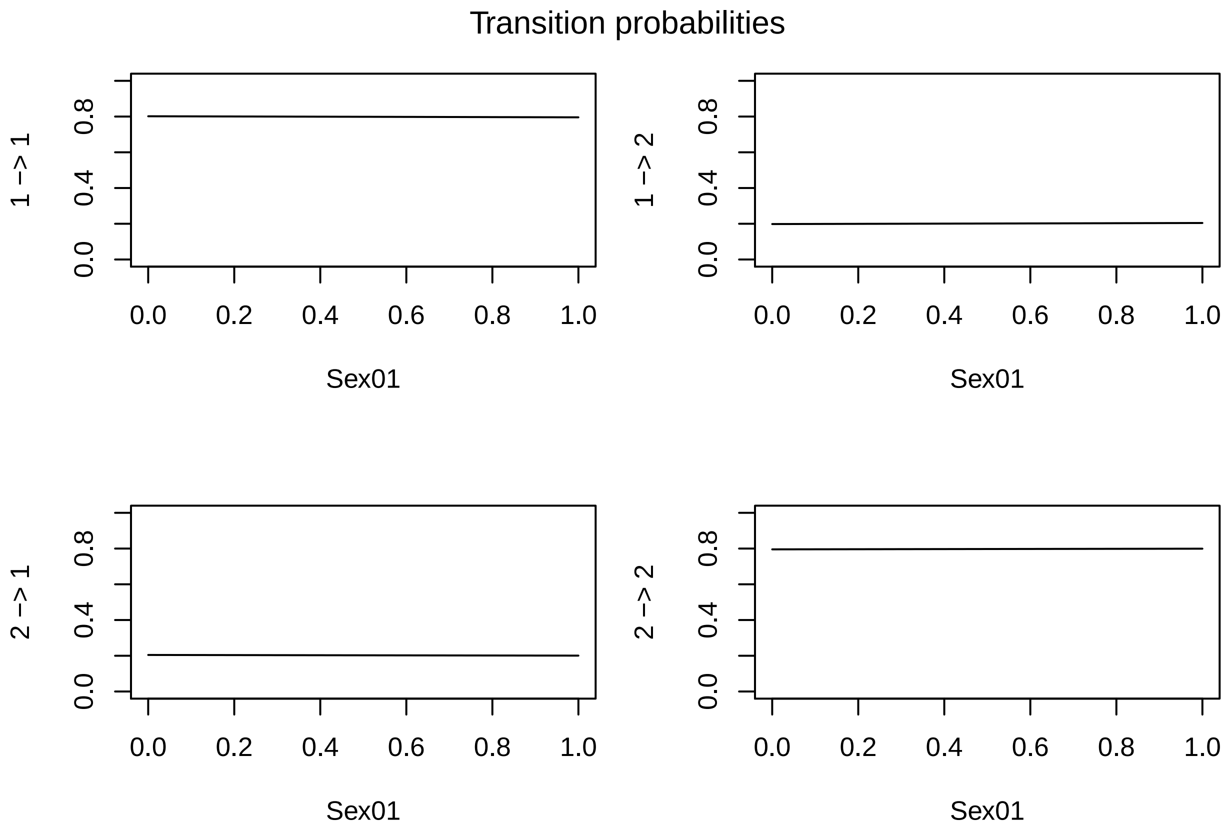
Transition probabilities
1 −> 1
1 −> 2
2 −> 1
2 −> 2
0.0
0.4
0.8
0.0 0.2 0.4 0.6 0.8 1.0
Sex01

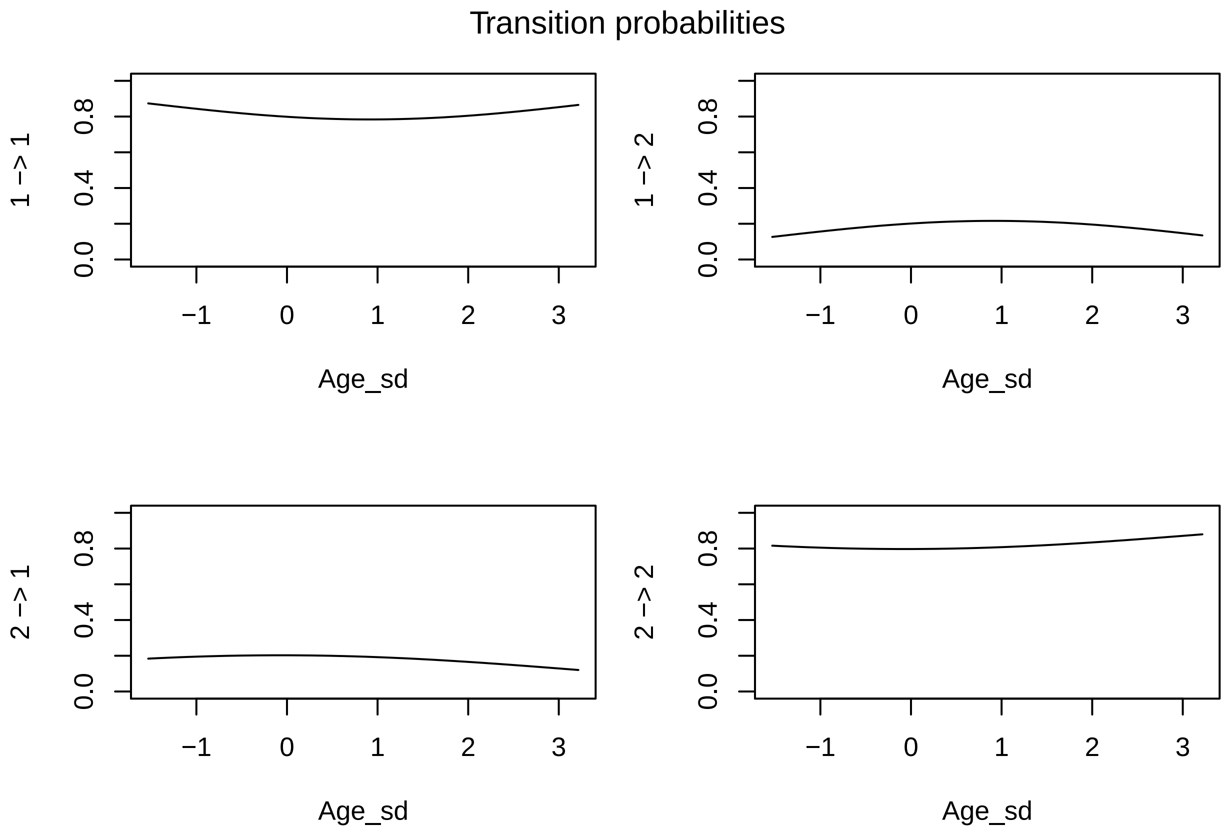

Transition probabilities
1 −> 1
1 −> 2
2 −> 1
2 −> 2
0.0
0.4
0.8
−1
0
1
2
3
Age_sd

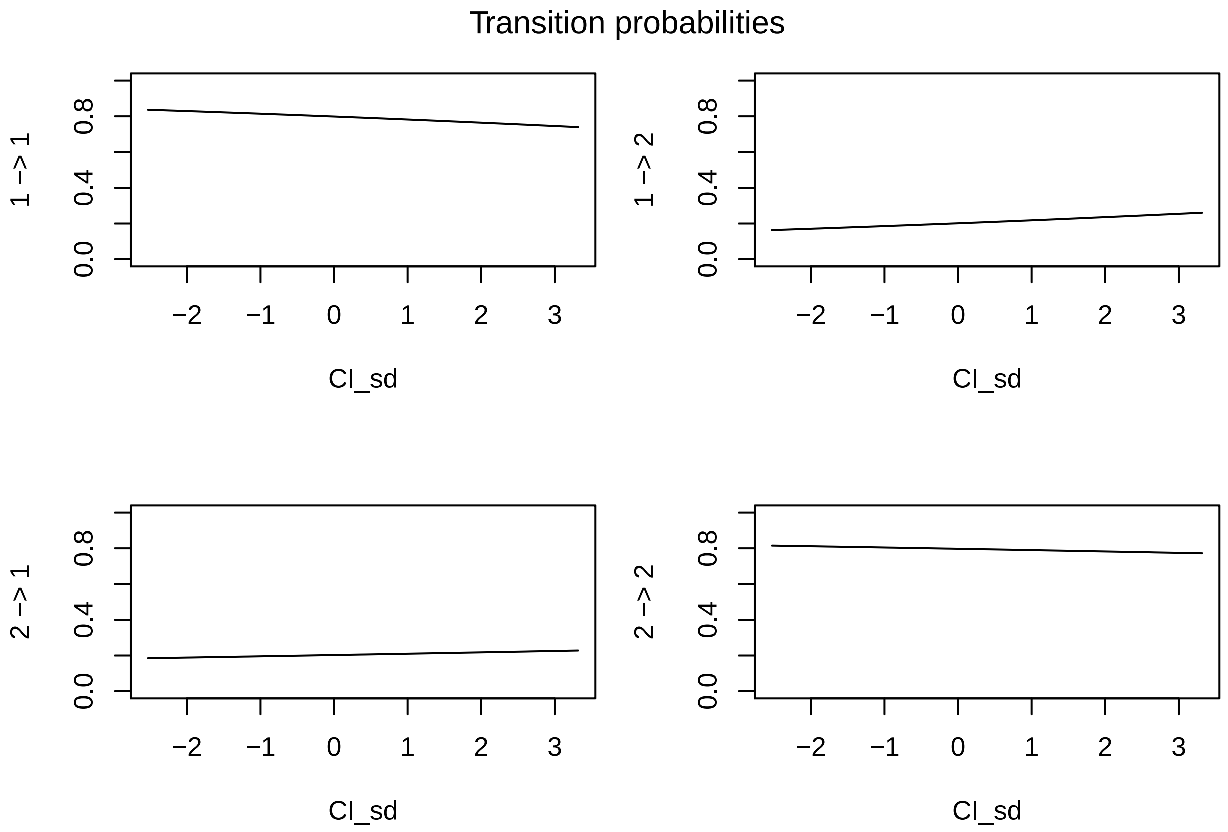
Transition probabilities
1 −> 1
1 −> 2
2 −> 1
2 −> 2
0.0
0.4
0.8
−2
−1
0
1
2
3
CI_sd

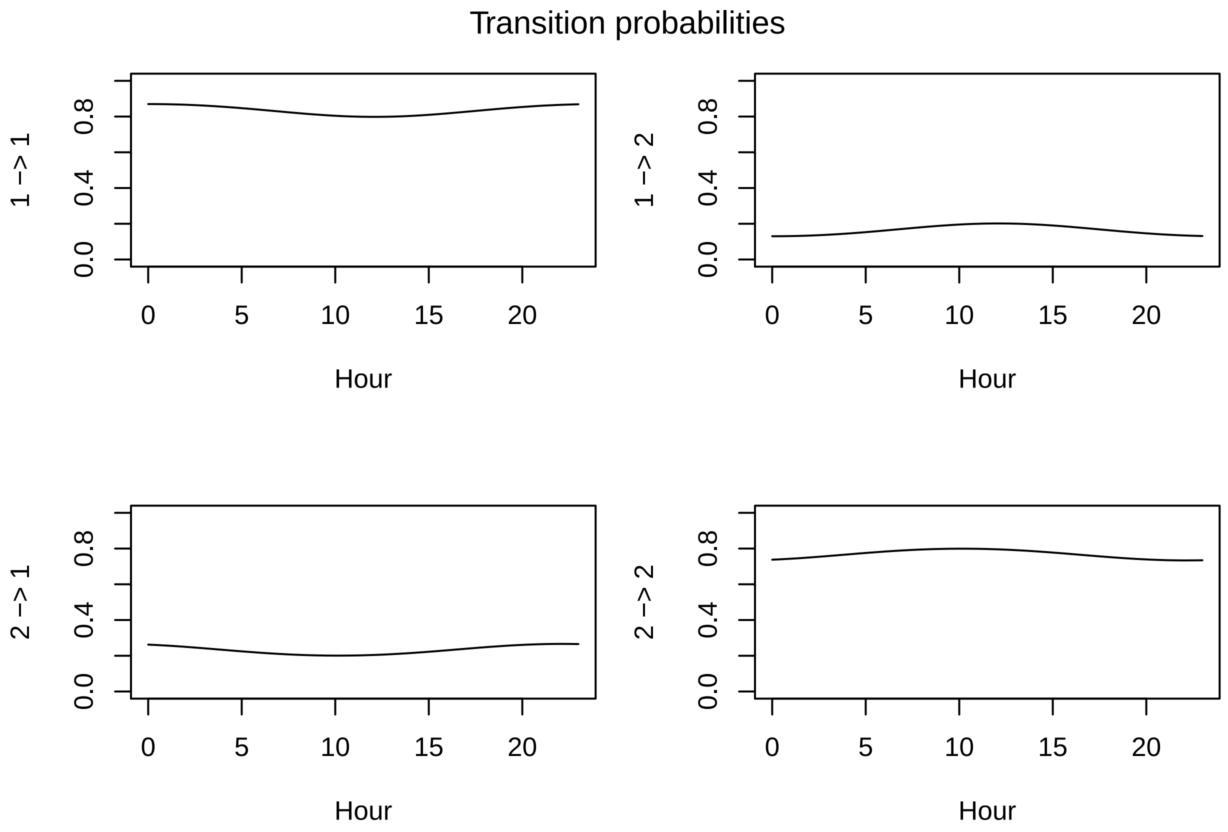

Transition probabilities
1 −> 1
1 −> 2
2 −> 1
2 −> 2
0.0
0.4
0.8
0
5
10
15
20
Hour

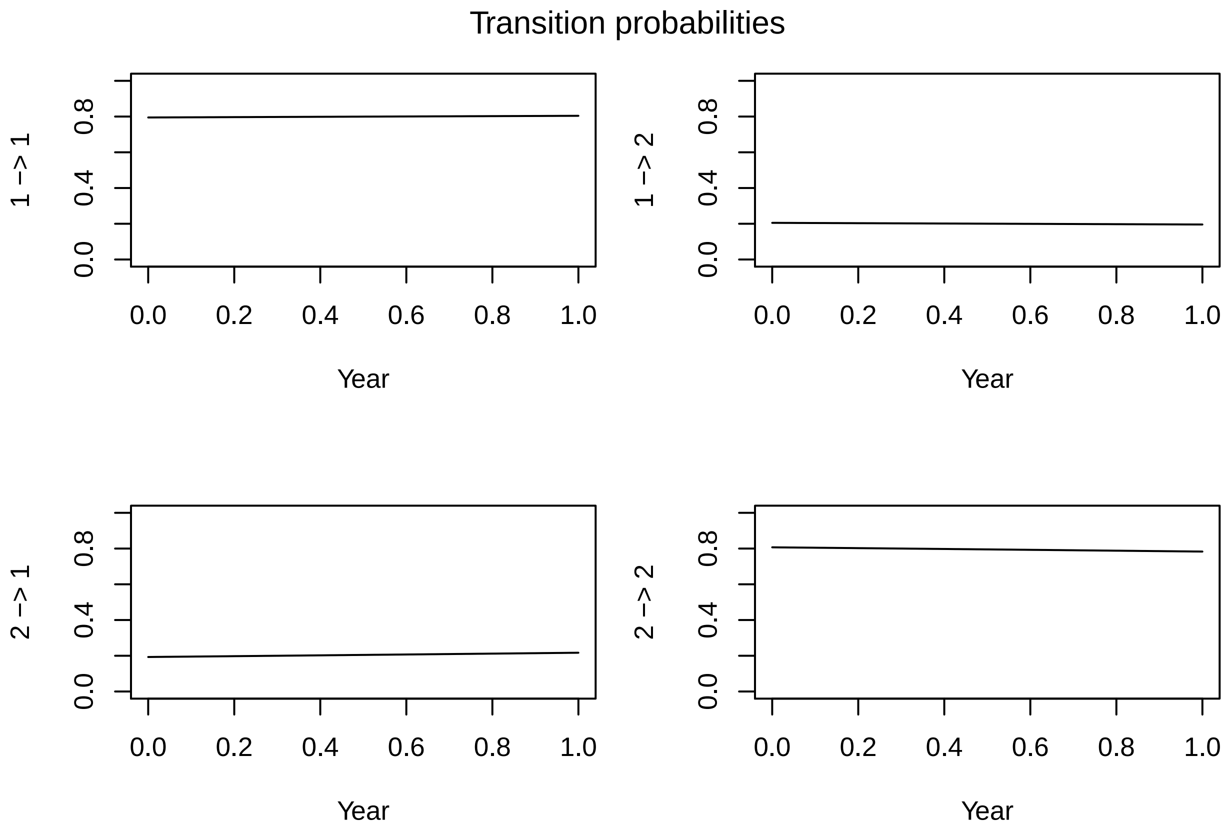
Transition probabilities
1 −> 1
1 −> 2
2 −> 1
2 −> 2
0.0
0.4
0.8
0.0
0.2
0.4
0.6
0.8
1.0
Year

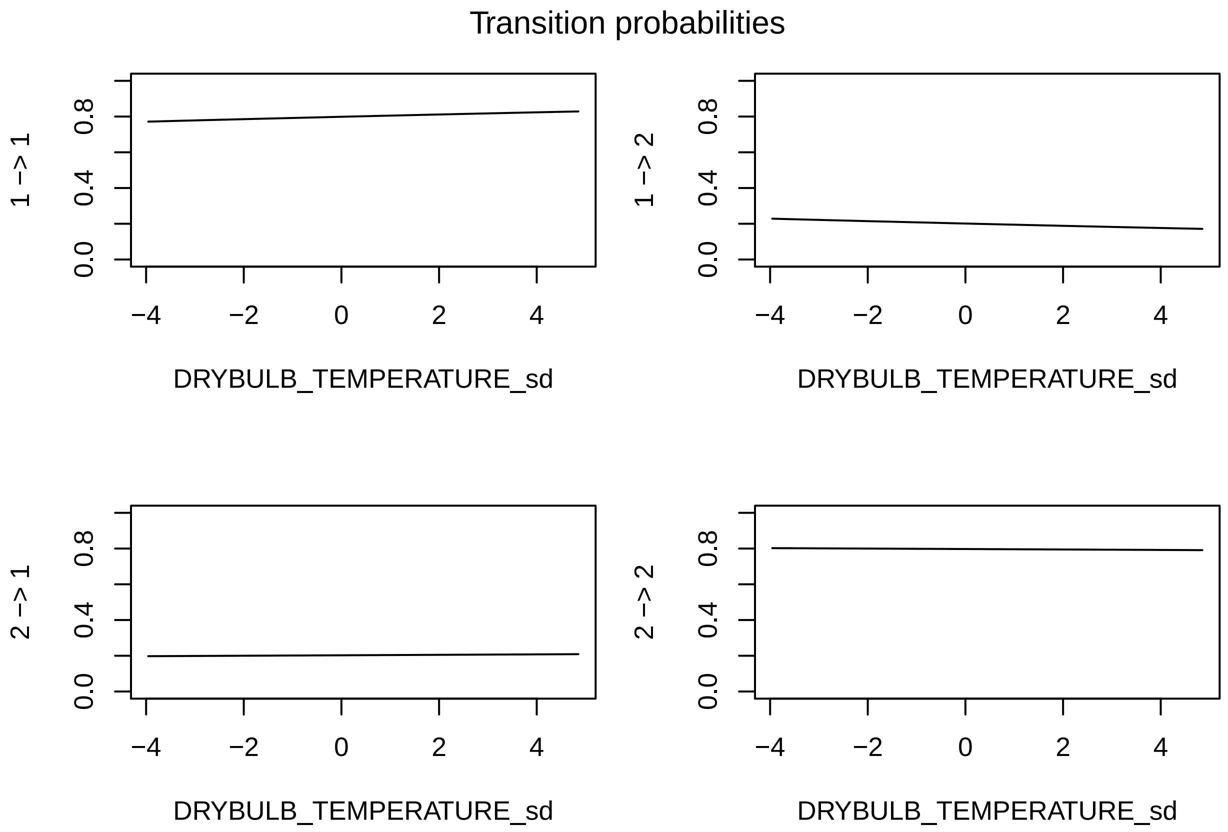
Transition probabilities
1 −> 1
1 −> 2
2 −> 1
2 −> 2
0.0
0.4
0.8
−4
−2
0
2
4
DRYBULB_TEMPERATURE_sd

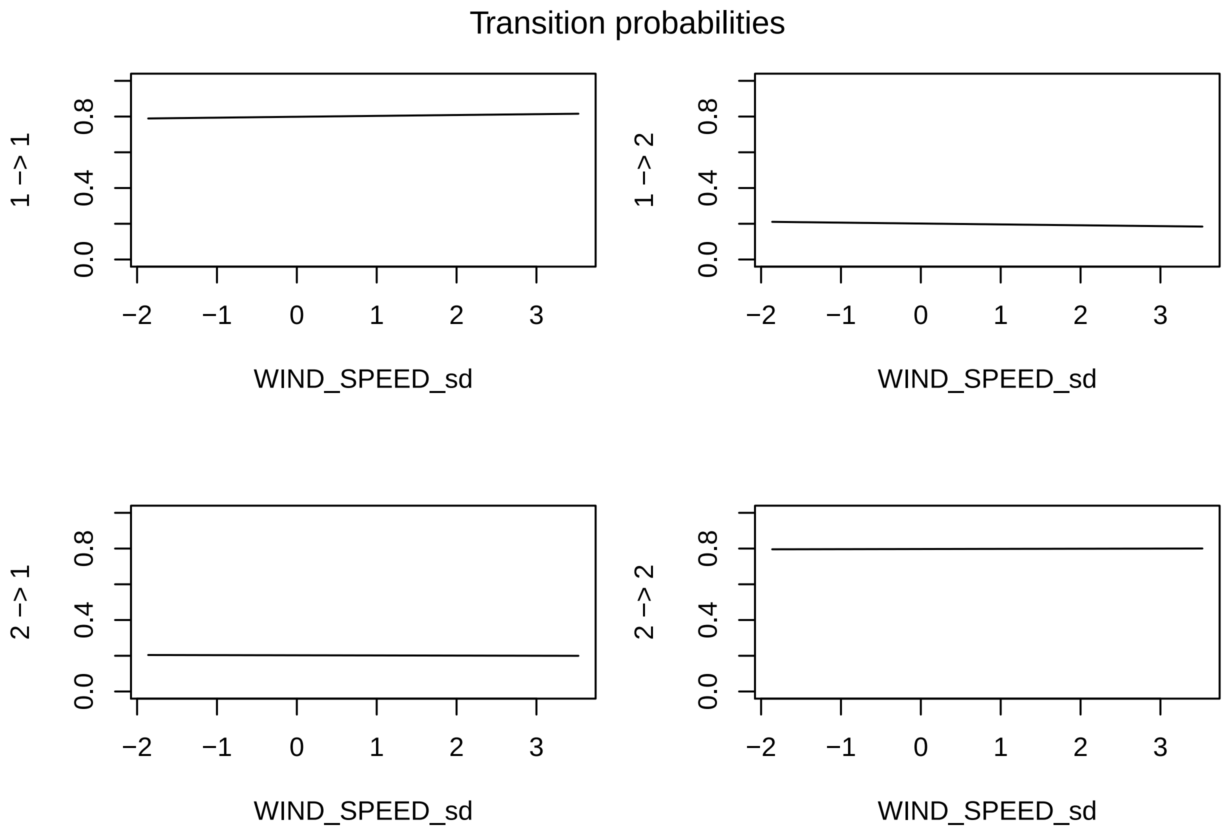
Transition probabilities
1 −> 1
1 −> 2
2 −> 1
2 −> 2
0.0
0.4
0.8
−2
−1
0
1
2
3
WIND_SPEED_sd

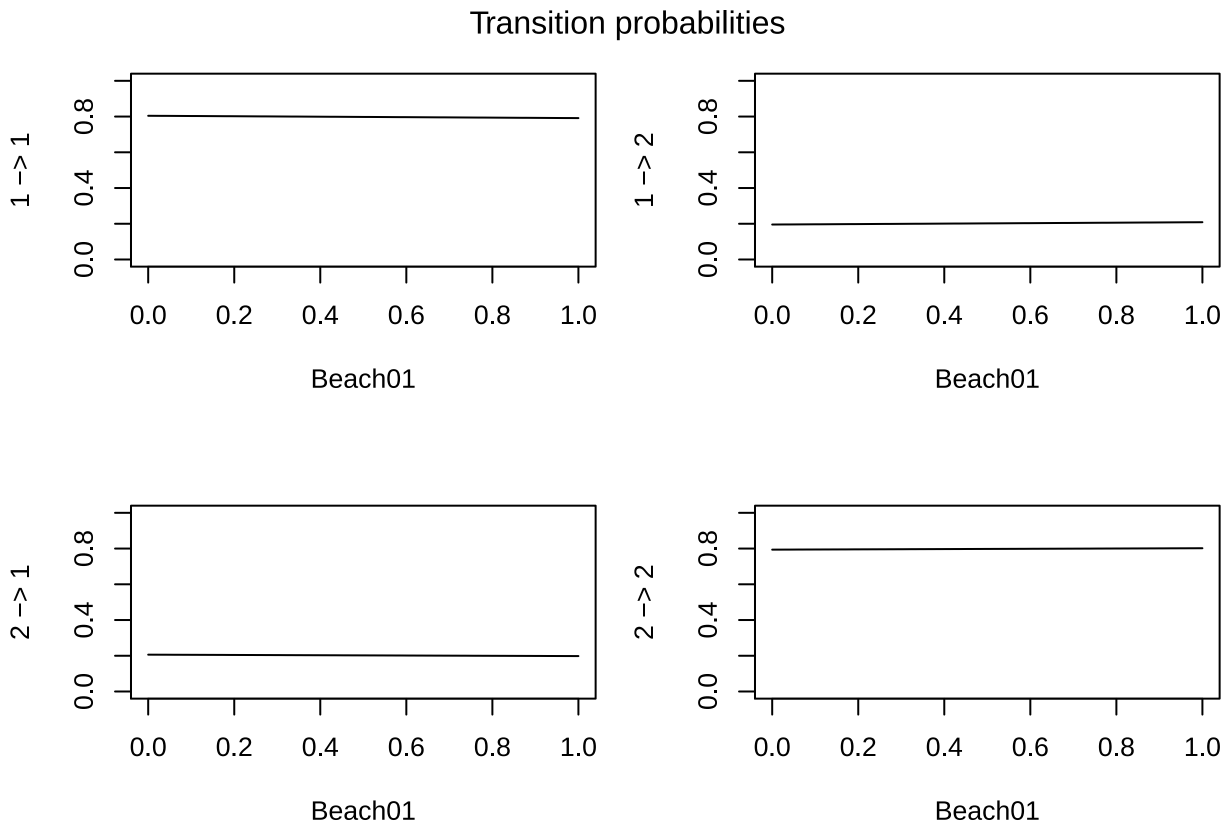

Transition probabilities
1 -> 1
1 -> 2
2 -> 1
2 -> 2
0.0
0.4
0.8
0.0
0.2
0.4
0.6
0.8
1.0
Beach01

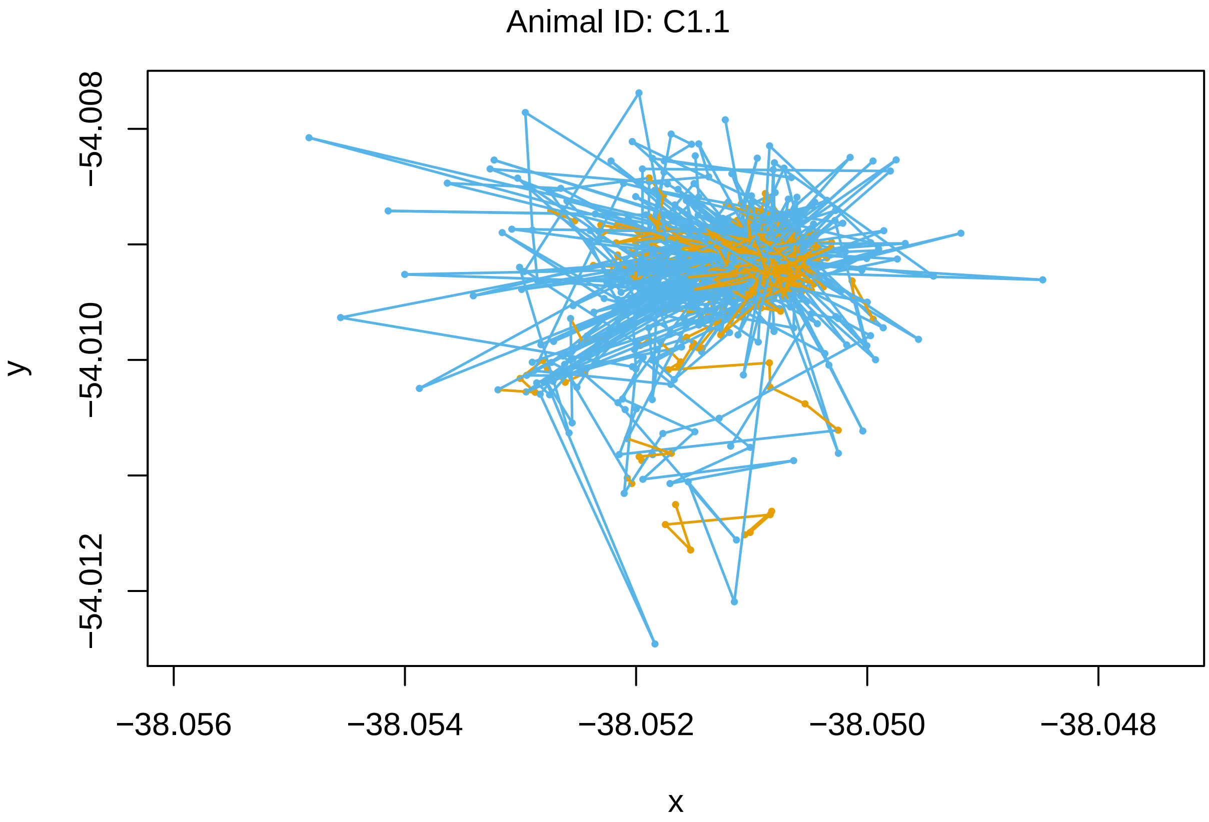


```r
# model checking using pseudo-residuals
plotPR(mod_final)
```

```
## Computing pseudo-residuals... DONE
```

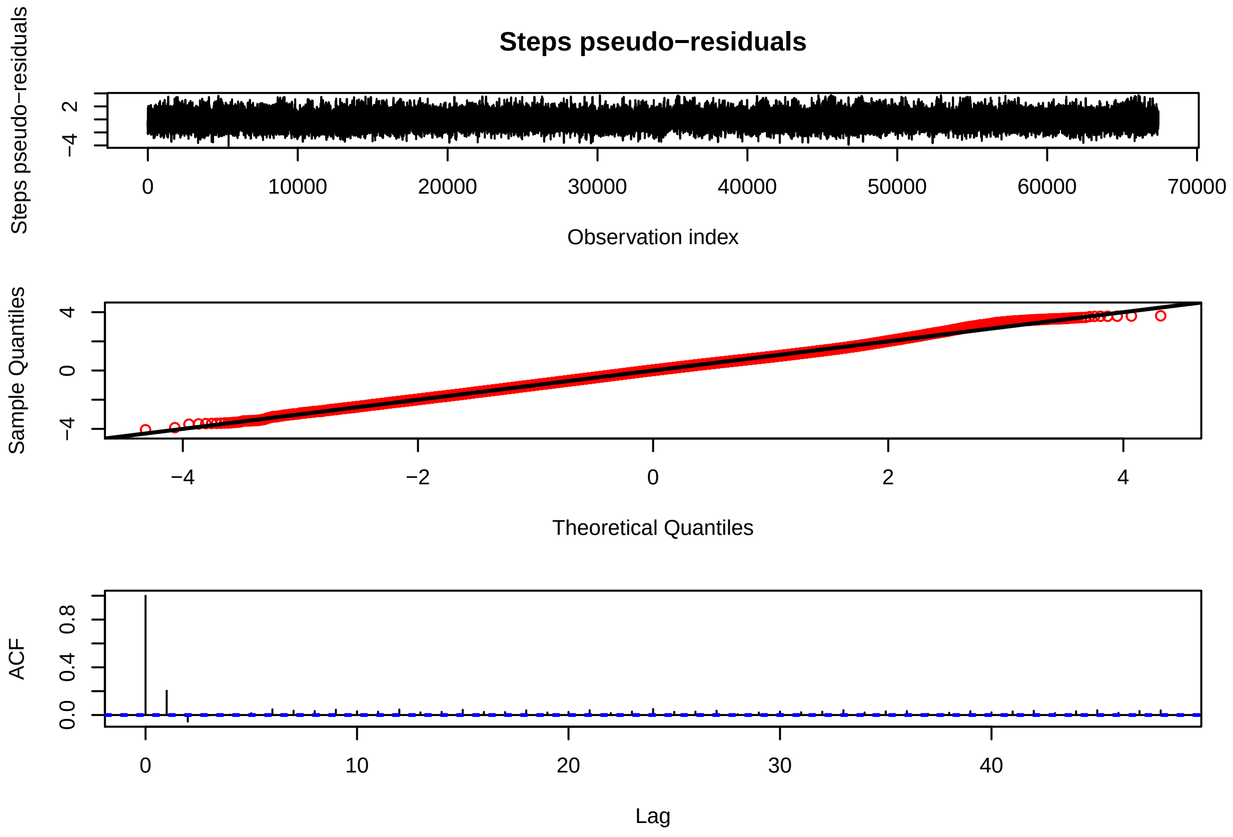


```
AIC(mod_final)
```

```
## [1] 592365
```

```
# compare decoded states for univariate & bivariate HMMs
allData$state <- viterbi(mod_final)
states_wAngle <- viterbi(mod_covsAll)
# percent of observations that get decoded differently
sum(allData$state != states_wAngle)/nrow(allData)
```

```
## [1] 0.03638548
```

Overview of final dataset

A broad overview of the final dataset is provided, including the percent weight of GPS and VHF tags relative to pup birth mass. Final sample size of the dataset by beach and season, number of moralities and deployment durations are calculated. The average temperature per season is calculated.

```
# read in data
movement <- read.csv("furSeals_finalData.csv", header = TRUE, sep = ",")
```

```r
temp <- read.csv("Bird Island - Wind_Temp_2018-2020.csv", header = TRUE, sep = ",")

# percent weight of tags relative to pup birth mass
tags <- 36 + 16 # weight of VHF + GPS in grams
weight_birth <- movement[movement$Age == 0, ]
weight_birth <- do.call(rbind, lapply(split(weight_birth, weight_birth$IDno), head, 1))
weight_birth <- mean(weight_birth$Weight_10Days) * 1000 # avg weight of pups at birth (g)
(tags * 100)/weight_birth # percentage weight of tags to pup mass at birth
```

```
## [1] 0.9306167
```

```r
## Sample size by beach, season
nrow(table(movement$IDno[movement$Year == 0]))
```

```
## [1] 40
```

```r
nrow(table(movement$IDno[movement$Year == 1]))
```

```
## [1] 26
```

```r
nrow(table(movement$IDno[movement$Beach == "FWB"]))
```

```
## [1] 32
```

```r
nrow(table(movement$IDno[movement$Beach == "SSB"]))
```

```
## [1] 34
```

```r
## how many pups die?
nrow(table(movement$IDno[movement$Death == "N"]))
```

```
## [1] 53
```

```r
## Time range of sampling
range <- setDT(movement)[, .SD[which.max(Age)], by = IDno]
range(range$Age)
```

```
## [1]  4 80
```

```r
# for pups that survive
range(range[range$Death == "N"]$Age)
```

```
## [1] 20 80
```

```r
median(range[range$Death == "N"]$Age)
```

```
## [1] 51
```

```r
mean(range[range$Death == "N"]$Age)
```

```
## [1] 51.69811
```

```r
# for pups that die
range[range$Death == "Y"]$Age
```

```
##  [1] 22 27 40  4 11 29 41 39  4 13  4 15  6
```

```r
median(range[range$Death == "Y"]$Age)
```

```
## [1] 15
```

```r
mean(range[range$Death == "Y"]$Age)
```

```
## [1] 19.61538
```

```r
table(range[range$Death == "Y"]$Beach)
```

```
##
## FWB SSB
##  10   3
```

```r
## average temperature per season
mean(temp[temp$Season == "1819", ]$DRYBULB_TEMPERATURE)
```

```
## [1] 3.665316
```

```r
sd(temp[temp$Season == "1819", ]$DRYBULB_TEMPERATURE)
```

```
## [1] 1.27667
```

```r
mean(temp[temp$Season == "1920", ]$DRYBULB_TEMPERATURE)
```

```
## [1] 3.946392
```

```r
sd(temp[temp$Season == "1920", ]$DRYBULB_TEMPERATURE)
```

```
## [1] 1.43429
```

Post-hoc analysis

To determine whether additional variables that we were not able to include in the HMM explain a significant proportion of variation in activity, we built two generalised linear mixed models (GLMMs) post-hoc.

Pup mortality

We test whether the survival status of each pup (0 = died, 1 = survived) explains a significant proportion of the variation in pup activity levels ('states'). We wanted to test for an association between survival status and movement without assuming the direction of causality. Pup ID was included as a random effect to account for repeated measurements of individuals.

```r
## data preparation state 1 = short step length (inactive) & state 2 = long step
## length (active)
movement$state <- factor(movement$state, ordered = FALSE)
movement$Death <- as.factor(movement$Death)

## Modeling - Death (y/n)
m1 <- glmer(state ~ Death + (1 | IDno), family = "binomial", data = movement)
summary(m1)
```

```
## Generalized linear mixed model fit by maximum likelihood (Laplace
##   Approximation) [glmerMod]
##  Family: binomial  ( logit )
## Formula: state ~ Death + (1 | IDno)
##    Data: movement
##
##      AIC      BIC   logLik deviance df.resid
##  85259.1  85286.4 -42626.5  85253.1    67414
##
## Scaled residuals:
##     Min      1Q  Median      3Q     Max
## -1.0856 -0.8121 -0.5669  1.1034  4.6183
##
## Random effects:
##  Groups Name        Variance Std.Dev.
##  IDno   (Intercept) 0.368    0.6066
## Number of obs: 67417, groups:  IDno, 66
##
## Fixed effects:
##             Estimate Std. Error z value Pr(>|z|)
## (Intercept) -0.55972    0.08382  -6.677 2.43e-11 ***
## DeathY      -0.75826    0.19248  -3.939 8.17e-05 ***
## ---
## Signif. codes:  0 '***' 0.001 '**' 0.01 '*' 0.05 '.' 0.1 ' ' 1
##
## Correlation of Fixed Effects:
##        (Intr)
## DeathY -0.430
```

```r
## Residual Checks 1. qq-plot to detect overall deviations from the expected
## distribution 2. plot of the residuals against the predicted value

m1.simulationOutput <- simulateResiduals(fittedModel = m1, plot = F)
# over/underdispersion ?
testDispersion(m1.simulationOutput)
```

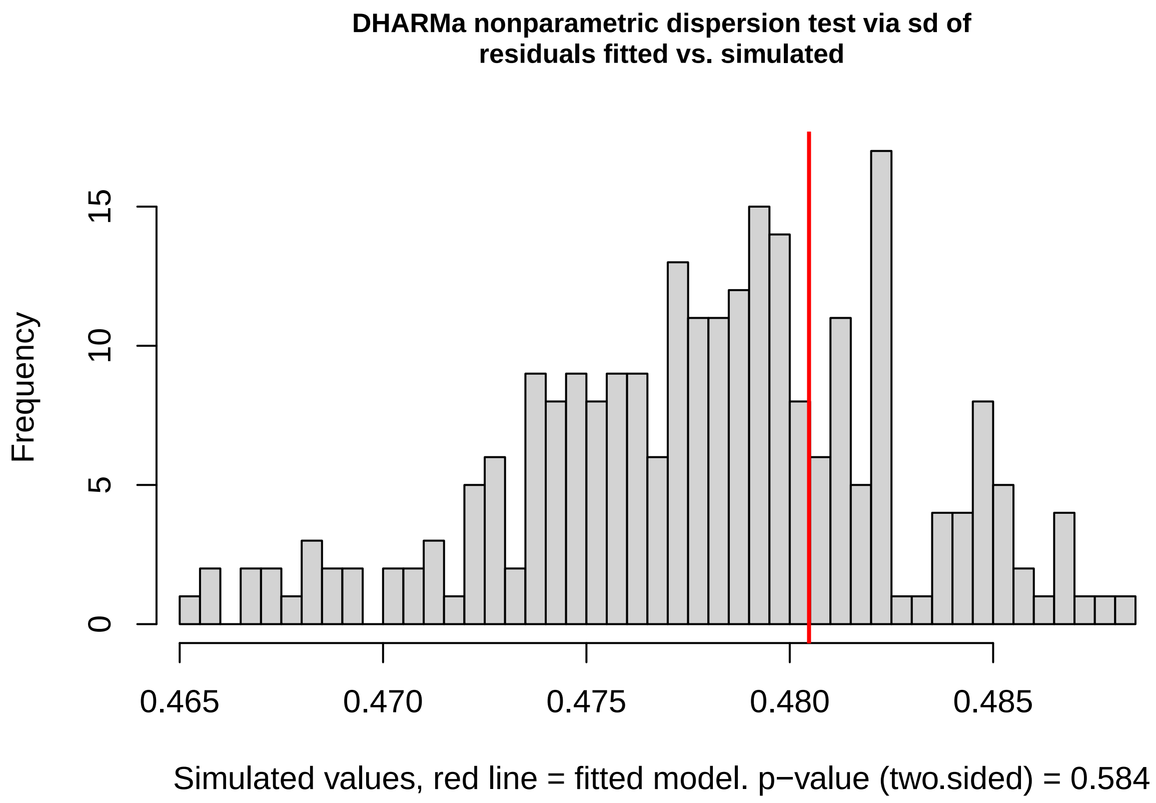


```
##
##  DHARMa nonparametric dispersion test via sd of residuals fitted vs.
##  simulated
##
## data:  simulationOutput
## ratioObsSim = 1.0052, p-value = 0.584
## alternative hypothesis: two.sided
```

```
## calculate percentage of time pups were in each state
per.act.death <- data.frame(death = c("Mortality", "Survived"), state1 =
    c(table(movement$state[movement$Death == "Y"])[1],
    table(movement$state[movement$Death == "N"])[1]), state2 =
    c(table(movement$state[movement$Death == "Y"])[2],
    table(movement$state[movement$Death == "N"])[2]))
per.act.death$tot <- with(per.act.death, state1 + state2)
per.act.death <- gather(per.act.death, state, value, state1:state2, factor_key = TRUE)
per.act.death$p <- with(per.act.death, value/tot)
per.act.death$per <- with(per.act.death, paste(formatC(p * 100, digits = 2, format =
    "fg"), "%", sep = ""))
```

Attendance of mother

We test whether maternal attendance (0 = present ashore, 1 = absent) explains a significant proportion of the variation in pup activity levels ('states'). As data on maternal attendance were only available for

pups born on FWB, this covariate could not be included in the full HMM, which defined an interaction term between all covariates and 'colony'. Pup ID was included as a random effect to account for repeated measurements of individuals.

```r
## read in data
attendance <- read.csv("Attendance_FWB.csv", header = TRUE, sep = ",")
## data preparation
attendance$state <- as.factor(attendance$state)
attendance$MumAshore <- as.factor(attendance$MumAshore)
## Modeling - Attendance (y/n), just FWB pups
m2 <- glmer(state ~ MumAshore + (1 | IDno), family = "binomial", data = attendance)
summary(m2)
```

```
## Generalized linear mixed model fit by maximum likelihood (Laplace
##   Approximation) [glmerMod]
##  Family: binomial  ( logit )
## Formula: state ~ MumAshore + (1 | IDno)
##    Data: attendance
##
##      AIC      BIC   logLik deviance df.resid
##  29503.4  29527.5 -14748.7  29497.4    22974
##
## Scaled residuals:
##     Min      1Q  Median      3Q     Max
## -1.0800 -0.7971 -0.6080  1.1473  2.9901
##
## Random effects:
##  Groups Name        Variance Std.Dev.
##  IDno   (Intercept) 0.2335   0.4832
## Number of obs: 22977, groups:  IDno, 24
##
## Fixed effects:
##             Estimate Std. Error z value Pr(>|z|)
## (Intercept) -0.59876    0.10003  -5.986 2.16e-09 ***
## MumAshore1  -0.09073    0.04218  -2.151   0.0315 *
## ---
## Signif. codes:  0 '***' 0.001 '**' 0.01 '*' 0.05 '.' 0.1 ' ' 1
##
## Correlation of Fixed Effects:
##            (Intr)
## MumAshore1 -0.056
```

```r
## Residual Checks 1. qq-plot to detect overall deviations from the expected
## distribution 2. plot of the residuals against the predicted value
m2.simulationOutput <- simulateResiduals(fittedModel = m2, plot = F)
# over/underdispersion ?
testDispersion(m2.simulationOutput)
```

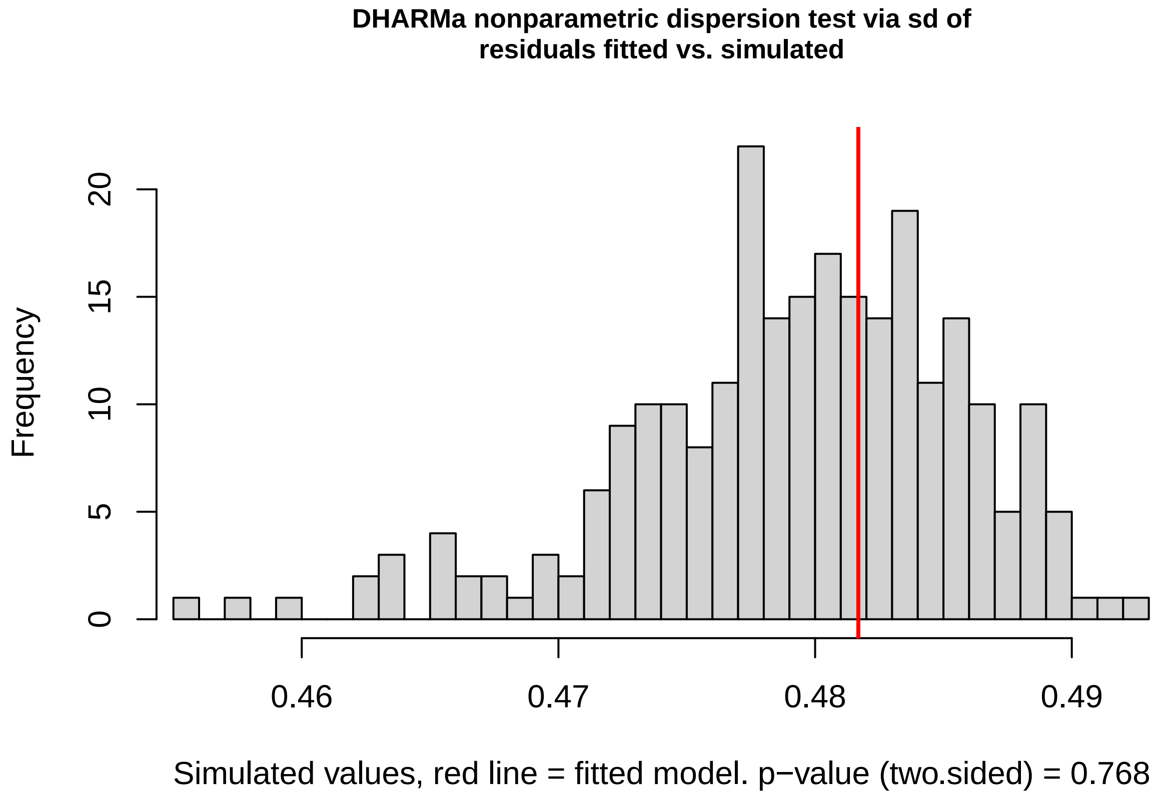


```
##
##  DHARMa nonparametric dispersion test via sd of residuals fitted vs.
##  simulated
##
## data:  simulationOutput
## ratioObsSim = 1.0051, p-value = 0.768
## alternative hypothesis: two.sided
```

```
## calculate percentage of time pups were in each state
per.act.ashore <- data.frame(attendance = c("Ashore", "Foraging"), state1 =
    c(table(attendance$state[attendance$MumAshore == 1])[1],
    table(attendance$state[attendance$MumAshore == 0])[1]), state2 =
    c(table(attendance$state[attendance$MumAshore == 1])[2],
    table(attendance$state[attendance$MumAshore == 0])[2]))
per.act.ashore$tot <- with(per.act.ashore, state1 + state2)
per.act.ashore <- gather(per.act.ashore, state, value, state1:state2, factor_key = TRUE)
per.act.ashore$p <- with(per.act.ashore, value/tot)
per.act.ashore$per <- with(per.act.ashore, paste(formatC(p * 100, digits = 2, format =
    "fg"), "%", sep = ""))
```

Manuscript Figures

Figure 2

(a) Location of the two fur seal breeding colonies - Freshwater Beach (FWB) and Special Study Beach (SSB) - from which pups were tagged. (b) Decoded states from the HMM showing the inferred activity patterns of pups. (c) GPS locations recorded throughout ontogeny for 32 pups born on FWB and 34 pups born on SSB.

```
## Figure 2a - mapping Bird Island read in data - Bird Island maps (from BAS,
## MAGIC) & outline of ssb and fwb (made in Google Earth)
bi_coast <- st_read("Map_BirdIsland/BI_Coast_Projected_new.shp")
```

```
## Reading layer `BI_Coast_Projected_new' from data source `C:\Users\localadmin\Documents\3 UniversitÃ¤
## Simple feature collection with 28 features and 5 fields
## geometry type:  POLYGON
## dimension:      XY
## bbox:           xmin: -38.08212 ymin: -54.02197 xmax: -38.00766 ymax: -53.99703
## geographic CRS: WGS 84
```

```
ssb <- st_read("SSB.kml/doc.kml")
```

```
## Reading layer `SSB.kmz' from data source `C:\Users\localadmin\Documents\3 UniversitÃ¤t Bielefeld\_Pr
## Simple feature collection with 1 feature and 2 fields
## geometry type:  POLYGON
## dimension:      XYZ
## bbox:           xmin: -38.05125 ymin: -54.01178 xmax: -38.05022 ymax: -54.01133
## z_range:        zmin: 0 zmax: 0
## geographic CRS: WGS 84
```

```
fwb <- st_read("FWB.kml/doc.kml")
```

```
## Reading layer `FWB.kmz' from data source `C:\Users\localadmin\Documents\3 UniversitÃ¤t Bielefeld\_Pr
## Simple feature collection with 1 feature and 2 fields
## geometry type:  POLYGON
## dimension:      XYZ
## bbox:           xmin: -38.05219 ymin: -54.00916 xmax: -38.05081 ymax: -54.00836
## z_range:        zmin: 0 zmax: 0
## geographic CRS: WGS 84
```

```
## plotting
ggplot() + geom_sf(data = bi_coast, fill = "#d3d3d3") + theme(legend.position = "none") +
    xlab("") + ylab("") + theme_void() + annotate(geom = "rect", xmin = -38.06, xmax =
    -38.045, ymin = -54.014, ymax = -54.005, fill = NA, color = "black")
```

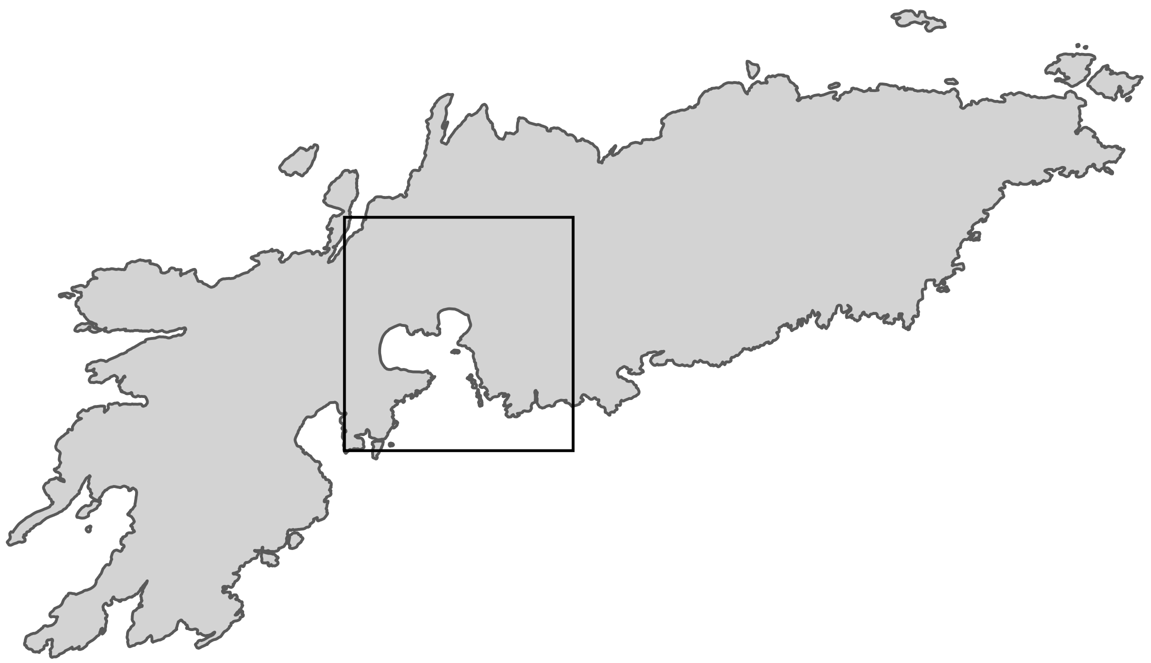

```r
ggplot() + geom_sf(data = bi_coast, fill = "white") + geom_sf(data = fwb, fill =
    "#377eb8") + geom_sf(data = ssb, fill = "#e41a1c") + theme(legend.position = "none")
    + labs(title = "a") + theme_void() + coord_sf(xlim = c(-38.06, -38.045), ylim =
    c(-54.014, -54.005), expand = FALSE) + annotation_scale(aes(location = "br", style =
    "ticks")) + theme(panel.border = element_rect(colour = "black", fill = NA, size = 1))
    + annotate(geom = "text", x = -38.05, y = -54.0092, label = "FWB", color = "#377eb8",
    fontface = "bold") + annotate(geom = "text", x = -38.05, y = -54.011, label = "SSB",
    color = "#e41a1c", fontface = "bold") + annotate(geom = "rect", xmin = -38.05, xmax =
    -38.08, ymin = -54.005, ymax = -54.0065, fill = "white", color = "white")
```

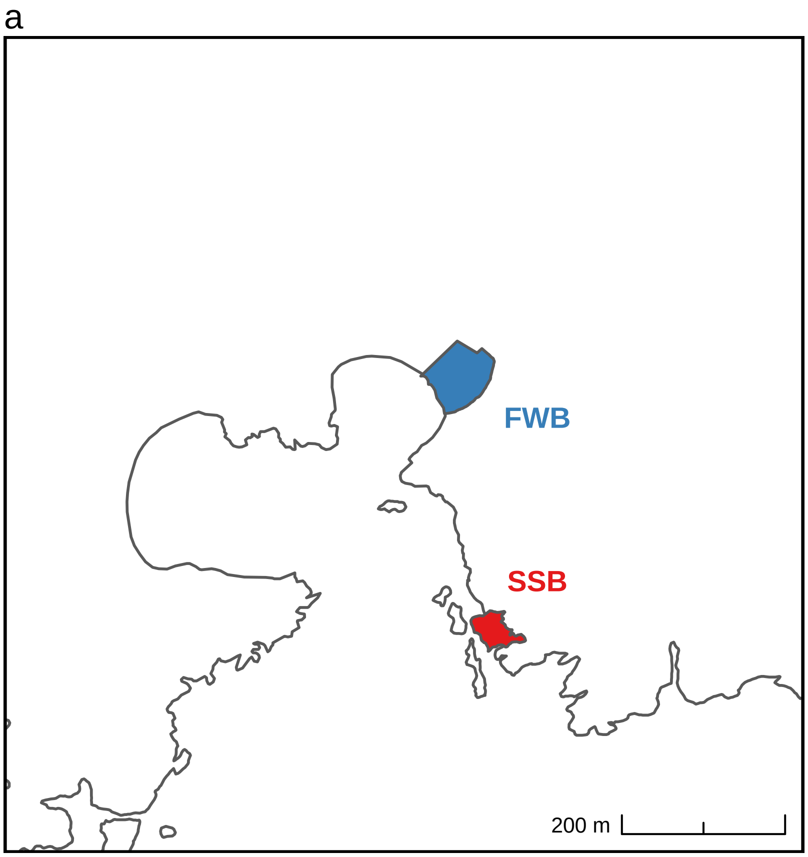


```r
## Figure 2b - All decoded states
id <- unique(allData$IDno)[1]
dataID <- allData[which(allData$IDno == id), ]
dataID$x[which(is.na(dataID$step))] <- dataID$y[which(is.na(dataID$step))] <- NA

cols <- rep("#E69F00", nrow(dataID))
cols[which(dataID$state == 2)] <- "#009E73"

plot.bi.states <- ggplot() + geom_path(data = dataID, aes(x = x, y = y), colour = cols,
    size = 0.2, na.rm = T, alpha = 0.2) + geom_point(data = dataID, aes(x = x, y = y),
    colour = cols, size = 0.1, na.rm = T, alpha = 0.2) + theme(legend.position = "none")
    + theme_void()

for (i in 2:length(unique(allData$ID))) { id <- unique(allData$ID)[i] dataID <-
    allData[which(allData$ID == id), ] dataID$x[which(is.na(dataID$step))] <-
    dataID$y[which(is.na(dataID$step))] <- NA cols <- rep("#E69F00", nrow(dataID))
    cols[which(dataID$state == 2)] <- "#009E73"

plot.bi.states <- plot.bi.states + geom_path(data = dataID, aes(x = x, y = y), colour =
    cols, size = 0.2, na.rm = T, alpha = 0.2) + geom_point(data = dataID, aes(x = x, y =
    y), colour = cols, size = 0.1, na.rm = T, alpha = 0.2) }

plot.bi.states + geom_sf(data = bi_coast, fill = NA) + coord_sf(xlim = c(-38.06,
    -38.045), ylim = c(-54.014, -54.005), expand = FALSE) + labs(title = "b") +
    theme(panel.border = element_rect(colour = "black", fill = NA, size = 1))
```

b

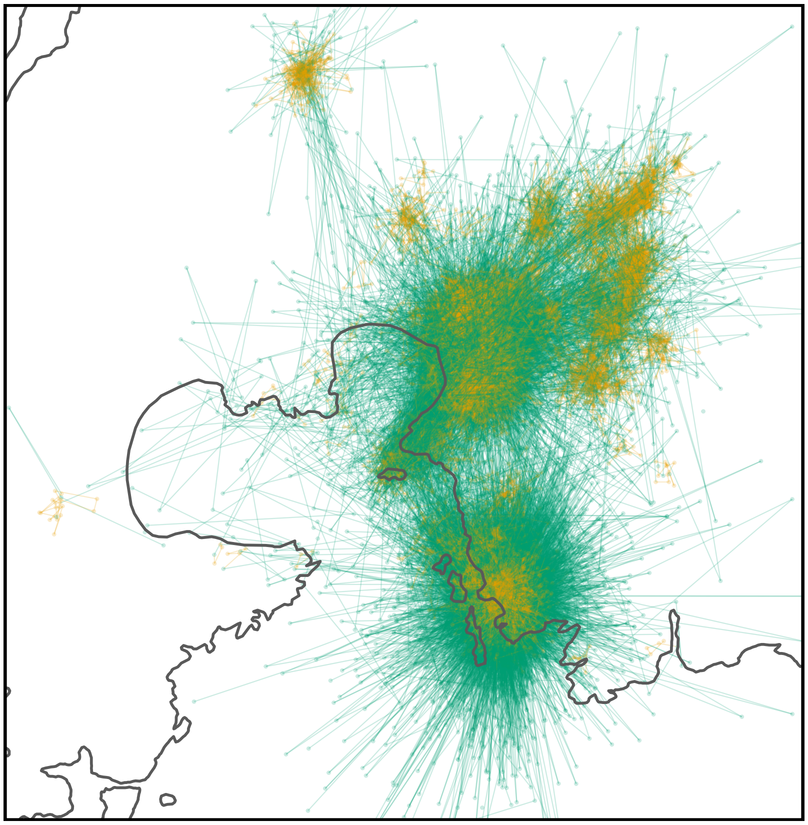

```
## Figure 2c - All recorded GPS points
ggplot() + geom_point(data = movement, aes(x = x, y = y, colour = Beach), alpha = 0.1,
    size = 0.5, inherit.aes = FALSE, na.rm = TRUE) + geom_sf(data = bi_coast, fill = NA)
    + labs(title = "c") + theme_void() + theme(legend.position = "none") +
    scale_color_manual(name = "", labels = c("FWB", "SSB"), values = c("#377eb8",
    "#e41a1c")) + coord_sf(xlim = c(-38.06, -38.045), ylim = c(-54.014, -54.005), expand
    = FALSE) + theme(panel.border = element_rect(colour = "black", fill = NA, size = 1))
```

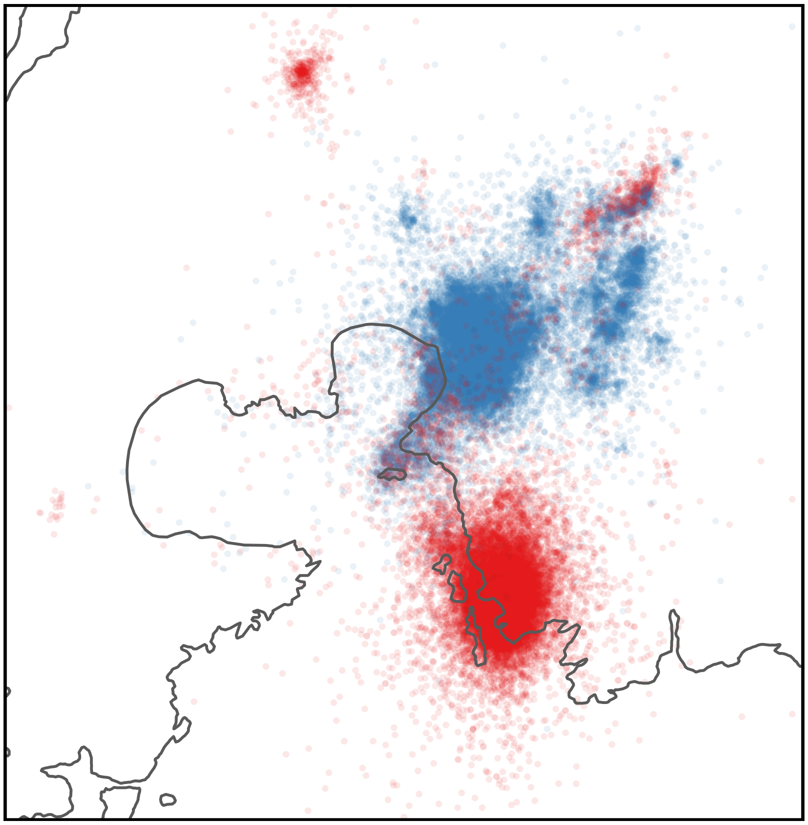

Figure 3

(a, b) The probability of being in an active state relative to time of day. (c, d) The probability of being in an active state relative to pup age. (e, f) Proportion of time spent on the colony of birth (beach habitat) vs further inland in the tussock grass.

```
## Figure 3 a & b - HMM, Time of day functions to calculate stationary
## distribution & 95% confidence intervals

draws <- 5000
betadraws <- array(NA, c(draws, nrow(mod$mle$beta), 2))
Fisher <- solve(mod$mod$hessian)
for (k in 1:draws) { betadraws[k, , ] <- matrix(rmvnorm(1, mean =
    as.vector(t(mod$mle$beta)), sigma = Fisher[5:(length(mod$mod$estimate) - 1),
    5:(length(mod$mod$estimate) - 1)]), nrow = nrow(mod$mle$beta), byrow = T) }

buildTPM <- function(beta, x, i, beach) { eta12 <- beta[1, 1] + beta[2, 1] * x[i] +
    beta[3, 1] * beach + beta[4, 1] * beach * x[i] eta21 <- beta[1, 2] + beta[2, 2] *
    x[i] + beta[3, 2] * beach + beta[4, 2] * beach * x[i]

Gamma <- matrix(c(0, eta12, eta21, 0), byrow = TRUE, nrow = 2) Gamma <- exp(Gamma) Gamma
    <- Gamma/apply(Gamma, 1, sum) return(Gamma) }

stationaries <- function(x, beta, rows, buildTPM = buildTPM) { TPM <- matrix(0, 2,
    length(x)) TPML <- matrix(0, 2, length(x)) TPMU <- matrix(0, 2, length(x)) for (i in
```

```
    1:2) { for (k in 1:length(x)) { Gamma <- buildTPM(beta, x, k, beach = i - 1)
    stationary <- solve(t(diag(2) - Gamma + matrix(1, 2, 2)), rep(1, 2)) TPM[, k] <-
    stationary stationary_draws <- matrix(NA, draws, 2) for (j in 1:draws) { betaTemp <-
    betadraws[j, rows, ] Gamma <- buildTPM(betaTemp, x, k, beach = i - 1)
    stationary_draws[j, ] <- solve(t(diag(2) - Gamma + matrix(1, 2, 2)), rep(1, 2)) }
    TPML[1, k] <- quantile(stationary_draws[, 1], 0.025) TPML[2, k] <-
    quantile(stationary_draws[, 2], 0.025) TPMU[1, k] <- quantile(stationary_draws[, 1],
    0.975) TPMU[2, k] <- quantile(stationary_draws[, 2], 0.975) } if (i == 1) {
    TPML_Beach0 <- TPML TPMU_Beach0 <- TPMU TPM_Beach0 <- TPM } } return(list(TPM, TPML,
    TPMU, TPM_Beach0, TPML_Beach0, TPMU_Beach0)) }

## prepare data
buildTPM.time <- function(beta, x, i, beach) { eta12 <- beta[1, 1] + beta[2, 1] * sin((2
    * pi * x[i])/24) + beta[3, 1] * cos((2 * pi * x[i])/24) + beta[4, 1] * beach +
    beta[5, 1] * beach * sin((2 * pi * x[i])/24) + beta[6, 1] * beach * cos((2 * pi *
    x[i])/24) eta21 <- beta[1, 2] + beta[2, 2] * sin((2 * pi * x[i])/24) + beta[3, 2] *
    cos((2 * pi * x[i])/24) + beta[4, 2] * beach + beta[5, 2] * beach * sin((2 * pi *
    x[i])/24) + beta[6, 2] * beach * cos((2 * pi * x[i])/24)

Gamma <- matrix(c(0, eta12, eta21, 0), byrow = TRUE, nrow = 2) Gamma <- exp(Gamma) Gamma
    <- Gamma/apply(Gamma, 1, sum) return(Gamma) }


rows <- which(rownames(mod$mle$beta) %in% c("intercept", "Beach01", "sin((2 * pi *
    Hour)/24)", "sin((2 * pi * Hour)/24):Beach01", "cos((2 * pi * Hour)/24)", "cos((2 *
    pi * Hour)/24):Beach01"))
beta <- mod$mle$beta[rows, ]
x <- seq(0, 24, by = 0.05)

TPMs <- stationaries(x, beta, rows, buildTPM.time)
TPM <- TPMs[[1]]
TPML <- TPMs[[2]]
TPMU <- TPMs[[3]]
TPM_Beach0 <- TPMs[[4]]
TPML_Beach0 <- TPMs[[5]]
TPMU_Beach0 <- TPMs[[6]]

timeofday.fwb <- data.frame(hour = x, prob = TPM[2, ], prob_ci_l = TPML[2, ], prob_ci_u =
    TPMU[2, ])

timeofday.ssb <- data.frame(hour = x, prob = TPM_Beach0[2, ], prob_ci_l = TPML_Beach0[2,
    ], prob_ci_u = TPMU_Beach0[2, ])

# plotting
plot.timeofday.fwb <- ggplot(data = timeofday.fwb, aes(x = hour, y = prob)) +
    geom_line(color = "#377eb8", lwd = 2) + geom_ribbon(aes(ymin = prob_ci_l, ymax =
    prob_ci_u), fill = "#377eb8", alpha = 0.3) + theme_classic() + theme(legend.position
    = "none") + scale_x_continuous(limits = c(0, 24), labels = c(0, 4, 8, 12, 16, 20,
    24), breaks = c(0, 4, 8, 12, 16, 20, 24), expand = c(0, 0)) +
    scale_y_continuous(limits = c(0, 1), expand = c(0, 0)) + labs(x = "Time of day
    (hours)", y = "Probability", title = "a")
```

```r
plot.timeofday.ssb <- ggplot(data = timeofday.ssb, aes(x = hour, y = prob)) +
    geom_line(color = "#e41a1c", lwd = 2) + geom_ribbon(aes(ymin = prob_ci_l, ymax =
    prob_ci_u), fill = "#e41a1c", alpha = 0.3) + theme_classic() + theme(legend.position
    = "none") + scale_x_continuous(limits = c(0, 24), labels = c(0, 4, 8, 12, 16, 20,
    24), breaks = c(0, 4, 8, 12, 16, 20, 24), expand = c(0, 0)) +
    scale_y_continuous(limits = c(0, 1), expand = c(0, 0)) + labs(x = "Time of day
    (hours)", y = "", title = "b")

## Figure 3 c & d - HMM, age prepare data
buildTPM.age <- function(beta, x, i, beach) { eta12 <- beta[1, 1] + beta[2, 1] * x[i] +
    beta[3, 1] * x[i]^2 + beta[4, 1] * beach + beta[5, 1] * beach * x[i] + beta[6, 1] *
    beach * x[i]^2 eta21 <- beta[1, 2] + beta[2, 2] * x[i] + beta[3, 2] * x[i]^2 +
    beta[4, 2] * beach + beta[5, 2] * beach * x[i] + beta[6, 2] * beach * x[i]^2

Gamma <- matrix(c(0, eta12, eta21, 0), byrow = TRUE, nrow = 2) Gamma <- exp(Gamma) Gamma
    <- Gamma/apply(Gamma, 1, sum) return(Gamma) }

rows <- which(rownames(mod$mle$beta) %in% c("intercept", "Beach01", "Age_sd",
    "Age_sd:Beach01", "I(Age_sd^2)", "I(Age_sd^2):Beach01"))
beta <- mod$mle$beta[rows, ]
x <- seq(min(allData$Age_sd), max(allData$Age_sd), by = 0.02)

TPMs <- stationaries(x, beta, rows, buildTPM.age)
TPM <- TPMs[[1]]
TPML <- TPMs[[2]]
TPMU <- TPMs[[3]]
TPM_Beach0 <- TPMs[[4]]
TPML_Beach0 <- TPMs[[5]]
TPMU_Beach0 <- TPMs[[6]]

age.fwb <- data.frame(day = x, prob = TPM[2, ], prob_ci_l = TPML[2, ], prob_ci_u =
    TPMU[2, ])
max(allData$Age[allData$Beach == "FWB"])
```

```
## [1] 67
```

```r
age.ssb <- data.frame(day = x, prob = TPM_Beach0[2, ], prob_ci_l = TPML_Beach0[2, ],
    prob_ci_u = TPMU_Beach0[2, ])
max(allData$Age[allData$Beach == "SSB"])
```

```
## [1] 80
```

```r
as.table((seq(0, 80) - mean(allData$Age))/sd(allData$Age))[80]
```

```
##       B3
## 3.157454
```

```r
# plotting
plot.age.fwb <- ggplot(data = age.fwb, aes(x = day, y = prob)) + geom_line(color =
    "#377eb8", lwd = 2) + geom_ribbon(aes(ymin = prob_ci_l, ymax = prob_ci_u), fill =
    "#377eb8", alpha = 0.3) + theme_classic() + theme(plot.subtitle = element_markdown(),
```

```
    legend.position = "none") + coord_cartesian(xlim = c(as.table((seq(0, 80) -
    mean(allData$Age))/sd(allData$Age))[1], as.table((seq(0, 80) -
    mean(allData$Age))/sd(allData$Age))[67])) + scale_x_continuous(breaks = (seq(0, 80,
    by = 10) - mean(allData$Age))/sd(allData$Age), labels = seq(0, 80, by = 10), expand =
    c(0, 0)) + scale_y_continuous(limits = c(0, 1), expand = c(0, 0)) + labs(x = "Age
    (days)", y = "Probability", title = "c")

plot.age.ssb <- ggplot(data = age.ssb, aes(x = day, y = prob)) + geom_line(color =
    "#e41a1c", lwd = 2) + geom_ribbon(aes(ymin = prob_ci_l, ymax = prob_ci_u), fill =
    "#e41a1c", alpha = 0.3) + theme_classic() + theme(plot.subtitle = element_markdown(),
    legend.position = "none") + coord_cartesian(xlim = c(as.table((seq(0, 80) -
    mean(allData$Age))/sd(allData$Age))[1], as.table((seq(0, 80) -
    mean(allData$Age))/sd(allData$Age))[80])) + scale_x_continuous(breaks = (seq(0, 90,
    by = 10) - mean(allData$Age))/sd(allData$Age), labels = seq(0, 90, by = 10), expand =
    c(0, 0)) + scale_y_continuous(limits = c(0, 1), expand = c(0, 0)) + labs(x = "Age
    (days)", y = "", title = "d")

## Figure 3 e & f - Beach vs tussock grass prepare data

# remove any rows with missing coordinates
movement_rmNA <- subset(movement, !is.na(x))
# buffer the beaches = 50 meters
fwb_buff = st_buffer(fwb, 5e-04)
ssb_buff = st_buffer(ssb, 5e-04)
# retrieve coordinates from shapefiles
ssb_buff_cord <- data.frame(st_coordinates(ssb_buff$geometry))
fwb_buff_cord <- data.frame(st_coordinates(fwb_buff$geometry))

# split FWB and SSB into two seperate files
movement_ssb_buff <- movement_rmNA[movement_rmNA$Beach == "SSB", ]
movement_fwb_buff <- movement_rmNA[movement_rmNA$Beach == "FWB", ]

## do points (movement) fall in a given polygon (fwb or ssb)?
ssb_buff_yn <- data.frame(ssb_buff_yn = point.in.polygon(movement_ssb_buff$x,
    movement_ssb_buff$y, ssb_buff_cord$X, ssb_buff_cord$Y, mode.checked = FALSE))

fwb_buff_yn <- data.frame(fwb_buff_yn = point.in.polygon(movement_fwb_buff$x,
    movement_fwb_buff$y, fwb_buff_cord$X, fwb_buff_cord$Y, mode.checked = FALSE))

# merge
movement_ssb_buff <- cbind(movement_ssb_buff, ssb_buff_yn)
movement_fwb_buff <- cbind(movement_fwb_buff, fwb_buff_yn)

## plotting
plot_fwb_buff_per <- ggplot() + geom_bar(aes(x = as.factor(Age), fill =
    as.factor(fwb_buff_yn)), data = movement_fwb_buff, position = "fill") +
    scale_fill_manual(name = "", labels = c("", "FWB"), values = c(NA, "#377eb8")) +
    scale_y_continuous(expand = c(0, 0)) + scale_x_discrete(breaks = seq(0, 80, by = 10),
    labels = seq(0, 80, by = 10), expand = c(0, 0)) + theme_classic() +
    theme(legend.position = "bottom") + guides(fill = guide_legend(label.position =
    "left", label.hjust = 1)) + labs(y = "Count", x = "Age (days)", title = "e")

plot_ssb_buff_per <- ggplot() + geom_bar(aes(x = as.factor(Age), fill =
```

```r
    as.factor(ssb_buff_yn)), data = movement_ssb_buff, position = "fill") +
    scale_fill_manual(name = "", labels = c("", "SSB"), values = c(NA, "#e41a1c"), guide
    = guide_legend(reverse = TRUE)) + scale_y_continuous(expand = c(0, 0)) +
    scale_x_discrete(breaks = seq(0, 70, by = 10), labels = seq(0, 70, by = 10), expand =
    c(0, 0)) + theme_classic() + theme(legend.position = "bottom") + labs(y = "", x =
    "Age (days)", title = "f")

## Figure 3 - combined
(plot.timeofday.fwb + plot.timeofday.ssb)/(plot.age.fwb +
    plot.age.ssb)/(plot_fwb_buff_per + plot_ssb_buff_per)/guide_area() +
    plot_layout(heights = c(1, 1, 1, 0.1), guides = "collect") & theme(legend.position =
    "bottom", text = element_text(size = 14))
```

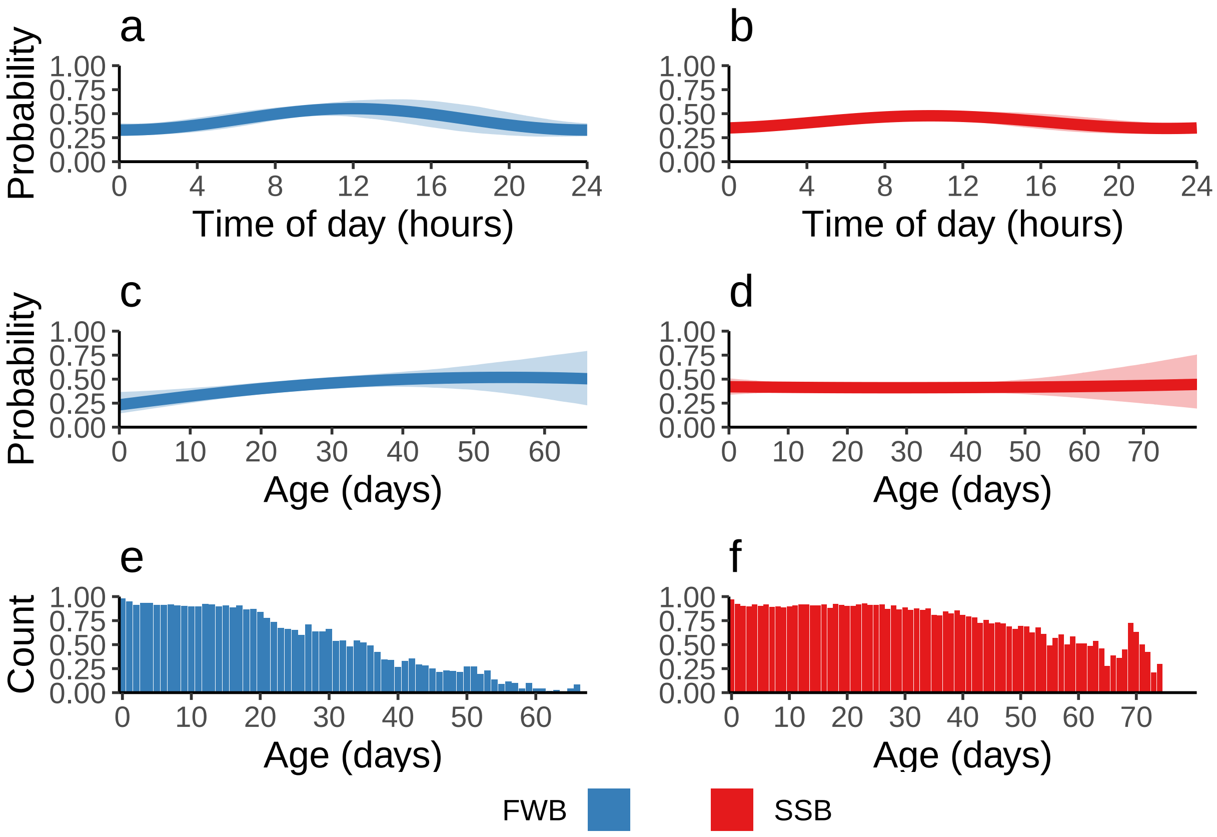

Figure 4

Results of post-hoc analyses. (a) Pup activity given maternal absence/ present on the island. (b) Pup activity given survival status.

```r
## Figure 4 - Post hoc, Death
plot_death <- ggplot(per.act.death, aes(death, weight = value)) + geom_bar(aes(fill =
    state), position = "fill") + geom_text(aes(label = per, y = c(0.75, 0.75, 0.2, 0.2)))
    + scale_y_continuous(expand = c(0, 0)) + scale_x_discrete(limits = c("Survived",
    "Mortality"), labels = c("Pups that\nsurvived", "Pups that\ndied")) +
    scale_fill_manual(name = NULL, labels = c("Inactive state", "Active state"), values =
```

```r
    c("#E69F00", "#009E73")) + labs(y = "", x = "", title = "b", subtitle =
    expression(paste(italic("n"), "= 66, ", "estimate = -0.758, ", italic("p"), " <
    0.001"))) + theme_classic()

## Figure 4 - Post hoc, Attendance
plot_mum.ashore <- ggplot(per.act.ashore, aes(attendance, weight = value)) +
    geom_bar(aes(fill = state), position = "fill") + geom_text(aes(label = per, y =
    c(0.75, 0.75, 0.2, 0.2))) + scale_y_continuous(expand = c(0, 0)) +
    scale_x_discrete(limits = c("Foraging", "Ashore"), labels = c("Mothers \nat sea",
    "Mothers \nashore")) + scale_fill_manual(name = NULL, labels = c("Inactive state",
    "Active state"), values = c("#E69F00", "#009E73")) + labs(y = "Proportion", x = "",
    title = "a", subtitle = expression(paste(italic("n"), " = 24, ", "estimate = -0.091,
    ", italic("p"), " = 0.032"))) + theme_classic()

## Figure 4 - combined
plot_mum.ashore + plot_death + plot_layout(guides = "collect") & theme(text =
    element_text(size = 14), plot.subtitle = element_text(size = 12)) & guides(color =
    guide_legend(override.aes = list(pch = 15)))
```

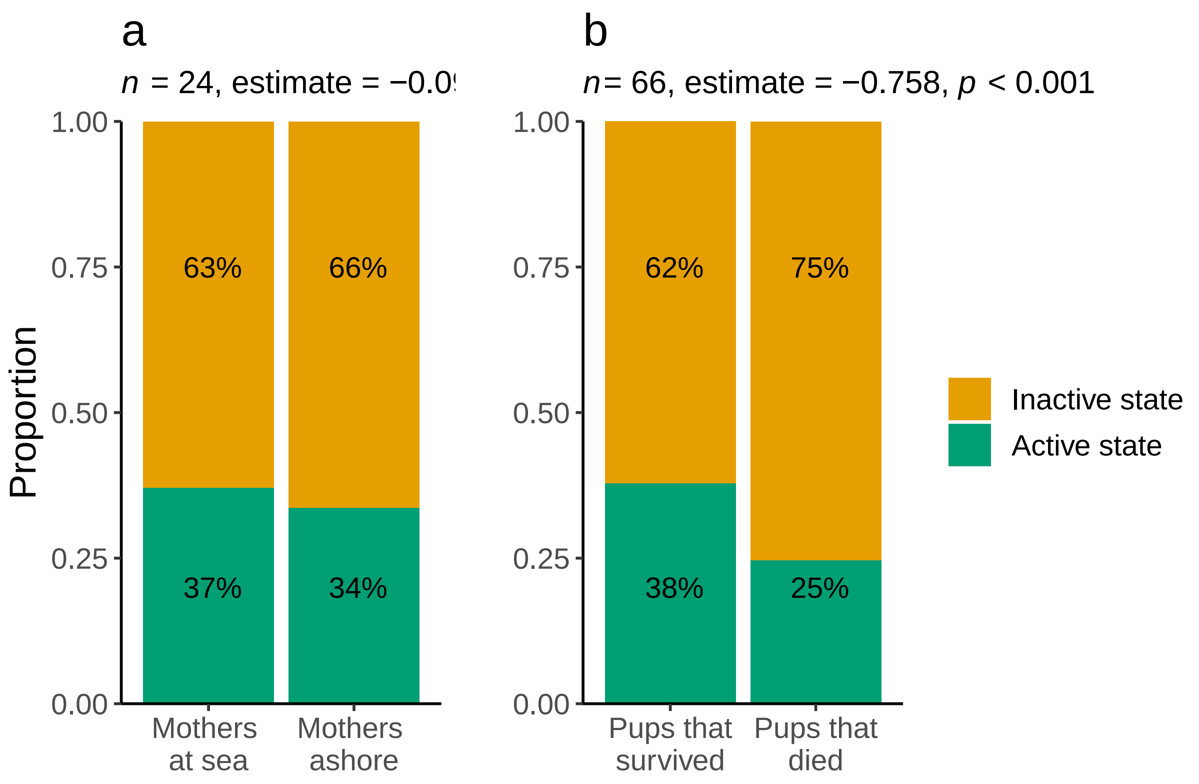

Manuscript Figures - Supplementary information

Figure S1

Map of Bird Island, South Georgia, showing all recorded GPS positions and states.

```
## Figure S1 - map of the entire island showing all recorded positions and states
plot.bi.states + geom_sf(data = bi_coast, fill = NA) + coord_sf() + scalebar(data =
    bi_coast, dist = 500, dist_unit = "m", transform = TRUE, model = "WGS84", location =
    "bottomright", box.fill = "white", border.size = 0.5) +
    annotation_north_arrow(location = "tl", which_north = "true", style =
    north_arrow_fancy_orienteering)
```

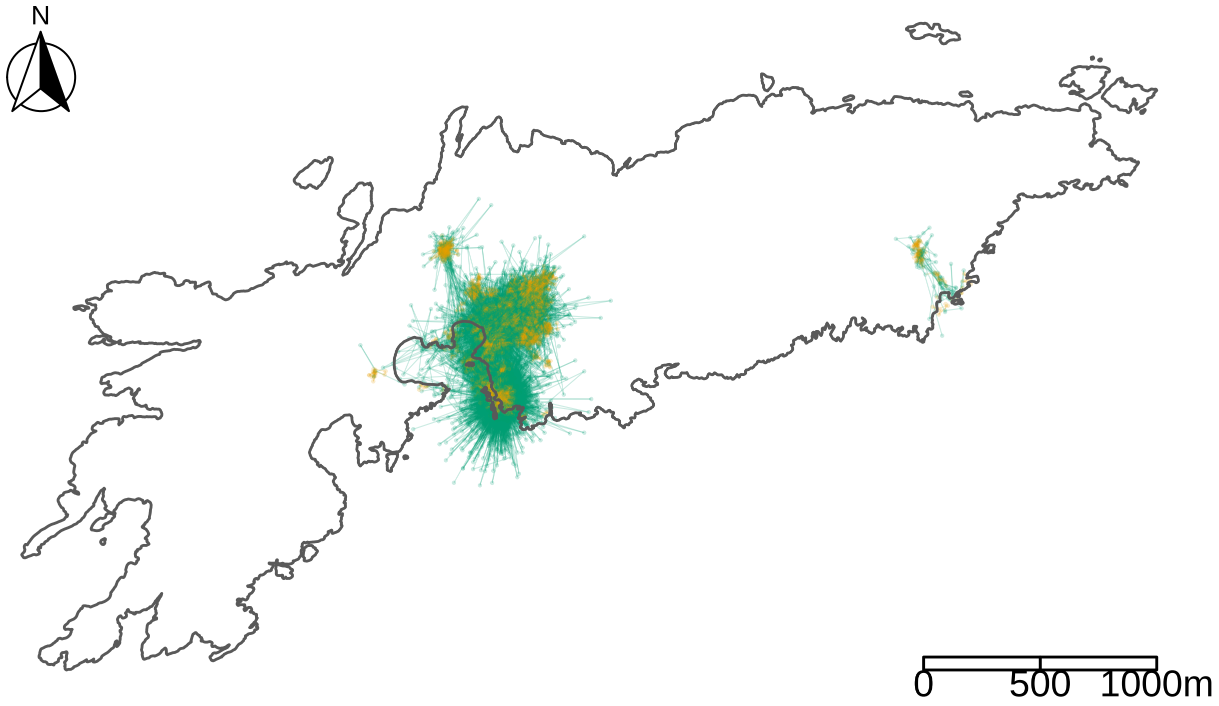

Figure S2

Representative examples of individual GPS tracks, including one pup from each breading colony and season.

```
## Figure S2 - representative examples of individual tracks C11
c11 <- ggplot() + geom_point(data = movement[movement$IDno == "C11", ], aes(x = x, y = y,
    colour = state), alpha = 0.1, size = 0.2, inherit.aes = FALSE, na.rm = TRUE) +
    geom_path(data = movement[movement$IDno == "C11", ], aes(x = x, y = y, colour =
    state), na.rm = TRUE, size = 0.2, alpha = 0.5) + geom_sf(data = bi_coast, fill = NA)
    + labs(title = "Individual C11 - FWB 2019") + theme_void() + theme(legend.position =
    "none") + scale_color_manual(name = "", labels = c("1", "2"), values = c("#E69F00",
```

```
    "#009E73")) + coord_sf(xlim = c(-38.06, -38.045), ylim = c(-54.0151, -54.0061),
    expand = FALSE) + theme(panel.border = element_rect(colour = "black", fill = NA, size
    = 1), plot.margin = margin(5, 5, 5, 5, "mm"))

# T1
t1 <- ggplot() + geom_point(data = movement[movement$IDno == "T1", ], aes(x = x, y = y,
    colour = state), alpha = 0.1, size = 0.2, inherit.aes = FALSE, na.rm = TRUE) +
    geom_path(data = movement[movement$IDno == "T1", ], aes(x = x, y = y, colour =
    state), na.rm = TRUE, size = 0.2, alpha = 0.5) + geom_sf(data = bi_coast, fill = NA)
    + labs(title = "Individual T1 - SSB 2020") + theme_void() + theme(legend.position =
    "none") + scale_color_manual(name = "", labels = c("1", "2"), values = c("#E69F00",
    "#009E73")) + coord_sf(xlim = c(-38.06, -38.045), ylim = c(-54.0151, -54.0061),
    expand = FALSE) + theme(panel.border = element_rect(colour = "black", fill = NA, size
    = 1), plot.margin = margin(5, 5, 5, 5, "mm"))

# N4
n4 <- ggplot() + geom_point(data = movement[movement$IDno == "N4", ], aes(x = x, y = y,
    colour = state), alpha = 0.1, size = 0.2, inherit.aes = FALSE, na.rm = TRUE) +
    geom_path(data = movement[movement$IDno == "N4", ], aes(x = x, y = y, colour =
    state), na.rm = TRUE, size = 0.2, alpha = 0.4) + geom_sf(data = bi_coast, fill = NA)
    + labs(title = "Individual N4 - FWB 2020") + theme_void() + theme(legend.position =
    "none") + scale_color_manual(name = "", labels = c("1", "2"), values = c("#E69F00",
    "#009E73")) + coord_sf(xlim = c(-38.06, -38.045), ylim = c(-54.0151, -54.0061),
    expand = FALSE) + theme(panel.border = element_rect(colour = "black", fill = NA, size
    = 1), plot.margin = margin(5, 5, 5, 5, "mm"))

# H19
h19 <- ggplot() + geom_point(data = movement[movement$IDno == "h19", ], aes(x = x, y = y,
    colour = state), alpha = 0.1, size = 0.2, inherit.aes = FALSE, na.rm = TRUE) +
    geom_path(data = movement[movement$IDno == "H19", ], aes(x = x, y = y, colour =
    state), na.rm = TRUE, size = 0.2, alpha = 0.4) + geom_sf(data = bi_coast, fill = NA)
    + labs(title = "Individual H19 - SSB 2019") + theme_void() + theme(legend.position =
    "none") + scale_color_manual(name = "", labels = c("1", "2"), values = c("#E69F00",
    "#009E73")) + coord_sf(xlim = c(-38.06, -38.045), ylim = c(-54.0151, -54.0061),
    expand = FALSE) + theme(panel.border = element_rect(colour = "black", fill = NA, size
    = 1), plot.margin = margin(5, 5, 5, 5, "mm"))

# legend

legend <- data.frame(x = c(1, 1, 1, 1), y = c(1, 2, 3, 4), label = c("FWB", "SSB", "state
    1", "state 2"))

plot.legend.si <- ggplot(legend[3:4, ]) + geom_point(aes(x = x, y = y, col = label)) +
    scale_color_manual(name = "", label = c("Inactive state", "Active state"), values =
    c("#E69F00", "#009E73")) + guides(color = guide_legend(override.aes = list(size = 6,
    pch = 15), label.position = "left", label.hjust = 1)) + theme(legend.position =
    "bottom", text = element_text(size = 12), legend.key = element_rect(fill = NA),
    legend.title = element_blank(), legend.margin = margin(c(0, 0, 10, 30)))

my_legend_si <- get_legend(plot.legend.si)

# Figure S2 - combined
(h19 + t1)/(c11 + n4)/(plot_spacer() + as_ggplot(my_legend_si) + plot_spacer()) +
```

```
    plot_layout(heights = c(2, 2, 0.25))
```

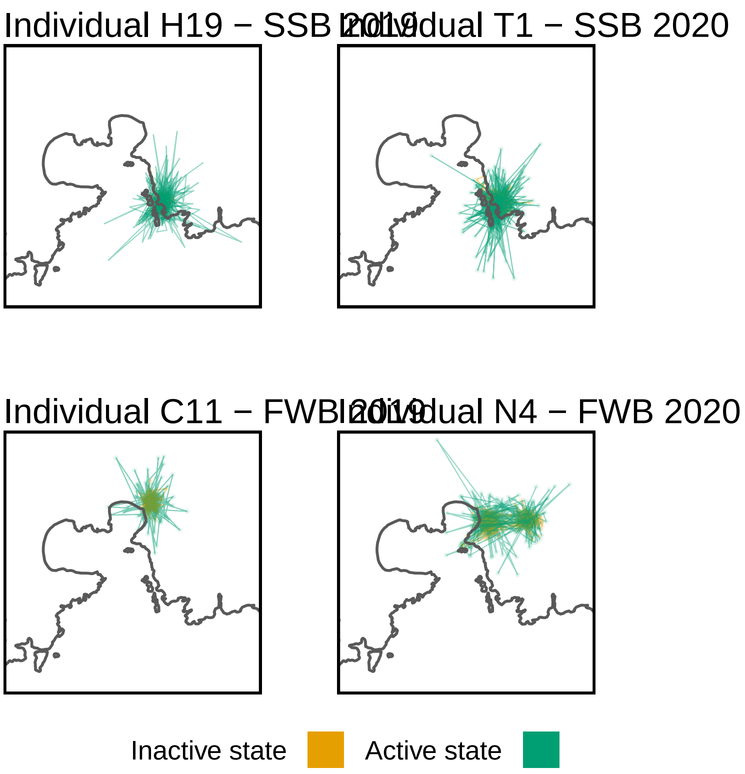

Figure S4

HMMs fitted to 5-minute interval GPS data collected from three fur seal pups, each between 30 – 50 days of age, for 13 – 19 days (mean = 3,965 data points).

```
## Figure S5 - 5 minute intervals

n21 <- ggplot() + geom_point(data = highFreq[highFreq$ID == "N21", ], aes(x = x, y = y,
    colour = as.factor(states2)), alpha = 0.1, size = 0.2, inherit.aes = FALSE, na.rm =
    TRUE) + geom_path(data = highFreq[highFreq$ID == "N21", ], aes(x = x, y = y, colour =
    as.factor(states2)), na.rm = TRUE, size = 0.2, alpha = 0.4) + geom_sf(data =
    bi_coast, fill = NA) + labs(title = "Individual N21\nFWB 2020") + theme_void() +
    theme(legend.position = "none") + scale_color_manual(name = "", labels = c("1", "2"),
    values = c("#E69F00", "#56B4E9")) + coord_sf(xlim = c(-38.064, -38.045), ylim =
    c(-54.019, -54.005), expand = FALSE) + theme(panel.border = element_rect(colour =
    "black", fill = NA, size = 1), plot.margin = margin(0, 2, 0, 2, "mm"))

t23 <- ggplot() + geom_point(data = highFreq[highFreq$ID == "T23", ], aes(x = x, y = y,
    colour = as.factor(states2)), alpha = 0.1, size = 0.2, inherit.aes = FALSE, na.rm =
    TRUE) + geom_path(data = highFreq[highFreq$ID == "T23", ], aes(x = x, y = y, colour =
    as.factor(states2)), na.rm = TRUE, size = 0.2, alpha = 0.4) + geom_sf(data =
    bi_coast, fill = NA) + labs(title = "Individual T23\nSSB 2020") + theme_void() +
```

```
    theme(legend.position = "none") + scale_color_manual(name = "", labels = c("1", "2"),
    values = c("#E69F00", "#56B4E9")) + coord_sf(xlim = c(-38.06, -38.043), ylim =
    c(-54.0151, -54.0061), expand = FALSE) + theme(panel.border = element_rect(colour =
    "black", fill = NA, size = 1), plot.margin = margin(0, 2, 0, 2, "mm"))

t5 <- ggplot() + geom_point(data = highFreq[highFreq$ID == "T5", ], aes(x = x, y = y,
    colour = as.factor(states2)), alpha = 0.1, size = 0.2, inherit.aes = FALSE, na.rm =
    TRUE) + geom_path(data = highFreq[highFreq$ID == "T5", ], aes(x = x, y = y, colour =
    as.factor(states2)), na.rm = TRUE, size = 0.2, alpha = 0.4) + geom_sf(data =
    bi_coast, fill = NA) + labs(title = "Individual T5\nSSB 2020") + theme_void() +
    theme(legend.position = "none") + scale_color_manual(name = "", labels = c("1", "2"),
    values = c("#E69F00", "#56B4E9")) + coord_sf(xlim = c(-38.06, -38.043), ylim =
    c(-54.0151, -54.0061), expand = FALSE) + theme(panel.border = element_rect(colour =
    "black", fill = NA, size = 1), plot.margin = margin(0, 2, 0, 2, "mm"))

## Figure S5 - combined
n21 + t5 + t23
```

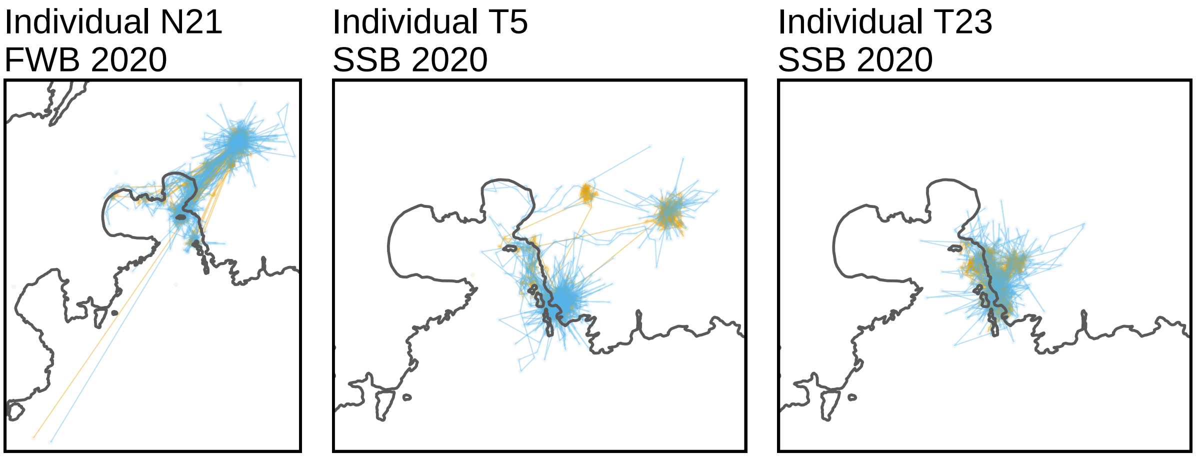

Figure S5

Five-minute interval GPS data from three pups. Randomly selected examples are shown where a pup was inferred to have moved at least 400 meters in the space of one hour. All of these tracks reveal fairly consistent step sizes across the observation time window rather than a single, erroneously large step. Consequently, we believe that 400 meters is an appropriate step threshold for the HMM analysis.

```r
# random examples of step length in an hour < 400 meters
sum(highFreq[888:899, ]$step) # N21
```

```
## [1] 411.736
```

```r
mean(highFreq[888:899, ]$step)
```

```
## [1] 34.31133
```

```r
range(highFreq[888:899, ]$step)
```

```
## [1]  3.439977 71.612957
```

```r
sum(highFreq[8269:8280, ]$step) # T23
```

```
## [1] 413.7598
```

```r
mean(highFreq[8269:8280, ]$step)
```

```
## [1] 34.47998
```

```r
range(highFreq[8269:8280, ]$step)
```

```
## [1]  6.871201 69.418879
```

```r
sum(highFreq[11334:11345, ]$step) # T5
```

```
## [1] 533.8173
```

```r
mean(highFreq[11334:11345, ]$step)
```

```
## [1] 44.48477
```

```r
range(highFreq[11334:11345, ]$step)
```

```
## [1]   7.252158 175.234997
```

```r
# plotting
plot.400m <- ggplot() + geom_point(data = highFreq[888:899, ], aes(x = x, y = y), color =
    "#1b9e77", pch = 16, size = 1, inherit.aes = FALSE, na.rm = TRUE) + geom_point(data =
    highFreq[8269:8280, ], aes(x = x, y = y), color = "#d95f02", pch = 16, size = 1,
    inherit.aes = FALSE, na.rm = TRUE) + geom_point(data = highFreq[11334:11345, ], aes(x
    = x, y = y), color = "#7570b3", pch = 16, size = 1, inherit.aes = FALSE, na.rm =
    TRUE) + geom_sf(data = bi_coast, fill = NA) + theme_void() + theme(legend.position =
    "none") + coord_sf(xlim = c(-38.06, -38.043), ylim = c(-54.0151, -54.005), expand =
    FALSE) + theme(panel.border = element_rect(colour = "black", fill = NA, size = 1),
```

```r
    plot.margin = margin(0, 2, 0, 2, "mm"))

plot.400m.N21zoom <- ggplot() + geom_point(data = highFreq[888:899, ], aes(x = x, y = y),
    color = "#1b9e77", pch = 16, size = 1, inherit.aes = FALSE, na.rm = TRUE) +
    geom_line(data = highFreq[888:899, ], aes(x = x, y = y), color = "#1b9e77", size = 1,
    inherit.aes = FALSE, na.rm = TRUE) + geom_sf(data = bi_coast, fill = NA) + theme_bw()
    + theme(legend.position = "none") + coord_sf(xlim = c(-38.0497, -38.0483), ylim =
    c(-54.0078, -54.0071), expand = FALSE) + scale_y_continuous(breaks = c(-54.0071)) +
    scale_x_continuous(breaks = c(-38.0483)) + labs(x = "", y = "", title = "N21",
    subtitle = "411 meters traveled in an hour")

plot.400m.T23zoom <- ggplot() + geom_point(data = highFreq[8269:8280, ], aes(x = x, y =
    y), color = "#d95f02", pch = 16, size = 1, inherit.aes = FALSE, na.rm = TRUE) +
    geom_line(data = highFreq[8269:8280, ], aes(x = x, y = y), color = "#d95f02", size =
    1, inherit.aes = FALSE, na.rm = TRUE) + geom_sf(data = bi_coast, fill = NA) +
    theme_bw() + theme(legend.position = "none") + coord_sf(xlim = c(-38.05095,
    -38.0496), ylim = c(-54.0109, -54.0103), expand = FALSE) + scale_y_continuous(breaks
    = c(-54.0103)) + scale_x_continuous(breaks = c(-38.0496)) + labs(x = "", y = "",
    title = "T23", subtitle = "413 meters traveled in an hour")

plot.400m.T5zoom <- ggplot() + geom_point(data = highFreq[11334:11345, ], aes(x = x, y =
    y), color = "#7570b3", pch = 19, size = 1, inherit.aes = FALSE, na.rm = TRUE) +
    geom_line(data = highFreq[11334:11345, ], aes(x = x, y = y), color = "#7570b3", size
    = 1, inherit.aes = FALSE, na.rm = TRUE) + geom_sf(data = bi_coast, fill = NA, color =
    NA) + theme_bw() + theme(legend.position = "none") + coord_sf(xlim = c(-38.0514,
    -38.0498), ylim = c(-54.0117, -54.011), expand = FALSE) + scale_y_continuous(breaks =
    c(-54.011)) + scale_x_continuous(breaks = c(-38.0498)) + labs(x = "", y = "", title =
    "T5", subtitle = "533 meters traveled in an hour")

layout <- c(patchwork::area(t = 1, l = 1, b = 3, r = 2), patchwork::area(t = 1, l = 3, b
    = 1, r = 4), patchwork::area(t = 2, l = 3, b = 2, r = 4), patchwork::area(t = 3, l =
    3, b = 3, r = 4))

plot.400m + plot.400m.N21zoom + plot.400m.T23zoom + plot.400m.T5zoom + plot_layout(design
    = layout) & theme(text = element_text(size = 14))
```

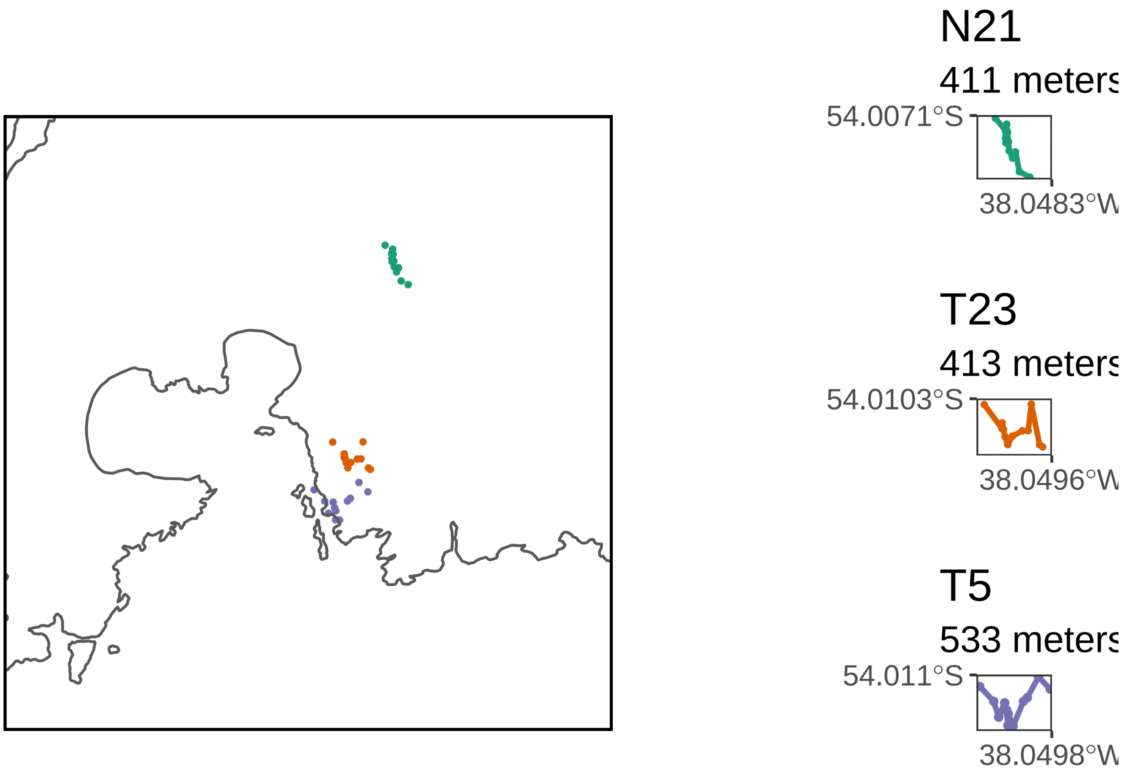

***

```
## - Session info ---------------------------------------------------------------
##  setting  value
##  version  R version 4.0.2 (2020-06-22)
##  os       Windows 10 x64
##  system   x86_64, mingw32
##  ui       RTerm
##  language (EN)
##  collate  English_United States.1252
##  ctype    English_United States.1252
##  tz       Europe/Berlin
##  date     2021-02-10
##
## - Packages -------------------------------------------------------------------
##  package      * version    date       lib source
##  abind          1.4-5      2016-07-21 [1] CRAN (R 4.0.0)
##  assertthat     0.2.1      2019-03-21 [1] CRAN (R 4.0.2)
##  backports      1.1.10     2020-09-15 [1] CRAN (R 4.0.2)
##  bayestestR     0.7.2      2020-07-20 [1] CRAN (R 4.0.2)
##  bitops         1.0-6      2013-08-17 [1] CRAN (R 4.0.0)
##  boot         * 1.3-25     2020-04-26 [1] CRAN (R 4.0.3)
##  broom          0.7.0      2020-07-09 [1] CRAN (R 4.0.2)
##  car            3.0-9      2020-08-11 [1] CRAN (R 4.0.2)
##  carData        3.0-4      2020-05-22 [1] CRAN (R 4.0.0)
##  cellranger     1.1.0      2016-07-27 [1] CRAN (R 4.0.2)
##  chron        * 2.3-56     2020-08-18 [1] CRAN (R 4.0.2)
```

```
##  CircStats    * 0.2-6      2018-07-01 [1] CRAN (R 4.0.3)
##  class          7.3-17     2020-04-26 [2] CRAN (R 4.0.2)
##  classInt       0.4-3      2020-04-07 [1] CRAN (R 4.0.2)
##  cli            2.0.2      2020-02-28 [1] CRAN (R 4.0.2)
##  coda           0.19-3     2019-07-05 [1] CRAN (R 4.0.2)
##  codetools      0.2-16     2018-12-24 [2] CRAN (R 4.0.2)
##  colorspace     1.4-1      2019-03-18 [1] CRAN (R 4.0.2)
##  cowplot        1.1.0      2020-09-08 [1] CRAN (R 4.0.2)
##  crayon         1.3.4      2017-09-16 [1] CRAN (R 4.0.2)
##  curl           4.3        2019-12-02 [1] CRAN (R 4.0.2)
##  data.table   * 1.13.0     2020-07-24 [1] CRAN (R 4.0.2)
##  DBI            1.1.0      2019-12-15 [1] CRAN (R 4.0.2)
##  DHARMa       * 0.3.3.0    2020-09-08 [1] CRAN (R 4.0.2)
##  digest         0.6.25     2020-02-23 [1] CRAN (R 4.0.2)
##  dplyr        * 1.0.2      2020-08-18 [1] CRAN (R 4.0.2)
##  e1071          1.7-3      2019-11-26 [1] CRAN (R 4.0.2)
##  effectsize     0.3.2      2020-07-27 [1] CRAN (R 4.0.2)
##  ellipsis       0.3.1      2020-05-15 [1] CRAN (R 4.0.2)
##  emmeans        1.5.0      2020-08-18 [1] CRAN (R 4.0.2)
##  estimability   1.3        2018-02-11 [1] CRAN (R 4.0.0)
##  evaluate       0.14       2019-05-28 [1] CRAN (R 4.0.2)
##  fansi          0.4.1      2020-01-08 [1] CRAN (R 4.0.2)
##  farver         2.0.3      2020-01-16 [1] CRAN (R 4.0.2)
##  forcats        0.5.0      2020-03-01 [1] CRAN (R 4.0.2)
##  foreach        1.5.0      2020-03-30 [1] CRAN (R 4.0.2)
##  foreign        0.8-80     2020-05-24 [2] CRAN (R 4.0.2)
##  formatR      * 1.7        2019-06-11 [1] CRAN (R 4.0.2)
##  generics       0.0.2      2018-11-29 [1] CRAN (R 4.0.2)
##  geosphere      1.5-10     2019-05-26 [1] CRAN (R 4.0.3)
##  ggeffects      0.16.0     2020-09-13 [1] CRAN (R 4.0.2)
##  ggmap          3.0.0      2019-02-04 [1] CRAN (R 4.0.2)
##  ggplot2      * 3.3.2      2020-06-19 [1] CRAN (R 4.0.2)
##  ggpubr       * 0.4.0      2020-06-27 [1] CRAN (R 4.0.2)
##  ggsignif       0.6.0      2019-08-08 [1] CRAN (R 4.0.2)
##  ggspatial    * 1.1.4      2020-07-12 [1] CRAN (R 4.0.3)
##  ggtext       * 0.1.0.9000 2020-11-25 [1] Github (clauswilke/ggtext@dfb02af)
##  glue           1.4.2      2020-08-27 [1] CRAN (R 4.0.2)
##  gridExtra    * 2.3        2017-09-09 [1] CRAN (R 4.0.2)
##  gridtext       0.1.1      2020-02-24 [1] CRAN (R 4.0.3)
##  gtable         0.3.0      2019-03-25 [1] CRAN (R 4.0.2)
##  haven          2.3.1      2020-06-01 [1] CRAN (R 4.0.2)
##  hms            0.5.3      2020-01-08 [1] CRAN (R 4.0.2)
##  htmltools      0.5.0      2020-06-16 [1] CRAN (R 4.0.2)
##  httr           1.4.2      2020-07-20 [1] CRAN (R 4.0.2)
##  insight        0.9.5      2020-09-07 [1] CRAN (R 4.0.2)
##  iterators      1.0.12     2019-07-26 [1] CRAN (R 4.0.2)
##  jpeg           0.1-8.1    2019-10-24 [1] CRAN (R 4.0.0)
##  kableExtra   * 1.2.1      2020-08-27 [1] CRAN (R 4.0.2)
##  KernSmooth     2.23-17    2020-04-26 [2] CRAN (R 4.0.2)
##  knitr        * 1.29       2020-06-23 [1] CRAN (R 4.0.2)
##  labeling       0.3        2014-08-23 [1] CRAN (R 4.0.0)
##  lattice        0.20-41    2020-04-02 [2] CRAN (R 4.0.2)
##  lifecycle      0.2.0      2020-03-06 [1] CRAN (R 4.0.2)
##  lme4         * 1.1-23     2020-04-07 [1] CRAN (R 4.0.2)
```

```
##  magick       * 2.5.2      2020-11-10 [1] CRAN (R 4.0.3)
##  magrittr       1.5        2014-11-22 [1] CRAN (R 4.0.2)
##  MASS         * 7.3-52     2020-08-18 [2] CRAN (R 4.0.2)
##  Matrix       * 1.2-18     2019-11-27 [2] CRAN (R 4.0.2)
##  minqa          1.2.4      2014-10-09 [1] CRAN (R 4.0.2)
##  modelr         0.1.8      2020-05-19 [1] CRAN (R 4.0.2)
##  moveHMM      * 1.7        2019-05-19 [1] CRAN (R 4.0.3)
##  multcomp       1.4-13     2020-04-08 [1] CRAN (R 4.0.2)
##  munsell        0.5.0      2018-06-12 [1] CRAN (R 4.0.2)
##  mvtnorm      * 1.1-1      2020-06-09 [1] CRAN (R 4.0.0)
##  nlme           3.1-149    2020-08-23 [2] CRAN (R 4.0.2)
##  nloptr         1.2.2.2    2020-07-02 [1] CRAN (R 4.0.2)
##  numDeriv       2016.8-1.1 2019-06-06 [1] CRAN (R 4.0.0)
##  openxlsx       4.1.5      2020-05-06 [1] CRAN (R 4.0.2)
##  parameters     0.8.5      2020-09-12 [1] CRAN (R 4.0.2)
##  patchwork    * 1.1.0      2020-11-09 [1] CRAN (R 4.0.3)
##  performance    0.5.0      2020-09-12 [1] CRAN (R 4.0.2)
##  pillar         1.4.6      2020-07-10 [1] CRAN (R 4.0.2)
##  pkgconfig      2.0.3      2019-09-22 [1] CRAN (R 4.0.2)
##  plyr           1.8.6      2020-03-03 [1] CRAN (R 4.0.2)
##  png            0.1-7      2013-12-03 [1] CRAN (R 4.0.0)
##  purrr          0.3.4      2020-04-17 [1] CRAN (R 4.0.2)
##  R6             2.4.1      2019-11-12 [1] CRAN (R 4.0.2)
##  Rcpp           1.0.5      2020-07-06 [1] CRAN (R 4.0.2)
##  readxl         1.3.1      2019-03-13 [1] CRAN (R 4.0.2)
##  rgdal        * 1.5-18     2020-10-13 [1] CRAN (R 4.0.3)
##  rgeos        * 0.5-5      2020-09-07 [1] CRAN (R 4.0.2)
##  RgoogleMaps    1.4.5.3    2020-02-12 [1] CRAN (R 4.0.2)
##  rio            0.5.16     2018-11-26 [1] CRAN (R 4.0.2)
##  rjson          0.2.20     2018-06-08 [1] CRAN (R 4.0.0)
##  rlang          0.4.8      2020-10-08 [1] CRAN (R 4.0.3)
##  rmarkdown      2.3        2020-06-18 [1] CRAN (R 4.0.2)
##  rstatix        0.6.0      2020-06-18 [1] CRAN (R 4.0.2)
##  rstudioapi     0.11       2020-02-07 [1] CRAN (R 4.0.2)
##  rvest          0.3.6      2020-07-25 [1] CRAN (R 4.0.2)
##  sandwich       2.5-1      2019-04-06 [1] CRAN (R 4.0.2)
##  scales         1.1.1      2020-05-11 [1] CRAN (R 4.0.2)
##  sessioninfo    1.1.1      2018-11-05 [1] CRAN (R 4.0.2)
##  sf           * 0.9-5      2020-07-14 [1] CRAN (R 4.0.2)
##  sjlabelled     1.1.6      2020-06-25 [1] CRAN (R 4.0.2)
##  sjmisc         2.8.5      2020-05-28 [1] CRAN (R 4.0.2)
##  sjPlot       * 2.8.4      2020-05-24 [1] CRAN (R 4.0.2)
##  sjstats        0.18.0     2020-05-06 [1] CRAN (R 4.0.2)
##  sp           * 1.4-2      2020-05-20 [1] CRAN (R 4.0.2)
##  statmod        1.4.34     2020-02-17 [1] CRAN (R 4.0.2)
##  stringi        1.5.3      2020-09-09 [1] CRAN (R 4.0.2)
##  stringr        1.4.0      2019-02-10 [1] CRAN (R 4.0.2)
##  survival       3.2-3      2020-06-13 [2] CRAN (R 4.0.2)
##  TH.data        1.0-10     2019-01-21 [1] CRAN (R 4.0.2)
##  tibble         3.0.3      2020-07-10 [1] CRAN (R 4.0.2)
##  tidyr        * 1.1.2      2020-08-27 [1] CRAN (R 4.0.2)
##  tidyselect     1.1.0      2020-05-11 [1] CRAN (R 4.0.2)
##  units          0.6-7      2020-06-13 [1] CRAN (R 4.0.2)
##  vctrs          0.3.4      2020-08-29 [1] CRAN (R 4.0.2)
```

```
##  viridisLite    0.3.0      2018-02-01 [1] CRAN (R 4.0.2)
##  webshot        0.5.2      2019-11-22 [1] CRAN (R 4.0.2)
##  withr          2.3.0      2020-09-22 [1] CRAN (R 4.0.2)
##  xfun           0.17       2020-09-09 [1] CRAN (R 4.0.2)
##  xml2           1.3.2      2020-04-23 [1] CRAN (R 4.0.2)
##  xtable         1.8-4      2019-04-21 [1] CRAN (R 4.0.2)
##  yaml           2.2.1      2020-02-01 [1] CRAN (R 4.0.2)
##  zip            2.1.1      2020-08-27 [1] CRAN (R 4.0.2)
##  zoo            1.8-8      2020-05-02 [1] CRAN (R 4.0.2)
##
## [1] C:/Users/localadmin/Documents/R/win-library/4.0
## [2] C:/Program Files/R/R-4.0.2/library
```
